# New Derivatives of 3,4-Dihydroisoquinoline-3-carboxylic Acid with Free-Radical Scavenging, d-Amino Acid Oxidase, Acetylcholinesterase and Butyrylcholinesterase Inhibitory Activity

**DOI:** 10.3390/molecules191015866

**Published:** 2014-09-30

**Authors:** Jolanta Solecka, Adam Guśpiel, Magdalena Postek, Joanna Ziemska, Robert Kawęcki, Katarzyna Łęczycka, Agnieszka Osior, Bartłomiej Pietrzak, Krzysztof Pypowski, Agata Wyrzykowska

**Affiliations:** 1 National Institute of Public Health—National Institute of Hygiene, Chocimska 24, Warsaw 00-791, Poland; E-Mails: aguspiel@pzh.gov.pl (A.G.); mpostek@pzh.gov.pl (M.P.); jziemska@pzh.gov.pl (J.Z.); 2 Institute of Chemistry, Siedlce University, 3.Maja 54, Siedlce 80-110, Poland; E-Mails: katarzyna.leczycka@icho.edu.pl (K.L.); agnieszka.osior@icho.edu.pl (A.O.); bartlomiej.pietrzak@gmail.com (B.P.); pypowski@uph.edu.pl (K.P.); agatawyrzykowska@gmail.com (A.W.)

**Keywords:** free radicals, scavenger, DAAO, AChE, BuChE, inhibitor, 3,4-dihydroisoquinoline

## Abstract

A series of 3,4-dihydroisoquinoline-3-carboxylic acid derivatives were synthesised and tested for their free-radical scavenging activity using 2,2-diphenyl-1-picrylhydrazyl radical (DPPH^·^), 2,2'-azino-bis(3-ethylbenzothiazoline-6-sulfonic acid) radical (ABTS^·+^), superoxide anion radical (O_2_^·−^) and nitric oxide radical (^·^NO) assays. We also studied d-amino acid oxidase (DAAO), acetylcholinesterase (AChE) and butyrylcholinesterase (BuChE) inhibitory activity. Almost each of newly synthesised compounds exhibited radical scavenging capabilities. Moreover, several compounds showed moderate inhibitory activities against DAAO, AChE and BuChE. Compounds with significant free-radical scavenging activity may be potential candidates for therapeutics used in oxidative-stress-related diseases.

## 1. Introduction

3,4-Dihydroisoquinoline derivatives have several important biological properties and are in the area of interest of the pharmaceutical industry. Published activities include antimetastatic [1], anti-inflammatory and analgesic [2], antiarrhythmic, and antiaggregatory [3] antihypertensive [4], and antibacterial [5]. They also inhibit catechol O-methyltransferase [6] and kinase JNK3 [7]. Most of investigated 3,4-dihydroisoquinoline derivatives have substituents at C1.

We have recently described the isolation, synthesis and antimicrobial activity of 6,7-dihydroxy-3,4-dihydroisoquinoline-3-carboxylic acid (**5e**), a C1-unsubstituted secondary metabolite of the *Streptomyces* sp. 8812 fermentation process [8,9]. This prompted us to prepare a series of hydroxy- and halogeno-substituted 3,4-dihydroisoquinoline-3-carboxylic acids and investigate their biological activity. We also included in our investigation 2-oxo-1,4,5,9b-tetrahydro-2*H*-azeto[2,1-a]isoquinoline analogue, as well as a 4,5-dihydro-3*H*-2-benzazepine analogue.

Isoquinoline alkaloid derivatives (benzyltetrahydroisoquinolines (±)-coclaurine and (±)-norarmepavine) also present antioxidative properties [10]. Berberine, with widely demonstrated antioxidative activities, exhibited acetylcholinesterase (AChE) and butyrylcholinesterase (BuChE) inhibitory activities, as well [11].

Our main interests are concerned with the determination of antioxidative properties due to the fact that phenolic compounds often present potent free radical scavenging activities. Several known, potent free-radical scavengers such as flavonols (e.g., quercetin, kaempferol), flavones (e.g., luteolin, apigenin), flavanols (e.g., catechin) and isoflavones (e.g., genistein) contain phenols and polyphenolic moiety [12]. Previous studies have shown that polyphenolic structures are more potent free-radical scavengers than those with only one hydroxyl group [13]. Also, the *ortho* location of two hydroxyl groups increases the antioxidative properties [13,14]. The free-radical scavenging potential of polyphenolic compounds is associated with their reducing (hydrogen- or electron-donating) activities [12,15].

Oxidative stress is a process that arises when free radicals and oxidants are produced in excess and cells cannot destroy them properly. In other words, an imbalance between the formation and neutralisation of free radicals can occur, owing to a depletion of antioxidants or the accumulation of reactive oxygen species (ROS), and can result in the appearance of oxidative stress [16,17,18,19]. The latter may lead to the change of cell components such as proteins, lipids, lipoproteins, nucleic acids and so forth [18,19,20].

Oxidative stress plays a crucial role in the development of various diseases such as cardiovascular disorders; atherosclerosis; hypertension or heart failure; diabetes mellitus; cancer; rheumatoid arthritis; ocular diseases; neurodegenerative diseases such as Parkinson’s and Alzheimer’s disease (AD), as well as psychic impairments such as schizophrenia and is linked to aging [15,16,17,18].

The synthesised compounds were investigated for their free-radical scavenging activity against 2,2-diphenyl-1-picrylhydrazyl radical (DPPH^·^), 2,2'-azino-bis(3-ethylbenzothiazoline-6-sulfonic acid) radical (ABTS^·+^), superoxide anion radical (O_2_^·−^) and nitric oxide radical (^·^NO). Additionally, we evaluated compounds for some activities associated with oxidative stress–inhibitory activity against d-amino acid oxidase (DAAO, a potential target in schizophrenia treatment) and for the inhibition of two enzymes important in pathophysiology of Alzheimer’s disease: acetylcholinesterase (AChE) and butyrylcholinesterase (BuChE).

## 2. Results and Discussion

### 2.1. Chemistry

**Scheme 1:**
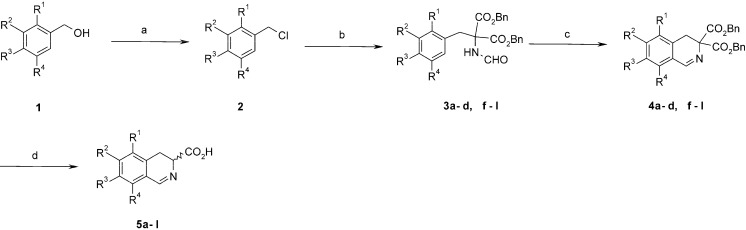
Synthesis of 3,4-dihydroisoquinoline-3-carboxylic acid derivatives.

Derivatives of the isoquinoline **5** have been obtained in a multistep synthesis using the Bischler-Napieralski reaction [21] as a cyclization step (Scheme 1, Table 1). Benzyl alcohols **1** can be easily obtained by known methods from commercially available hydroxy- or methoxy-substituted benzaldehydes. We have used benzyl protection for hydroxyl groups. Some other protective groups like allyl, carbonate or acetal groups were not stable in the cyclization step. Reduction of the carbonyl group and chlorination with thionyl chloride gave benzyl chlorides **2**. Alkylation of dibenzyl formamidomalonate with **2** gave Bischler-Napieralski reaction substrates **3**. Cyclization was conducted under standard conditions using POCl_3_ in acetonitrile at 70–95 °C. Higher reaction temperatures resulted in extensive formation of polymers and lowered the yield. Two isomers can form in the case of 2,6-unsubstituted derivatives **3b**, **d**, **g** and **h** (R^1^ = H). Usually only one product was obtained. For example cyclization of compound **3b**, gives predominantly 8-benzyloxy-7-bromo-3,4-dihydroisoquinoline instead of the corresponding 6-benzyloxy-7-bromo derivative. Apparently the C2 carbon atom in formamidomalonate **3b** is more nucleophilic than C6. The stereochemistry of **4d**, **g** and **h** has been determined on the basis of HMBC correlation spectra, and derivative **4b** showed a vicinal coupling constant between C5-H and C6-H. In the last step of the synthesis, all benzyl groups were removed with BBr_3_ [22] and decarboxylation took place gently to give acid **5** (Table 2). We have also used hydrogenation on Pd/C with 1,4-cyclohexadiene as hydrogen donor. This is very mild method which selectively removes benzyl groups in the presence of C=N bond. However formation of isoquinoline derivatives due to aromatization of the ring was noticed. More difficult was removal of methyl groups with BBr_3_. In the case of trimethoxy-substituted dihydroisoquinoline we have obtained mixture of isomers, where **5i** was the major one.

*Reagents and conditions:* (a) SOCl_2_, Py, CH_2_Cl_2_; (b) K_2_CO_3_, dibenzyl formamidomalonate, acetone; (c) POCl_3_, MeCN; (d) BBr_3_, CH_2_Cl_2_.

**Table 1:** Formamidomalonates **3** and 3,4-dihydroisoquinoline derivatives **4**.

3 (Yield,%)	4 (Yield,%)	R^1^	R^2^	R^3^	R^4^
**3a** (63)	**4a** (31)	Br	H	H	OBn
**3b** (95)	**4b** (26)	H	H	Br	OBn
**3c** (77)	**4c** (94)	H	OBn	H	OBn
**3d** (65)	**4d** (94)	H	OMe	OBn	I
**3f** (66)	**4f** (58)	OMe	OMe	H	Br
**3g** (65)	**4g** (94)	H	OMe	OMe	Br
**3h** (57)	**4h** (95)	H	OMe	OMe	Cl
**3i** (70)	**4i** (95)	H	OMe	OMe	OMe
**3j** (75)	**4j** (85)	H	OBn	Br	OBn
**3k** (88)	**4k** (94)	Cl	OBn	H	OBn
**3l** (56)	**4l** (86)	Cl	OMe	OMe	H

**Table 2:** 3,4-Dihydroisoquinoline-3-carboxylic acid derivatives.

5 (Yield,%)	R^1^	R^2^	R^3^	R^4^
**5a** (22)	**Br**	**H**	**H**	**OH**
**5b** (87)	**H**	**H**	**Br**	**OH**
**5c** (62)	**H**	**OH**	**H**	**OH**
**5d** (22)	**H**	**OH**	**OH**	**I**
**5e** ^a^	**H**	**OH**	**OH**	**H**
**5f** (21)	**OH**	**OH**	**H**	**Br**
**5g** (34)	**H**	**OH**	**OH**	**Br**
**5h** (15)	**H**	**OH**	**OH**	**Cl**
**5i** (60)	**H**	**OH**	**OMe**	**OH**
**5j** (53)	**H**	**OH**	**Br**	**OH**
**5k** (17)	**Cl**	**OH**	**H**	**OH**
**5l** (8)	**Cl**	**OH**	**OH**	**H**

^a^ Reference [9].

**Scheme 2:**
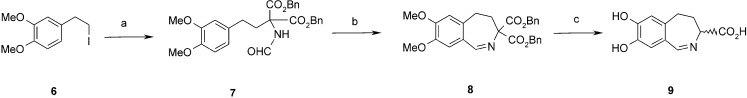
Synthesis of 4,5-dihydro-3*H*-2-benzazepin-3-carboxylic acid **9**.

**Scheme 3:**
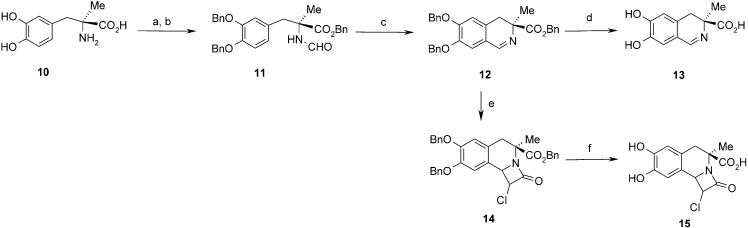
Synthesis of the 3-methyl derivative of 3,4-dihydroisoquinoline-3-carboxylic acid **13** and β-lactam derivative **15**.

The most problematic was purification of polyhydroxylated derivatives of 3,4-dihydroisoquinoline-3-carboxylic acid **5**. The products are sensitive to air and bases giving aromatic derivatives of isoquinolines. Due to the very low solubility in organic solvents and high polarity of the products, we had to use chromatography on ion exchange and polyamide columns. In a similar way, we obtained 4,5-dihydro-3*H*-2-benzazepin-3-carboxylic acid **9** from 4-(2-iodoethyl)-1,2-dimethoxybenzene (Scheme 2). Optically active 3-methyl derivative of 3,4-dihydroisoquinoline-3-carboxylic acid **13** was obtained using l-methyl-DOPA by formylation with formyloxyacetonitrile and Bischler-Napieralski cyclization. We also synthesised a β-lactam derivative of dihydroisoquinoline **15** by the Bose method using [2+2] cycloaddition with chloroketene generated *in situ* from chloroacetyl chloride [23]. This type of reaction is usually highly stereoselective. Examination of the coupling constant of C1-H (1.7 Hz) suggests that we have obtained *trans* stereoisomer. The benzyl groups in **14** were removed by hydrogenolysis to give β-lactam **15** (Scheme 3).

*Reagents and conditions*: (a) NaH, dibenzyl formamidomalonate, DMF, 43%; (b) POCl_3_, MeCN, 52%; (c) BBr_3_, CH_2_Cl_2_, 58%.

*Reagents and conditions:* (a) OHCOCH_2_CN, DMSO, 64%; (b) BnBr, K_2_CO_3_, MeCN, 48%; (c) POCl_3_, MeCN, 88%; (d) BBr_3_, CH_2_Cl_2_, 88%; (e) ClCH_2_COCl, Et_3_N, CH_2_Cl_2_, 47%; (f) H_2_, Pd/C, EtOH, 85%.

### 2.2. Biological Activities

#### 2.2.1. Free-Radical Scavenging Activity

All the synthesised derivatives were assayed *in vitro* for their antioxidant and free-radical scavenging activity. In these assays, the compounds were tested for their ability to scavenge DPPH^·^, ABTS^·+^, O_2_^·−^ and ^·^NO. The EC_50_ values are summarized in Table 3. In general, almost all of the described compounds were potent scavengers on at least one free radical used in our study. Lower EC_50_ value indicates higher radical-scavenging activity. Free-radical scavenging activities of synthesised derivatives were compared to the properties shown by naturally obtained compound **5e**.

Compounds **5e**, **5g** and **9** showed the best EC_50_ value, better than ascorbic acid on DPPH^·^ scavenging. Compounds **5l**, **5f** and **5d** also presented high DPPH^·^ scavenging activity, comparable to the standard (Table 3).

**Table 3:** Biological activities of synthesised compounds.

Tested Compound	Scavenging Activity [EC_50_] ^a^				DAAO Inhibition [IC_50_] ^c^/[%] ^d^	AChE Inhibition [IC_50_] ^c^	BuChE Inhibition IC_50_] ^c^
	DPPH^·^	ABTS^·+^	O_2_^·−^	^·^NO			
**5a**	351.60	29.62	402.62	2196.69	8.46 ^c^	-	-
**5b**	1103.08	280.47	N.D. ^b^	901.25	13.88 ^d^	N.A. ^e^	N.A.
**5c**	520.32	16.22	N.D.	2459.99	1.66 ^c^	N.A.	N.A.
**5d**	39.45	22.58	4.53	330.61	3.35 ^c^	-	-
**5e**	23.26	7.19	35.96	386.38	7.52 ^d^	0.56	0.37
**5f**	36.07	13.49	9.54	337.07	2.06 ^c^	0.54	N.A.
**5g**	26.22	11.74	14.23	402.82	2.00 ^c^	N.A.	N.A.
**5h**	52.69	21.48	116.09	3219.46	1.74 ^c^	0.82	N.A.
**5i**	63.15	23.73	35.54	2236.12	3.28 ^c^	N.A.	N.A.
**5j**	269.19	113.71	869.76	540.23	11.50 ^d^	0.47	0.64
**5k**	494.52	28.85	49.21	979.18	7.27 ^d^	0.59	1.42
**5l**	32.98	9.97	5.42	1644.66	1.29 ^c^	N.A.	0.17
**9**	27.03	12.02	5.92	N.D.	0.55 ^c^	2.08	N.A.
**13**	56.01	18.76	203.61	65.05	7.89 ^d^	1.78	0.62
**15**	84.62	10.62	4.40	1054.32	2.02 ^c^	N.A.	1.75
**Ascorbic acid**	42.11	28.25	98.77	-	-	-	-
**Trolox**	-	-	-	161.37	-	-	-
**Benzoic acid**	-	-	-	-	2.28 ^c^	-	-
**Galanthamine hydrobromide**	-	-	-	-	-	0.001	0.009

^a^ The values were expressed as μM concentration; ^b^ N.D.—not determined; ^c^ The values were expressed as mM concentration; ^d^ % of DAAO inhibition at 3.57 mM concentration; ^e^ N.A.—not active.

**Figure 1:**
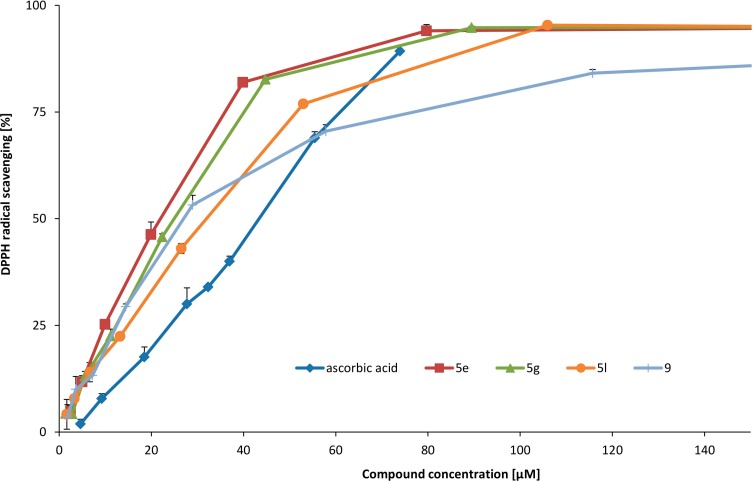
DPPH^·^ scavenging of the most active compounds.

Compounds **5h** and **13**, also showed good activity (slightly lower than standard). The presence of two hydroxyl groups at 6- and 7-position (**5e**, **5g**, **5l**, **5d**, **5h**, **13**) and respectively at 7- and 8-position for compound **9** resulted in significant DPPH radical scavenging activity. *Meta* location of hydroxyl groups (compounds **5i**, **5j**, **5k**, **5c**) did not result in better activities in comparison to **5e** (*ortho* location). Among compounds with *meta* location of hydroxyl groups (compounds **5i**, **5j**, **5k**, **5c)**, compound **5i** with an electron-donating group at 7-position presented better activity than others. Introduction of a halogen atom at 5- or 8-position of a compound, with two hydroxyl groups at 6- and 7-positions (**5g**, **5l**, **5d**, **5h**), did not potentiate scavenging activity on DPPH^·^ in comparison to **5e**. DPPH radical was scavenged by compounds **5e**, **5g**, **9**, **5l** and ascorbic acid in a concentration-dependent manner (Figure 1).

**Figure 2:**
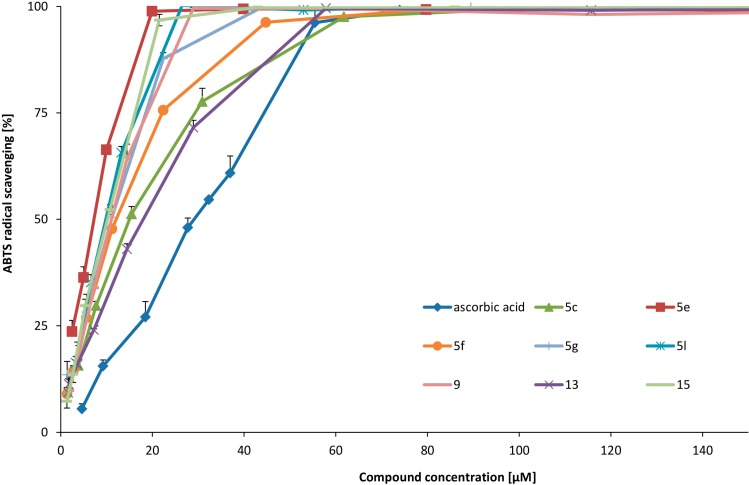
ABTS^·+^ scavenging of the most active compounds.

Compound **5e**, was the most active scavenger on ABTS^·+^. The EC_50_ value of the above-mentioned compound was approximately one quarter of that of ascorbic acid. Also, **5l**, **15**, **5g**, **9**, **5f**, **5c**, **13**, **5h**, **5d**, **5i** **and** **5k** showed very potent activities. The presence of two hydroxyl groups at the 6- and 7-positions (**5e**, **5l**, **5g**, **13**, **5h**, **5d**) and respectively at 7- and 8-positions in **15** and **9**, resulted in significant ABTS^·+^ scavenging activity of the compounds. Halogen atoms (chlorine or bromine) at the 5- and 8-positions did not significantly influence the activity in comparison to **5e**. ABTS^·+^ was scavenged by compounds **5e**, **5l**, **15**, **5g**, **9**, **5f**, **5c**, **13** and ascorbic acid in a concentration-dependent manner (Figure 2).

**Figure 3:**
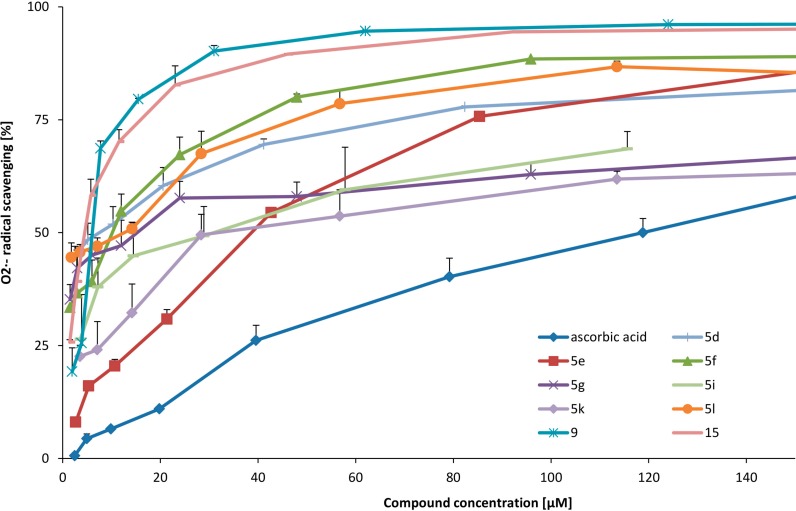
O_2_^·−^ scavenging of the most active compounds.

We obtained nine compounds (**15**, **5d**, **5l**, **9**, **5f**, **5g**, **5i**, **5e**, **5k**) with superoxide anion radical (O_2_^·−^) scavenging activities better than the standard (ascorbic acid). Compounds **15**, **5d**, **5l**, **9**, and **5f** presented very high superoxide anion radical scavenging activities. Their EC_50_ values were 22-times lower (**15** and **5d**) and more than 10-times lower (**5l**, **9**, **5f**) than the EC_50_ value for ascorbic acid. The chloroazetidinone moiety at [2,1a]-position in compound **15** and methyl group at the 4-position improved the activity in comparison to compound **5e**. Also, the presence of two hydroxyl groups at the 6- and 7-positions and iodine at the 8-position (**5d**) or chlorine at the 5-position (**5l**) caused significant O_2_^·−^ scavenging activity. *Ortho* position of two hydroxyl groups in the tested compounds contributed to significant superoxide anion radical scavenging activity. A bromine atom at the 8-position (compound **5g**) enhanced O_2_^·−^ scavenging activity of the compound, in comparison to a chlorine atom at the same position (compound **5h**). The EC_50_ value for compound **5g** was about sevenfold lower than the EC_50_ value for ascorbic acid. This data suggests that substitution of phenolic compounds with a halogen atom (I > Br > Cl) stabilizes the superoxide anion radical. Compound **9**, with a dihydrobenzoazepine ring, showed 6-times greater O_2_^·−^ scavenging activity in comparison to the similar compound, **5e**, with a dihydropyridine ring. Tested compounds were able to scavenge superoxide anion radicals in a concentration-dependent manner (Figure 3).

Compound **13** was the most potent ^·^NO scavenger. Its EC_50_ value was lower than the standard (Trolox). All of the other tested compounds showed rather moderate scavenging activities on the nitric oxide radical. Comparing the results of all above assays, in general, compounds with two hydroxyl groups (**5c**–**l**, **9**, **13**, **15**) were more potent free-radical scavengers than phenolic compounds (**5a**, **5b**). Also, an *ortho* position of hydroxyl groups determined more significant free radical scavenging activity than the *meta* position.

#### 2.2.2. DAAO Inhibitory Activity

**Figure 4:**
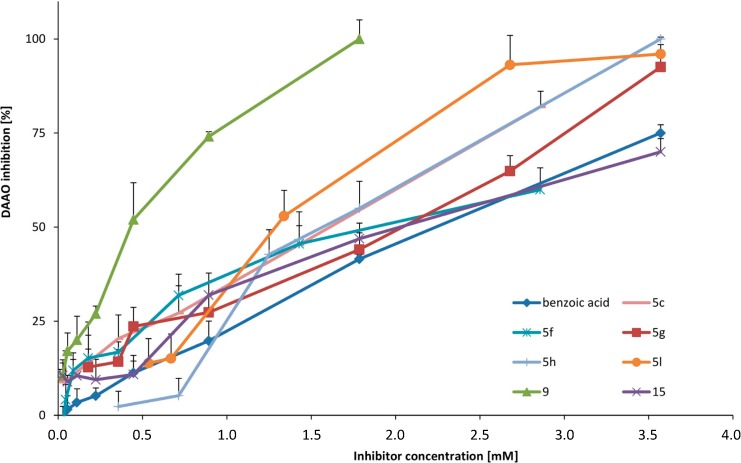
DAAO inhibition of the most active compounds.

Similarly, all newly synthesised compounds were screened for DAAO inhibitory activity. Compound **9** was the most active inhibitor of DAAO (Table 3). It showed a fourfold lower IC_50_ value than the standard benzoic acid. Other compounds, including **5l**, **5c**, **5h**, **5g**, **15** and **5f**, showed comparable or better DAAO inhibitory activities than the standard. The presence of two hydroxyl groups at the *meta* position in the benzene ring (**5c**) as well as the *ortho* location of hydroxyl groups with additional chlorine or bromine atom at 5- or 8-positions (**5g**, **5h**, **5l**, **5f**) exhibited good activity. Comparison of the compound with two hydroxyl groups at 6- and 7-positions (**5e**) and compounds with an additional halogen atom at the 8-position (**5g**, **5h**, **5d**) showed that halogen atoms enhanced DAAO inhibition. Moreover, the more electronegative the substituent (Cl > Br > I) at the 8-position, the stronger the resulting DAAO inhibitor. Compounds **5a**–**5l** and **9** are obtained as racemic mixtures. Biological activities of separated enantiomers may be higher (even two times). However, the activity would remain in milimolar concentration range. Synthesised derivatives inhibited DAAO in a concentration-dependent manner (Figure 4).

#### 2.2.3. AChE Inhibitory Activity

The effect of the synthesised compounds on AChE activity was evaluated, using a selective AChE inhibitor, galanthamine hydrobromide as a standard. Only a few of the synthesised compounds (**5j**, **5f**, **5e**, **5k**) showed rather weak activity against AChE. None of the tested compounds were more potent than the standard. The results are presented in Table 3.

#### 2.2.4. BuChE Inhibitory Activity

The reported compounds were tested for their BuChE inhibitory activity (IC_50_ values are summarised in Table 3). They showed moderate activities (**5l**, **5e**, **13**, **5j**) against BuChE.

## 3. Experimental Section

### 3.1. General Information

d-Amino acid oxidase from porcine kidney (DAAO), d-alanine, nitroblue tetrazolium chloride (NBT), 2,2-diphenyl-1-picrylhydrazyl (DPPH^·^), 2,2'-azino bis(3-ethylbenzothiazoline-6-sulfonic acid) (ABTS^·+^), potassium persulphate, sodium nitroprusside, Griess reagent, acetylthiocholine iodide, acetylcholinesterase from *Electrophorus electricus* (AChE), 5,5-dithiobis-2-nitrobenzoic acid (DTNB), galanthamine hydrobromide, butyrylcholinesterase from equine serum (BuChE) and butyrylthiocholine iodide were obtained from Sigma-Aldrich (Poznań, Poland) and 2,4-dinitro phenylhydrazine was purchased from POCH S.A. (Gliwice, Poland). Dibenzyl formamidomalonate has been prepared analogously to the procedure described for diethyl derivative [24]. All others reagents were of standard analytical grade.

NMR spectra were recorded using Varian 400 MR (400 MHz) spectrometer. Chemical shifts of aqueous solutions were referenced against solvent signal (4.75 ppm). Most of the ^13^C-NMR chemical shifts of deprotected phenols **5** could not be determined due to low solubility and deuteriation in the ring. IR spectra were recorded on Shimadzu IRAffinity-1S spectrophotometer using single reflection ATR (ZnSe). Mass spectra were obtained using LTQ Orbitrap Velos (Thermo Scientific) and QToF Premier (Waters), HR and LR respectively. The radical scavenging activity and enzymes inhibitory activity were assessed by UV-VIS spectrophotometer (FLUOstar Omega BMG LABTECH microplate reader). Copies of ^1^H and ^13^C-NMR spectra are available in the Supplementary Information file.

### 3.2. Synthesis and Characterisation

#### 3.2.1. General Procedure for the Synthesis of Benzyl Chlorides **2**

Pyridine (0.36 mL) and thionyl chloride (0.37 mL) were added dropwise to a solution of protected benzyl alcohol **1** (4.78 mmol) in CH_2_Cl_2_ (15 mL). The resultant mixture was then stirred at rt for 2.5 h. The reaction was quenched with 10 mL of water and stirred for 0.5 h to destroy excess of thionyl chloride. The organic layer was removed, and the aqueous layer was extracted twice with CH_2_Cl_2_ (3 × 15 mL). The combined organic layers were dried over MgSO_4_ and evaporated. The products were used in the next step without purification.

#### 3.2.2. General Procedure for Alkylation of Dibenzyl Formamidomalonate **3a**–**3l**

Dibenzyl formamidomalonate (315 mg, 0.96 mmol) followed by K_2_CO_3_ (1.76 g, 12.75 mmol) and KI (478 mg, 2.88 mmol) were added to a solution of respective benzyl chlorides (0.96 mmol) in acetone (20 mL). The reaction mixture was stirred under reflux (oil bath, 60 °C) for 20 h. When complete, the reaction mixture was cooled and filtered through Celite. The solvent was evaporated to give a solid.

*Dibenzyl 2-(2-bromo-5-benzyloxybenzyl)-2-formamidomalonate* (**3a**). The compound was purified by chromatography on silica gel (EtOAc-hexane, 1:3). White crystals. Yield 0.363 g (63%). ^1^H-NMR (CDCl_3_) δ: 3.84 (s, 2H, CH_2_); 4.98 (s, 2H, OCH_2_Ph); 5.12 and 5.16 (AB, *J =* 14, 4H, CH_2_Ph); 6.58 (bs, 1H, NH); 6.69 (d, *J =* 3.3 Hz, 1H, C6-H); 6.76 (dd, *J =* 3.3, 8.8 Hz, 1H, C4-H); 7.40 (d, *J =* 8.8 Hz; 1H, C3-H); 7.16–7.38 (m, 15H, Ar); 7.94 (d, *J =* 1.3 Hz, 1H, CHO). ^13^C-NMR (CDCl_3_) δ: 37.6; 65.5; 68.5; 70.0; 116.0; 116.1; 118.7; 127.2; 128.1; 128.3; 128.5; 128.55; 128.6; 133.7; 134.5; 135.4; 136.5; 157.6; 160.1; 166.8. HR MS ESI calculated for C_32_H_29_BrNO_6_ (M+H) 602.1173. Found 602.1170.

*Dibenzyl 2-(3-benzyloxy-4-bromobenzyl)-2-formamidomalonate* (**3b**). This compound was used in the next step without purification. Beige solid. Yield 95%. ^1^H-NMR (CDCl_3_) δ: 3.84 (s, 2H, CH_2_); 4.98 (s, 2H, OCH_2_Ph); 5.12 and 5.16 (AB, *J =* 12, 4H, CH_2_Ph); 6.76 (dd, *J =* 2.9 Hz, *J =* 8.8, 1H, C6-H); 6.89 (d, *J =* 2.9, 1H, C2-H); 6.60 (bs, 1H, NH); 7.14–7.38 (m, 15H, Ar); 7.40 (d, *J =* 8.8, 1H, C5-H); 7.93 (d, *J =* 0.8 Hz, 1H, CHO). ^13^C-NMR (CDCl_3_) δ: 37.6; 65.5; 68.4; 69.9; 116.04; 116.05 118.6; 127.2; 128.0; 128.3; 128.5; 128,6; 133.7; 134.4; 135.4; 136.5; 157.6; 160.1, 166.8. HR MS ESI calculated for C_32_H_29_BrNO_6_ (M+H) 602.1172. Found 602.1170.

*Dibenzyl (3,5-dibenzyloxybenzyl)-2-formamidomalonate* (**3c**). The product was purified on silica gel (EtOAc-hexane 1:2.5). White solid. Yield 77%. ^1^H-NMR (CDCl_3_) δ: 3.59 (s, 2H, CH_2_); 4.93 (s, 4H, OCH_2_Ph); 5.08 and 5.12 (AB, *J =* 12 Hz, 4H, CH_2_Ph); 6.17 (d, *J =* 2.1 Hz, 2H, C2-H, C6-H); 6.52 (t, *J =* 2.1 Hz, 1H, C4-H); 6.55 (bs, 1H, NH); 7.16–7.44 (m, 20H, Ar); 7.91 (d, *J =* 0.8 Hz, 1H, CHO). ^13^C-NMR (CDCl_3_) δ: 38.2; 66.7; 68.3; 69.9; 101.2; 109.2; 127.3; 127.9; 128.3; 128.5; 128.61; 128.62; 134.5; 136.7; 136.8; 159.7; 159.8; 166.6. HR MS ESI calculated for C_39_H_36_NO_7_ (M+H) 630.2486. Found 630.2466.

*Dibenzyl 2-(3-iodo-4-benzyloxy-5-methoxybenzyl)-2-formamidomalonate* (**3d**). This compound was used in the next step without purification. Beige solid. Yield 65%. ^1^H-NMR (CDCl_3_) δ: 3.63 (s, 2H, CH_2_); 3.67 (s, 3H, OCH_3_); 4.97 (s, 2H, OCH_2_Ph); 5.13 and 5.20 (AB, *J =* 12.1 Hz, 4H, CH_2_Ph); 6.51 (d, *J =* 1.8 Hz, 1H, C6-H); 6.80 (bs, 1H, NH); 6.99 (d, *J =* 1.8 Hz, 1H, C2-H); 7.24–7.40 (m, 13H, Ar); 7.53–7.57 (m, 2H, Ar); 8.20 (d, *J =* 1.1 Hz, 1H, CHO). ^13^C-NMR (CDCl_3_) δ: 37.2; 55.91; 55.94; 66.8; 68.5; 74.4; 92.8; 114.8; 128.0; 128.3; 128.37; 128.4; 128.69; 128.74; 131.6; 132.7; 134.4; 137.0; 147.2; 152.4; 159.9; 166.5. HR MS ESI calculated for C_33_H_30_INO_7_ (M+H) 680.1139. Found 680.1131.

*Dibenzyl 2-(5-bromo-2,3-dimethoxybenzyl)-2-formamidomalonate* (**3f**). The product was purified on silica gel (EtOAc-hexane 1:2). White solid. Yield 66%. ^1^H-NMR (CDCl_3_) δ: 3.66 (s, 2H, CH_2_); 3.66 (s, 3H, OCH_3_); 3.82 (s, 3H, OCH_3_); 5.13 and 5.17 (AB, *J =* 12 Hz, 4H, OCH_2_Ph); 6.67 (bs, 1H, NH); 6.75 (d, *J =* 2.5 Hz, 1H, C6-H); 6.93 (d, *J =* 2.5 Hz, 1H, C4-H); 7.21–7.38 (m, 10H, Ar); 8.12 (d, *J =* 1.3 Hz, 1H, CHO). ^13^C-NMR (CDCl_3_) δ: 33.0; 55.9; 60.6; 65.8; 68.4; 115.2; 116.1; 126.3; 128,2; 128.5; 128.6; 130.1; 134.6; 147.3; 153.3; 160.2; 166.9. HR MS ESI calculated for C_27_H_27_BrNO_7_ (M+H) 556.0965. Found 556.0960. 

*Dibenzyl 2-(3-bromo-4,5-dimethoxybenzyl)-2-formamidomalonate* (**3g**). The product was purified on silica gel (EtOAc-hexane 1:2). White solid. Yield 65%. ^1^H-NMR (CDCl_3_) δ: 3.62 (s, 2H, CH_2_); 3.66 (s, 3H, OCH_3_); 3.81 (s, 3H, OCH_3_); 5.12 and 5.20 (AB, *J =* 12 Hz, 4H, OCH_2_Ph); 6.44 (d, *J =* 2.1 Hz, 1H, C2-H); 6.71 (d, *J =* 2.1 Hz, 1H, C6-H); 6.76 (bs, 1H, NH); 7.24–7.38 (m, 10H, Ar); 8.20 (d, *J =* 0.8 Hz, 1H, CHO). ^13^C-NMR (CDCl_3_) δ: 37.4; 55.9; 60.5; 66.8; 68.5; 113.6; 117.5; 125.8; 128.4; 128.7; 128,8; 131.7; 134.4; 145.8; 153.4; 159.9; 166.5. HR MS ESI calculated for C_27_H_27_BrNO_7_ (M+H) 558.0965. Found 558.0935.

*Dibenzyl 2-(3-chloro-4,5-dimethoxybenzyl)-2-formamidomalonate* (**3h**). This compound was purified on silica gel (EtOAc-hexane 1:2.5). White solid. Yield 57%. ^1^H-NMR (CDCl_3_) δ: 3.62 (s, 2H, CH_2_); 3.67 (s, 3H, OCH_3_); 3.82 (s, 3H, OCH_3_); 5.12 and 5.20 (AB, *J =* 12.1 Hz, 2H, OCH_2_Ph); 6.40 (d, *J =* 1.7 Hz, 1H, C6-H); 6.52 (d, *J =* 1.7 Hz, 1H, C2-H); 6.76 (s, 1H, NH); 7.25–7.37 (m, 10H, Ar); 8.20 (d, *J =* 1.3 Hz, 1H, CHO). ^13^C-NMR (CDCl_3_) δ: 37.5; 56.0; 60.6; 66.7; 68.5; 112.9; 123.0; 128.1; 128.4; 128.7; 128.8; 131.0; 134.4; 144.8; 153.5; 159.9; 166.6. HR MS ESI calculated for C_27_H_27_ClNO_7_ (M+H) 512.1470. Found 512.1467.

*Dibenzyl 2-(3,4,5-trimethoxybenzyl)-2-formamidomalonate* (**3i**). This compound was purified on silica gel (EtOAc-hexane 1:1.5). White solid. Yield 70%. ^1^H-NMR (CDCl_3_) δ: 3.63 (s, 6H, OCH_3_); 3.67 (s, 2H, CH_2_); 3.80 (s, 3H, OCH_3_); 5.10 and 5.20 (AB, *J =* 12.1 Hz, 4H, OCH_2_PH); 6.16 (s, 2H, C2-H, C6-H); 6.73 (bs, 1H, NH); 7.23–7.36 (m, 10H, Ar); 8.19 (d, *J =* 1.2 Hz, 1H, CHO). ^13^C-NMR (CDCl_3_) δ: 38.3; 55.9; 60.8; 66.9; 68.3; 106.9; 128.2; 128.67; 128.68; 130.1; 134.5; 137.3; 153.0; 159.8; 166.8. HR MS ESI calculated for C_28_H_30_NO_8_ (M+H) 508.1965. Found 508.1945.

*Dibenzyl 2-(4-bromo-3,5-dibenzyloxybenzyl)-2-formamidomalonate* (**3j**). This compound was purified on silica gel (EtOAc-hexane 1:4). Beige solid. Yield 75%. ^1^H-NMR (CDCl_3_) δ: 3.58 (s, 2H, CH_2_); 5.00 (s, 4H, OCH_2_Ph); 5.02 and 5.05 (AB, *J =* 12.1 Hz, 4H, CH_2_Ph); 6.17 (s, 2H, C2-H, C6-H); 6.34 (bs, 1H, NH); 7.14–7.42 (m, 20H, Ar); 7.68 (d, *J =* 1.2 Hz, 1H, CHO). ^13^C-NMR (CDCl_3_) δ: 38.2; 66.6; 68.3; 70.6; 101.7; 108.5; 126.7; 127.8; 128.1; 128.5; 128.6; 128.7; 134.4; 134.9; 136.5; 155.9; 159.9; 166.4. HR MS ESI calculated for C_39_H_35_BrNO_7_ (M+H) 708.1591. Found 708.1593.

*Dibenzyl (2-chloro-3,5-dibenzyloxybenzyl)-2-formamidomalonate* (**3k**). This compound was used in the next step without purification. Beige solid. Yield 88%. ^1^H-NMR (CDCl_3_) δ: 3.87 (s, 2H, CH_2_); 4.94 (s, 2H, OCH_2_Ph); 5.07 (s, 2H, OCH_2_Ph); 5.11 and 5.16 (AB, *J =* 12.5 Hz, 4H, CH_2_Ph); 6.30 (d, *J =* 2.9 Hz, 1H, C4-H); 6.56 (br, 1H, NH); 6.57 (d, *J =* 2.9 Hz, 1H, C6-H); 7.18–7.45 (m, 20H, Ar); 7.88 (d, *J =* 1.3, 1H, CHO). ^13^C-NMR (CDCl_3_) δ: 35.7; 65.5; 68.4; 70.1; 70.9; 101.7; 109.7; 116.3; 127.1; 127.2; 128.0; 128.1; 128.3; 128.5; 128.56; 128.59; 128.6; 134.47; 134.52; 136.2; 136.5; 155.0; 157.3; 160.1; 166.9. HR MS ESI calculated for C_39_H_35_ClNO_7_ (M+H) 664.2096. Found 664.2079.

*Dibenzyl 2-(2-chloro-3,4-dimethoxybenzyl)-2-formamidomalonate* (**3l**). This compound was purified on silica gel (EtOAc-hexane 1:2.5). White solid. Yield 56%. ^1^H-NMR (CDCl_3_) δ: 3.81 (s, 3H, OCH_3_); 3.82 (s, 2H, CH_2_); 3.83 (s, 3H, OCH_3_); 5.15 (s, 4H, CH_2_Ph); 6.63 and 6.70 (AB, *J =* 8.3 Hz, 2H, C6-H and C5-H); 6.66 (br, 1H, NH); 7.21–7.34 (m, 10H, Ar); 8.13 (s, 1H, CHO). ^13^C-NMR (CDCl_3_) δ: 35.1; 55.9; 60.5; 65.7; 68.4; 110.3; 125.4; 126.9; 128.4; 128.6; 129.6; 134.5; 145.4; 152.9; 154.4; 160.1; 166.9. HR MS ESI calculated for C_27_H_27_ClNO_7_ (M+H) 512.1470. Found 512.1448.

#### 3.2.3. General Procedure for Bischler-Napieralski Cyclization. Preparation of Dibenzyl 3,4-Dihydro- isoquinolin-3,3-dicarboxylates **4a**–**4l**

POCl_3_ (0.23 mL) was added to a stirred solution of the respective dibenzyl formamidomalonate **3a**–**3l** (0.83 mmol) in acetonitrile (10 mL) and the mixture was heated under various conditions depending on the compound. The solvent was evaporated and residue was made alkaline with aqueous NaHCO_3_ (5 mL). The mixture was extracted with CH_2_Cl_2_ (3 × 10 mL). The combined organic layers were dried with MgSO_4_ and concentrated to give the product.

*Dibenzyl 5-bromo-8-benzyloxy-3,4-dihydroisoquinolin-3,3-dicarboxylate* (**4a**). The reaction mixture was heated at 80 °C (temp. of oil bath) for 8 h. The compound was purified by chromatography on silica gel (EtOAc-hexane, 1:3). Brown oil. Yield 0.150 g (31%). ^1^H-NMR (CDCl_3_) δ: 3.44 (s, 2H C4-H); 5.12 (s, 2H, OCH_2_Ph); 5.10 and 5.20 (AB, *J =* 12.1 Hz, 4H, CH_2_PH); 6.76 (d, *J =* 8.7 Hz, 1H, C7-H); 7.48 (d, *J =* 8.7 Hz, 1H, C6-H); 7.14–7.43 (m, 15H, Ar); 8.95 (s, 1H, C1-H). ^13^C-NMR (CDCl_3_) δ: 30.8; 67.8; 70.3; 70.6; 113.2; 114.5; 118.8; 126.9; 127.1; 128.2; 128.3; 128.4; 128.5; 128.7; 134.4; 135.1; 135.8; 136.5; 155.7; 157.7; 168.4. MS ESI calculated for C_32_H_27_BrNO_5_ (M+H) 586.1. Found 586.1.

*Dibenzyl 8-benzyloxy-7-bromo-3,4-dihydroisoquinolin-3,3-dicarboxylate* (**4b**). The reaction mixture was heated at 73 °C for 15 h. The product was purified on silica gel (EtOAc-hexane 1:6). Brown oil. Yield 100 mg (26%). ^1^H-NMR (CDCl_3_) δ: 3.44 (s, 2H C4-H); 5.11 (s, 2H, OCH_2_Ph); 5.10 and 5.21 (AB, *J =* 12.5 Hz, 4H, CH_2_PH); 6.76 (d, *J =* 8.7 Hz, 1H, C5-H); 7.19–7.40 (m, 15H, Ar); 7.47 (d, *J =* 8.7 Hz, 1H, C6-H); 8.94 (s, 1H, C1-H). ^13^C-NMR (CDCl_3_) δ: 30.8; 67.8; 70.3; 70.6; 113.2; 114.5; 118.8; 127.1; 128.0; 128.2; 128.3; 128.4; 128.7; 134.4; 135.1; 135.8; 136.5; 155.7; 157.7; 168.4. MS ESI calculated for C_32_H_27_NO_5_Br (M+H) 584.1. Found 583.9.

*Dibenzyl 6.8-dibenzyloxy-3,4-dihydroisoquinolin-3,3-dicarboxylate* (**4c**). The reaction mixture was heated at 70 °C for 8 h. Product was purified by extraction with hot cyclohexane. Orange solid. Yield 94%. ^1^H-NMR (CDCl_3_) δ: 3.31 (s, 2H C4-H); 5.00 (s, 2H, OCH_2_Ph); 5.07 (s, 2H, OCH_2_Ph); 5.10 and 5.19 (AB, *J =* 12.5 Hz, 4H, CH_2_Ph); 6.33 (d, *J =* 2.1 Hz, 1H, C8-H); 6.42 (d, *J =* 2.1 Hz, 1H, C5-H); 7.20–7.44 (m, 20H, Ar); 8.87 (s, 1H, C1-H). ^13^C-NMR (CDCl_3_) δ: 31.7; 67.8; 68.3; 70.3; 99.1; 106.0; 109.1; 127.1; 127.6; 128.0; 128.2; 128.4; 128.7; 128.74; 134.4; 135.1; 135.8; 136.9; 158.0; 158.1. HR MS EI calculated for C_39_N_33_NO_6_ 611.2308. Found 611.2292.

*Dibenzyl 6-methoxy-7-benzyloxy-8-iodo-3,4-dihydroisoquinolin-3,3-dicarboxylate* (**4d**). The reaction mixture was heated at 80 °C for 9 h. The product was purified on silica gel (EtOAc-hexane 1:3). Brown oil. Yield 94%. ^1^H-NMR (CDCl_3_) δ: 3.30 (s, 2H, C4-H); 3.86 (s, 3H, OCH_3_); 4.97 (s, 2H, OCH_2_Ph); 5.14 and 5.19 (AB, *J =* 12.1 Hz, 4H, CH_2_Ph); 6.63 (s, 1H); 7.17–7.21 (m, 4H, Ar); 7.26–7.29 (m, 5H, Ar); 7.34–7.42 (m, 4H); 7.55–7.58 (m, 2H); 8.65 (s, 1H, C1-H). ^13^C-NMR (CDCl_3_) δ: 31.3; 56.1; 67.8; 70.3; 74.5; 98.3; 111.6; 122.4; 128.0; 128.18; 128.2; 128.3; 128.43; 128.44; 132.6; 135.1; 136.7; 147.0; 155.4; 165.2; 168.6. HR MS EI calculated for C_33_H_29_INO_6_ 662.1034. Found 662.1037.

*Dibenzyl 8-bromo-5,6-dimethoxy-3,4-dihydroisoquinolin-3,3-dicarboxylate* (**4f**). The reaction mixture was heated at 95 °C for 4 h. The product was purified on silica gel (EtOAc-hexane 1:3). Beige oil. Yield 58%. ^1^H-NMR (CDCl_3_) δ: 3.36 (s, 2H, C4-H); 3.67 (s, 3H, OCH_3_); 3.88 (s, 3H, OCH_3_); 5.14 and 5.17 (AB, *J =* 12.1 Hz, 4H, CH_2_Ph); 6.95 (s, 1H, C7-H); 7.18–7.30 (m, 10H, Ar); 8.72 (s, 1H, C1-H). ^13^C-NMR (CDCl_3_) δ: 25.4; 56.0; 60.6; 67.7; 70.1; 114.9; 119.0; 119.7; 128.0; 128.2; 128.4; 129.3; 135.1; 145.2; 155.9; 160.8; 168.5. MS ESI calculated for C_27_H_25_BrNO_6_ (M+H) 538.1. Found 538.1.

*Dibenzyl 8-bromo-6,7-dimethoxy-3,4-dihydroisoquinolin-3,3-dicarboxylate* (**4g**). The reaction mixture was heated at 80 °C for 9 h. Product was used in the next step without purification. Brown oil. Yield 94%. ^1^H-NMR (CDCl_3_) δ: 3.30 (s, 2H, C4-H); 3.83 (s, 3H, OCH_3_); 3.86 (s, 3H, OCH_3_); 5.14 and 5.19 (AB, *J =* 12.1 Hz, 4H, CH_2_Ph); 6.59 (s, 1H, C5-H); 7.15–7.36 (m, 10H, Ar); 8.78 (s, 1H, C1-H). ^13^C-NMR (CDCl_3_) δ: 31.2; 56.1; 60.6; 67.8; 70.2; 110.5; 119.7; 119.8; 128.0; 128.2; 128.4; 132.1; 135.1; 145.7; 156.3; 160.8; 168.6. HR MS EI calculated for C_27_H_24_BrNO_6_ 537.0787. Found 537.0774.

*Dibenzyl 8-chloro-6,7-dimethoxy-3,4-dihydroisoquinolin-3,3-dicarboxylate* (**4h**). The reaction mixture was heated at 70 °C for 8 h. Product was used in the next step without purification. Brown oil. Yield 95%. ^1^H-NMR (CDCl_3_) δ: 3.31 (s, 2H, C4-H); 3.84 (s, 3H, OCH_3_); 3.87 (s, 3H, OCH_3_); 5.14 and 5.20 (AB, *J =* 12.1 Hz, 4H, CH_2_Ph); 6.56 (s, 1H, C5-H); 7.19–7.38 (m, 10H, Ar); 8.85 (s, 1H, C1-H). ^13^C-NMR (CDCl_3_) δ: 31.1; 56.1; 60.7; 67.8; 70.2; 109.8; 118.4; 128.0; 128.2; 128.3; 128.4; 131.4; 135.1; 144.6; 156.5; 158.5; 168.6. HR MS ESI calculated for C_27_H_25_ClNO_6_ (M+H) 494.1365. Found 494.1360.

*Dibenzyl 6,7,8-trimethoxy-3,4-dihydroisoquinolin-3,3-dicarboxylate* (**4i**). The reaction mixture was heated at 70 °C for 9 h. Product was used in the next step without purification. Brown oil. Yield 95%. ^1^H-NMR (CDCl_3_) δ: 3.66 (s, 2H, C4-H); 3.83 (s, 3H, OCH_3_), 3.99 (s, 3H, OCH_3_); 4.17 (s, 3H, OCH_3_); 5.20 and 5.27 (AB, *J =* 12.1 Hz, 4H, CH_2_Ph); 6.53 (s, 1H, C5-H); 7.21–7.39 (m, 10H, Ar); 9.02 (s, 1H, C1-H). ^13^C-NMR (CDCl_3_) δ: 32.4; 57.3; 61.5; 61.6; 66.3; 69.9; 107.4; 110.3; 128.6; 128.7; 128.9; 133.2; 133.8; 139.7; 157.0; 161.5; 163.8; 165.0. HR MS ESI calculated for C_28_H_28_NO_7_ (M+H) 490.1860. Found 490.1841.

*Dibenzyl 7-bromo-6,8-dibenzyloxy-3,4-dihydroisoquinolin-3,3-dicarboxylate* (**4j**). The reaction mixture was heated at 80 °C for 7 h. Product was used in the next step without purification. Brown oil. Yield 85%. ^1^H-NMR (CDCl_3_) δ: 3.29 (s, 2H C4-H); 4.84 (s, 2H, OCH_2_Ph); 5.15 (s, 2H, OCH_2_Ph); 5.15 and 5.18 (AB, *J =* 12.5 Hz, 4H, CH_2_Ph); 6.55 (s, 1H, C5-H); 7.10–7.55 (m, 20H, Ar); 8.69 (s, 1H, C1-H). ^13^C-NMR (CDCl_3_) δ: 31.4; 53.4; 67.8; 70.3; 71.1; 106.5; 108.5; 116.4; 127.0; 128.1; 128.26; 128.3; 128.4; 128.44; 128.57; 128.61; 128.7; 135.0; 135.1; 135.6; 135.8; 156.1; 157.8; 159.0; 168.6. HR MS ESI calculated for C_39_H_33_BrNO_6_ (M+H) 690.1485. Found 690.1464.

*Dibenzyl 5-chloro-6,8-dibenzyloxy-3,4-dihydroisoquinolin-3,3-dicarboxylate* (**4k**). The reaction mixture was heated at 75 °C for 10 h. Product was used in the next step without purification. Brown oil. Yield of crude product 94%. ^1^H-NMR (CDCl_3_) δ: 3.75 (s, 2H, C4-H); 5.18 and 5.26 (AB, *J =* 12.1 Hz, 4H, CH_2_Ph); 5.20 (s, 2H, OCH_2_Ph); 5.21 (s, 2H, OCH_2_Ph); 6.49 (s, 1H, C7-H); 7.22–7.46 (m, 20H, Ar); 9.07 (s, 1H, C1-H). HR MS EI calculated for C_39_H_33_ClNO_6_ 646.1991. Found 646.1978.

*Dibenzyl 5-chloro-6,7-dimethoxy-3,4-dihydroisoquinolin-3,3-dicarboxylate* (**4l**). The reaction mixture was heated at 75 °C for 10 h. Product was used in the next step without purification. Brown oil. Yield of crude product 86%. ^1^H-NMR (CDCl_3_) δ: 3.44 (s, 2H, C4-H); 3.88 (s, 3H, OCH_3_); 3.89 (s, 3H, OCH_3_); 5.09 and 5.22 (AB, *J =* 12.1 Hz, 2H, CH_2_Ph); 6.84 (s, 1H, C8-H); 7.15–7.40 (m, 10H, Ar); 8.43 (s, 1H, C1-H). HR MS ESI calculated for C_27_H_25_ClNO_6_ (M+H) 494.1365. Found 494.1352.

#### 3.2.4. General Procedure for Removal of Benzylic Groups. Preparation of 3,4-Dihydroisoquinolin-3-carboxylic Acids **5a**–**5l**

BBr_3_ (0.2 mL, 2.13 mmol) was added dropwise under argon to a solution of respective dibenzyl 3,4-dihydroisoquinolin-3,3-dicarboxylate **4a**–**4l** (0.17 mmol) in CH_2_Cl_2_ (10 mL) at −20 °C. The reaction mixture was stirred for 0.5 h at the same temperature and quenched with H_2_O (25 mL). The resultant mixture was then stirred at rt for 0.5 h. The water layer was separated and washed 3 times with CH_2_Cl_2_. The solution was concentrated under vacuum at rt and the residue was loaded on a column packed with ion exchange resin Dowex 50W X4 (*ca*. 30 mL, pretreated subsequently with water, 2 M HCl, and water to pH 6–7). The column was washed with water (200 mL), and the product was eluted with 2 M aqueous NH_3_. Brown to yellow colored fractions were collected. Ammonia was removed under water pump vacuum at rt and the solution was evaporated (water bath temp. 35 °C). The residue was acidified with 2 M aqueous HCl to pH 2, and purified on polyacrylamide (Biogel P-2) using 0.1% aqueous TFA.

*5-Bromo-8-hydroxy-3,4-dihydroisoquinolin-3-carboxylic acid* (**5a**). Beige powder. Yield 10 mg (22%). ^1^H-NMR (D_2_O/DCl) δ: 3.25 and 3.35 (ABX, *J =* 7.1 Hz, 8.8 Hz, 17.5 Hz, 2H, C4-H); 4.48 (t, *J =* 7.1 Hz, 1H, C3-H); 6.58 (d, *J =* 9.6 Hz, 1H, C7-H); 7.59 (d, *J =* 9.6 Hz, 1H, C6-H); 8.73 (s, 1H, C1-H). MS ESI calculated for C_10_H_9_NO_3_Br (M+H) 269.9. Found 269.9.

*7-Bromo-8-hydroxy-3,4-dihydroisoquinolin-3-carboxylic acid* (**5b**). Beige powder. Yield 87%. ^1^H-NMR (D_2_O/DCl) δ: 3.27 and 3.36 (ABX, *J =* 7.1 Hz, 8.8 Hz, 17.5 Hz, 2H, C4-H), 4.50 (t, *J =* 7.9 Hz, C3-H); 6.62 (d, *J =* 9.1, 1H, C5-H), 7.62 (d, *J =* 9.1, 1H, C6-H), 8.77 (s, 1H, C1-H). IR: 579; 617; 665; 698; 723; 750; 781; 804; 841; 897; 937; 993; 1040; 1084; 1136; 1184; 1234; 1277; 1292; 1310; 1344; 1364; 1400; 1452; 1601; 1634; 2250–3500 br; 2806; 3044; 3129. MS ESI calculated for C_10_H_9_NO_3_Br (M+H) 270.0. Found 269.9.

*6,8-Dihydroxy-3,4-dihydroisoquinolin-3-carboxylic acid* (**5c**). Beige powder. Yield 62%. ^1^H-NMR (D_2_O-H_2_O/DCl) δ: 3.19 and 3.31 (ABX, *J =* 7.0 Hz, 9.2 Hz, 16.9 Hz, 2H, C3-H); 4.44 (m, 1H, C3-H); 6.13 (d, *J =* 1.5 Hz, 1H, C7-H); 6.26 (s, 1H, C6-H); 8.64 (s, 1H, C1-H). IR: 586; 619; 633; 662; 704; 733; 770; 808; 843; 870; 901; 957; 986; 1015; 1069; 1113; 1169; 1188; 1202; 1250; 1314; 1348; 1385; 1404; 1504; 1541; 1582; 1609; 2250–3300 br; 2519; 2612; 2689; 2722; 2793; 3071; 3250. MS ESI calculated for C_10_H_10_NO_4_ (M+H) 208.0. Found 208.0.

*6,7-Dihydroxy-8-iodo-3,4-dihydroisoquinolin-3-carboxylic acid* (**5d**). The reaction mixture was kept at rt for 24 h. Beige powder. Yield 22%. ^1^H-NMR (DMSO) δ: 3.23 and 3.31 (ABX, *J =* 7.5; 8.3; 17.5 Hz, 2H, C4-H); 4.82 (t, *J =* 7.9 Hz, 1H, C3-H); 6.59 (s, 1H, C5-H); 8.48 (s, 1H, C1-H); 11.9 (bs, 1H, OH). IR: 598; 648; 667; 692; 741; 768; 795; 822; 870; 885; 908; 943; 966; 1013; 1032; 1057; 1128; 1177; 1192; 1219; 1256; 1281; 1298; 1371; 1485; 1508; 1557; 1587; 1634; 1721; 2250–3650 br; 2826; 2911; 2959; 3146. MS ESI calculated for C_10_H_9_INO_4_ (M+H) 333.9. Found: 333.9.

*6,7-Dihydroxy-3,4-dihydroisoquinolin-3-carboxylic acid* (**5e**). This compound was obtained from l-DOPA according to published procedure [9]. ^1^H-NMR (D_2_O-H_2_O/DCl) δ: 3.28 and 3.38 (ABX, *J =* 17.0, 8.6, 7.7, Hz, 1H, C4-H); 4.56 (dd, *J =* 8.6 Hz, 7.7 Hz, 1H, C3-H); 6.91 (s, 1H, C5-H); 7.28 (s, 1H, C8-H); 8.64 (s, 1H, C1-H). IR: 586; 629; 648; 691; 725; 752; 773; 802; 843; 853; 883; 908; 937; 982; 1013; 1130; 1177; 1196; 1258; 1288; 1371; 1393; 1429; 1531; 1557; 2250–3500 br; 3038.

*8-Bromo-5,6-dihydroxy-3,4-dihydroisoquinolin-3-carboxylic acid* (**5f**). The reaction mixture was kept at rt for 24 h. Beige powder. Yield 21%. ^1^H-NMR (D_2_O-H_2_O/DCl) δ: 3.13 and 3.21 (ABX, *J =* 16.9, 8.8, 7.0 Hz, 2H, C4-H); 4.30 (t, *J =* 8.1 Hz, 1H, C3-H) 6.73 (s, 1H, C7-H); 8.42 (s, 1H, C1-H). ^13^C-NMR (CDCl_3_) δ: 23.9; 54.9; 108.8; 120.1; 121.4; 126.2; 143.2; 158.4; 175.9. IR: 615; 669; 694; 735; 770; 797; 824; 853; 891; 962; 1009; 1096; 1128; 1202; 1219; 1234; 1298; 1348; 1377; 1404; 1420; 1474; 1499; 1560; 1595; 1719; 2250–3500 br; 2970; 3038; 3134. MS ESI calculated for C_10_H_8_BrNO_4_ (M+H) 286.0. Found 286.0.

*8-Bromo-6,7-dihydroxy-3,4-dihydroisoquinolin-3-carboxylic acid* (**5g**). The reaction was kept at rt for 24 h. Beige powder. Yield 34%. ^1^H NMR (D_2_O-H_2_O/DCl) δ: 3.32 and 3.43 (ABX, *J =* 16.5, 8.8, 7.0 Hz, 2H, C4-H); 4.53 (t, *J =* 8.8 Hz, 1H, C3-H); 6.59 (s, 1H, C5-H); 8.64 (s, 1H, C1-H). ^13^C-NMR (CDCl_3_) δ: 32.7; 114.1; 114.9; 115.8; 133.9; 142.4; 155.7; 163.9; 170.3. IR: 600; 667; 696; 721; 762; 799; 839; 893; 1040; 1088; 1136; 1182; 1236; 1258; 1279; 1292; 1344; 1368; 1393; 1557; 1593; 2250–3500 br; 2806; 3044; 3125. MS ESI calculated for C_10_H_8_BrNO_4_ (M+H) 286.0. Found 286.0.

*8-Chloro-6,7-dihydroxy-3,4-dihydroisoquinolin-3-carboxylic acid* (**5h**). The reaction mixture was kept at rt for 16 h. Beige powder. Yield 15%. ^1^H-NMR (D_2_O-H_2_O/DCl) δ: 3.15 and 3.25 (ABX, *J =* 7.1 Hz, *J =* 9.2 Hz, *J =* 17.1 Hz, 2H, C4-H); 4.37 (t, *J =* 7.9 Hz, 1H, C3-H); 6.41 (s, 1H, C5-H); 8.50 (s, 1H, C1-H). IR: 600; 640; 665; 696; 719; 735; 824; 851; 876; 908; 943; 968; 989; 1032; 1047; 1105; 1134; 1179; 1194; 1215; 1234; 1256; 1265; 1325; 1379; 1393; 1454; 1470; 1487; 1557; 1595; 1611; 1682; 1730; 2851; 2916; 2957; 3225; 3294. HR MS ESI calculated for C_10_H_9_ClNO_4_ (M+H) 242.0215. Found: 242.0213.

*6,8-Dihydroxy-7-methoxy-3,4-dihydroisoquinolin-3-carboxylic acid* (**5i**). The reaction mixture was kept at rt for 24 h. Product **5i** could not be purified from 6- and 8-methoxy isomers. Beige powder. Yield 61 mg. Major isomer (60%). ^1^H-NMR (D_2_O-H_2_O/DCl) δ: 3.10 (m, 2H, C4-H), 3.82 (s, 3H, OCH_3_), 4.30 (m, 1H, C3-H), 6.33 (s, 1H, C5-H), 8.39 (s, 1H, C1-H). IR: 554; 600; 635; 671; 756; 777; 800; 901; 949; 991; 1080; 1098; 1138; 1202; 1265; 1304; 1354; 1373; 1454; 1522; 1589; 2250–3650 br; 2835; 3030; 3179. HR MS ESI calculated for C_11_H_12_NO_5_ (M+H) 238.0709. Found: 238.0702.

*7-Bromo-6,8-dihydroxy-3,4-dihydroisoquinolin-3-carboxylic acid* (**5j**). Beige powder. Yield 53%. ^1^H-NMR (D_2_O-H_2_O/DCl) δ: 2.99 and 3.11 (ABX, *J =* 6.7, 9.6, 16.2 Hz, 2H, C4-H); 4.25 (dd, *J =* 9.6, 6.7 Hz; 1H, C3-H); 6.00 (s, 1H, C5-H); 8.33 (s, 1H, C1-H). ^13^C-NMR (D_2_O-H_2_O/DCl) δ: 27.7; 53.8; 97.5; 106.4; 109.0; 137.8; 159.6; 159.7; 164.5; 171.3. IR: 590; 660; 691; 746; 764; 797; 826; 854; 872; 945; 1011; 1030; 1061; 1096; 1123; 1173; 1229; 1246; 1283; 1329; 1369; 1514; 1589; 2250–3500 br; 2970; 3144. MS ESI calculated for C_10_H_9_NO_4_Br (M+H) 286.0. Found 285.9.

*5-Chloro-6,8-dihydroxy-3,4-dihydroisoquinolin-3-carboxylic acid* (**5k**). The reaction mixture was kept at −20 °C for 1 h. Beige powder. Yield 17%. ^1^H-NMR (D_2_O-H_2_O/DCl) δ: 3.22 and 3.29 (ABX, *J =* 7.8, *J =* 8.6, 18.0 Hz, 2H, C4-H); 4.69 (ddd, *J =* 1.3, 7.8, 8.6 Hz, 1H, C3-H); 6.25 (s, 1H, C7-H); 8.68 (d, *J =* 1.3 Hz, 1H, C1-H). IR: 598; 633; 650; 671; 743; 781; 829; 858; 939; 1001; 1036; 1094; 1186; 1215; 1248; 1285; 1319; 1337; 1398; 1420; 1504; 1531; 1557; 2250–3650 br; 2602; 2745; 3065. MS ESI calculated for C_10_H_9_ClNO_4_ (M+H) 242.0. Found: 241.9.

*5-Chloro-6,7-dihydroxy-3,4-dihydroisoquinolin-3-carboxylic acid* (**5l**). The reaction mixture was kept at rt for 21 h. Beige powder. Yield 8%. ^1^H-NMR (D_2_O-H_2_O/DCl) δ: 2.77 and 2.92 (ABX. *J =* 7.9 Hz, *J =* 10.0, 17.5 Hz, 2H, C4-H); 4.42 (ddd, *J =* 7.9, 10.0, 1.7 Hz, 1H, C3-H); 6.64 (s, 1H, C8-H); 8.20 (d, *J =* 1.5 Hz, 1H, C1-H). ^13^C-NMR (D_2_O-H_2_O/DCl) δ: 53.4; 114.8; 119.9; 127.6; 143.8; 150.6; 150.7; 164.3; 169.7. IR: 606; 631; 654; 689; 718; 731; 758; 800; 826; 853; 868; 907; 930; 951; 976; 1020; 1142; 1171; 1254; 1265; 1308; 1342; 1396; 1429; 1454; 1472; 1514; 1557; 1593; 1639; 2250–3500 br; 2731; 2920; 3063; 3146; 3566. MS ESI calculated for C_10_H_9_ClNO_4_ (M+H) 242.0. Found 241.9.

#### 3.2.5. Preparation of Dibenzyl 2-[2-(3,4-Dimethoxyphenyl)ethyl]-2-formamidomalonate (**7**)

Solution of dibenzyl formamidomalonate (1.629 g, 4.98 mmol) in anh. DMF (15 mL) was added to a suspension of NaH (299 mg, 7.46 mmol, 60% in oil) in anh. DMF (10 mL). The reaction mixture was stirred for 1 h at rt. Next, a solution of 4-(2-iodoethyl)-1,2-dimethoxybenzene (1.53 g, 4.98 mmol) in anh. DMF (10 mL) was added and the reaction mixture was heated at 60 °C for 20 h. The solvent was distilled off under vacuum. Water (100 mL) was carefully added and the mixture was extracted with CH_2_Cl_2_ (3 × 50 mL). Collected extracts were dried over MgSO_4_ and evaporated. Product was purified by crystallization from diethyl ether cooling the solution to −20 °C. Orange solid. Yield 1.05 g (43%). ^1^H-NMR (CDCl_3_) δ: 2.34 (m, 2H, ArCH_2_CH_2_); 2.70 (m, 2H, ArCH_2_CH_2_); 3.79 (s, 3H, OCH_3_); 3.83 (s, 3H, OCH_3_); 5.08 and 5.14 (AB, *J =* 12.1 Hz, 4H, CH_2_Ph); 6.51–6.55 (m, 2H, C2,6-H); 6.72 (d, *J =* 8.0 Hz, C5-H); 6.95 (s, 1H, NH); 7.22–7.35 (m, 10H, Ar), 8.18 (d, *J =* 1.3 Hz, 1H, CHO). ^13^C-NMR (CDCl_3_) δ: 29.4; 33.9; 55.8; 55.9; 65.9; 68.3; 111.1; 111.6; 120.3; 128.3; 128.5; 128.6; 132.6; 134.6; 147.4; 148.8; 159.7; 167.2. MS ESI calculated for C_28_H_29_NO_7_ (M+H) 492.1. Found: 492.2.

#### 3.2.6. Preparation of Dibenzyl 7,8-Dimethoxy-4,5-dihydro-3H-2-benzazepine-3,3-dicarboxylate (**8**)

This compound was obtained according to the procedure 3.2.3. The reaction mixture was heated at 80 °C for 20 h. The product was purified by dissolving in boiling cyclohexane and cooling to 10 °C. Brown oil. Yield 52%. ^1^H-NMR (CDCl_3_) δ: 2.68 (m, 2H, C5-H); 2.85 (m, 2H, C4-H); 3.86 (s, 3H, OCH_3_); 3.87 (s, 3H, OCH_3_); 5.09 and 5.18 (AB, *J =* 12.5 Hz, 4H, CH_2_Ph); 6.58 (s, 1H, C7-H); 6.84 (s, 1H, C10-H); 7.20–7.30 (m, 10H, Ar); 8.41 (s, 1H, C1-H). ^13^C-NMR (CDCl_3_) δ: 30.6; 33.1; 55.9; 56.0; 67.5; 75.8; 112.2; 116.4; 125.4; 128.0; 128.1; 128.3; 134.8; 135.2; 147.2; 150.8; 163.7; 169.1. HR MS EI calculated for C_28_H_27_NO_6_ 473.1838. Found: 473.1852.

#### 3.2.7. Preparation of 7,8-Dihydroxy-4,5-dihydro-3H-2-benzazepine-3-carboxylic Acid (**9**)

This compound was obtained according to the procedure 3.2.4. The reaction mixture was kept at rt for 24 h. Brown solid. Yield 58%. ^1^H-NMR (D_2_O-H_2_O/DCl) δ: 2.07–2.18 (m, 2H, C4-H); 2.70 and 2.86 (m, 2H, C5-H); 4.62 (t, *J =* 5.3 Hz, 1H, C3-H); 6.61 (s, 1H, C6-H); 6.99 (s, 1H, C9-H); 8.11 (s, 1H, C1-H). IR: 552; 565; 594; 667; 694; 752; 804; 843; 881; 941; 1038; 1103; 1138; 1171; 1238; 1252; 1281; 1373; 1456; 1506; 1558; 1576; 1601; 2250–3500 br; 2805; 2870; 3042; 3123. MS ESI calculated for C_11_H_12_NO_4_ (M+H) 222.0. Found 222.0.

#### 3.2.8. Preparation of (*S*)-Benzyl 2-(3,4-Dibenzyloxy)benzyl-2-formamidopropanoate (**11**)

Formylation of l-methyl-DOPA. Formyloxyacetonitrile (1.326 g, 15.6 mmol) was added to a solution of l-methyl-DOPA hydrate, (1.38 g, 5.8 mmol) in DMSO (10 mL) and the mixture was stirred at rt for 3 days. Solvent was removed under vacuum and the residue was dissolved in acetonitrile (5 mL). Dichloromethane (150 mL) and hexane (10 mL) were added to precipitate product. After filtration white solid was obtained (0.88 g). Yield 64%. ^1^H-NMR (DMSO) δ: 1.32 (s, 3H, CH_3_), 2.94 and 2.98 (AB, *J =* 13.3 Hz, 2H, CH_2_), 6.36 (dd, J_1_=1.9, 7.9 Hz, 1H, C6-H), 6.51 (d, *J =* 1.9 Hz, 1H, C2-H), 6.60 (d, *J =* 7.9 Hz, 1H, C5-H), 7.90 (br, 1H, NH), 7.93 (d, *J =* 1.7 Hz, 1H, CHO); 8.70 (br, 1H, OH). ^13^C-NMR (DMSO) δ: 22.7; 58.9; 115.1; 117.8; 117.8; 121.2; 127.2; 144.0; 144.7; 160.8; 174.6.

Benzylation of l-*N*-formylmethyl-DOPA. K_2_CO_3_, (12.4 g) and benzyl bromide (10.74 g, 62.8 mmol) were added to a solution of l-*N*-formylmethyl-DOPA (4.29 g, 17.9 mmol) in anh. acetonitrile (80 mL). The reaction mixture was stirred and heated at 60 °C for 16 h. The solvent was evaporated and the water was added to the residue. The mixture was extracted with CH_2_Cl_2_ (3 × 20 mL). Combined extracts were dried with Na_2_SO_4_ and evaporated. Product was purified on silica gel (EtOAc-CH_2_Cl_2_ 1:1). White solid. Yield 244 mg (48%). ^1^H-NMR (CDCl_3_) δ: 1.68 (s, 3H, CH_3_), 3.10 (d, *J =* 13.8 Hz, 1H, CH_2_), 3.45 (d, *J =* 13.8 Hz, 1H, CH_2_), 5.05–5.14 (m, 6H, CH_2_Ph), 6.06 (bs, 1H, NH), 6.47 (dd, *J =* 8.3, 2.1 Hz, 1H, C6-H), 6.63 (d, *J =* 2.1 Hz, 1H, C2-H), 6.75 (d, *J =* 8.3 Hz, 1H, C5-H), 7.28–7.46 (m, 15H, Ar), 7.87 (d, *J =* 2.1 Hz, 1H, CHO). ^13^C-NMR (CDCl_3_) δ: 23.5; 40.9; 61.4; 67.6; 71.0; 71.2; 114.7; 117.0; 122.7; 127.1; 127.2; 127.3; 127.7; 127.8; 128.3; 128.4; 128.6; 128.7; 129.1; 134.9; 137.3; 148.1; 148.3; 160.4; 173.2. HR MS ESI calculated for C_32_H_32_NO_5_ (M+H) 510.2275. Found 510.2252.

#### 3.2.9. Preparation of (*S*)-Benzyl 6,7-Dibenzyloxy-3-methyl-3,4-dihydroisoquinolin-3-carboxylate (**12**)

This compound was obtained according to the procedure 3.2.3. The reaction mixture was heated at 60 °C (oil bath) for 1.5 h. The product was purified on silica gel (first CH_2_Cl_2_ then CH_2_Cl_2_-EtOAc 1:1). Orange oil. Yield 1.479 g (88%). ^1^H-NMR (CDCl_3_) δ: 1.45 (s, 3H, CH_3_), 2.75 (d, 1H, *J =* 16.3 Hz, C4-H), 3.16 (d, 1H, *J =* 16.3 Hz, C4-H), 5.15 (s, 2H, CH_2_Ph); 5.17 (s, 2H, CH_2_Ph); 5.18 (s, 2H, CH_2_Ph); 6.72 (s, 1H, C5-H); 6.92 (s, 1H, C8-H); 7.25–7.45 (m, 15H, Ar); 8.22 (s, 1H, C1-H). ^13^C-NMR (CDCl_3_) δ: 23.6; 33.8; 62.7; 66.8; 71.0; 71.6; 113.8; 114.5; 120.7; 127.2; 127.3; 127.9; 127. 94; 128.0; 128.0; 128.2; 128.4; 128.5; 128.6; 135.9; 136.5; 136.9; 147.8; 152.0; 159.0; 174.5. HR MS ESI calculated for C_32_H_30_NO_4_ (M+H) 492.2169. Found 492.2169.

#### 3.2.10. Preparation of (*S*)-6,7-Dihydroxy-3-methyl-3,4-dihydroisoquinolin-3-carboxylic Acid (**13**)

This compound was obtained according to the procedure 3.2.4. Green powder. Yield 88%. ^1^H-NMR (D_2_O-H2O/DCl) δ: 1.62 (s, 3H, CH_3_); 3.12 and 3.36 (AB, *J =* 17.1 Hz, 2H, C4-H); 6.79 (s, 1H, C5-H); 7.16 (s, 1H, C8-H); 8.57 (s, 1H, C1-H). ^13^C-NMR (D_2_O-H_2_O/DCl) δ: 21.8; 34.0; 60.8; 115.2; 116.0; 120.7; 131.7; 144.1; 155.8; 164.2; 173.8. IR: 554; 596; 627; 650; 719; 750; 797; 835; 883; 941; 966; 1126; 1180; 1233; 1277; 1331; 1381; 1447; 1516; 1557; 1568; 1614; 1634; 1643; 1667; 1732; 2250–3500 br; 2787; 2945; 3123; 3196. HR MS ESI calculated for C_11_H_11_NO_4_Na (M+Na) 244.0580. Found 244.0589.

#### 3.2.11. Preparation of Benzyl 1-Chloro-7,8-dibenzyloxy-4-methyl-2-oxo-1,4,5,9b-tetrahydro-2*H*-azeto[2,1-a]isoquinoline-4-carboxylate (**14**)

Et_3_N (654 mg, 6.48 mmol) and solution of chloroacetyl chloride (549 mg, 4.86 mmol) in CH_2_Cl_2_ (2 mL) were added to a stirred solution of benzyl 6,7-dibenzyloxy-3,4-dihydroisoquinolin-3-methyl-3-carboxylate (400 mg, 0.81 mmol) in CH_2_Cl_2_ (10 mL). The reaction mixture was stirred at rt for 20 h and quenched with aqueous NaHCO_3_ (2 mL). Stirring was continued for next 1 h. Water layer was separated and extracted with CH_2_Cl_2_. Organic layers were dried over MgSO_4_, evaporated and purified by chromatography on silica gel (acetone/CH_2_Cl_2_ 150:1). Brown oil. Yield 140 mg (47%). ^1^H-NMR (CDCl_3_) δ: 1.88 (s, 3H, CH_3_); 2.94 and 3.14 (AB, *J =* 15.8, 2H, C5-H); 4.41 (d, *J =* 1.7, 1H, C1-H); 4.69 (br, 1H, C9b-H); 5.09 (^1^/_2_AB, *J =* 12.1, 1H, CH_2_Ph); 5.10 (s, 2H, OCH_2_Ph); 5.12 (s, 2H, OCH_2_Ph); 5.13 (^1^/_2_AB, *J =* 12.1, 1H, CH_2_Ph); 6.64 (s, 1H, C6-H); 6.75 (s, 1H, C9-H); 7.13–7.46 (m, 15H, Ar). ^13^C-NMR (CDCl_3_) δ: 22.3; 39.3; 60.3; 60.4; 61.2; 67.3; 71.2; 71.4; 111.6; 115.4; 124. 2; 124.9; 127.2; 127.4; 127.8; 127.9; 128.0; 128.4; 128.5; 135.1; 136.6; 136.7; 148.5; 149.0; 162.9; 171.3. HR MS ESI calculated for C_34_H_31_ClNO_5_ (M+H) 568.1813. Found: 568.1887.

#### 3.2.12. Preparation of 1-Chloro-7,8-dihydroxy-4-methyl-2-oxo-1,4,5,9b-tetrahydro-2*H*-azeto[2,1-a]-isoquinoline-4-carboxylic Acid (**15**)

Pd/C (10%, 15 mg) was added to a solution of benzyl 1-chloro-7,8-dibenzyloxy-4-methyl-2-oxo-1,4,5,9b-tetrahydro-2*H*-azeto[2,1-a]isoquinoline-4-carboxylate **14** (90 mg, 0.16 mmol) in EtOH (7 mL) and a balloon with hydrogen was connected. The suspension was stirred for 3 h. The catalyst was centrifuged and washed two times with EtOH (3 mL). Collected solution were evaporated and dried under vacuum. White solid. Yield 40 mg (85%). ^1^H-NMR (acetone-*d*_6_) δ: 1.80 (s, 3H, CH_3_); 2.94 and 3.13 (AB, *J =* 15.4 Hz, 2H, C5-H); 4.70 (br, 1H, C9b-H); 4.74 (d, *J =* 2.1 Hz, 1H, C1-H); 6.69 (s, 1H, C6-H); 6.77 (s, 1H, C9-H); 8.10 (br, 1H, OH); 8.15 (br, 1H, OH). ^13^C-NMR (acetone-*d*_6_) δ: 22.5; 39.5; 60.7; 61.1; 62.1; 112.8; 116.8; 124.1; 124.7; 145.5; 146.1; 163.3; 173.3. HR MS ESI calculated for C_13_H_12_ClNO_5_ (M+H) 298.0404. Found: 298.0478.

### 3.3. Free-Radical Scavenging Properties

#### 3.3.1. DPPH Radical Scavenging Assay

The radical-scavenging activity of tested compounds against DPPH^·^ was determined by Williams’ method [25]. Tested compounds dissolved in water or methanol (30 μL) were added at various concentrations (to final concentration of 2, 4, 8, 16, 32, 64, 128, 256, 512 μg/mL) to freshly prepared 0.22 mM DPPH^·^ in methanol (120 μL). The absorbance was measured against the control in the microplate reader at 517 nm after 30 min of incubation at room temperature (rt). The control contained all reagents without the tested compound. The corresponding blank reading was also taken and the remaining DPPH^·^ was calculated. Each sample was replicated three times. Ascorbic acid was used as a standard.

#### 3.3.2. ABTS Radical Scavenging Assay

The radical-scavenging activity of tested compounds against ABTS^·+^ and the generation of the ABTS^·+^ radical was performed according to methods described previously [26,27]. Briefly, an ABTS^·+^ solution was prepared by mixing 2 mM ABTS (50 mL) with 70 mM potassium persulphate (200 μL), both in deionized water, and used after 2 h of being kept in the dark at rt. The ABTS^·+^ solution (40 μL) was added to 0.1 M phosphate buffer pH 7.4 (60 μL) and tested compounds (25 μL) were dissolved in water or methanol at various concentrations (to final concentration of 2, 4, 8, 16, 32, 64, 128, 256, 512 μg/mL). The absorbance was measured against the control in the microplate reader at 734 nm after 30 min of incubation at rt. The control contained all reagents without the tested compound. The corresponding blank reading was also taken and the remaining ABTS^·+^ was calculated. Each sample was replicated three times. Ascorbic acid was used as a standard.

#### 3.3.3. Superoxide Anion Radical Scavenging Assay

Superoxide scavenging assay was based on inhibition of the formazan dye formation. O_2_^·−^ was generated in alkaline DMSO by the addition of sodium hydroxide to air-saturated pure DMSO [28]. The generated superoxide reacts with NBT to give coloured diformazan. To the reaction mixture containing NBT (10 μL; 1 mg/mL solution in DMSO) and the various concentration of scavenger (30 μL; to final concentration of 2, 4, 8, 16, 32, 64, 128, 256, 512 μg/mL), alkaline DMSO (100 μL) was added to give a final volume of 140 μL per well in a microplate. The absorbance was measured in the microplate reader at 560 nm after 30 min of incubation at rt [29]. The same procedure was repeated for the control in which DMSO was added instead of scavenger solution. Each sample was replicated three times and the corresponding blank reading was also taken. Ascorbic acid was used as a standard.

#### 3.3.4. Nitric Oxide Radical Scavenging Assay

Nitric oxide was generated from sodium nitroprusside and measured by the Griess reaction [26]. Scavengers of ^·^NO compete with oxygen leading to reduced production of nitric oxide. 10 mM sodium nitroprusside in 0.1 M phosphate buffer pH 7.4 (30 μL) was mixed with tested compounds dissolved in water or methanol (25 μL) at various concentrations (to final concentration of 2, 4, 8, 16, 32, 64, 128, 256, 512 μg/mL) and incubated at 25 °C for 5 h. After incubation, Griess reagent (50 μL) was added. The absorbance was measured in the microplate reader at 546 nm against a control (without tested compounds) treated in the same way with Griess reagent. The corresponding blank reading was also taken. Each sample was replicated three times. Trolox was used as a standard.

#### 3.3.5. Effective Concentration of Scavengers (EC_50_)

The percentage of radical scavenging was calculated using Equation (1) (DPPH^·^, ABTS^·+^ and ^·^NO scavenging assays). The absorbance for the control is *A_c_* and the absorbance in the presence of the compounds or other scavenger is *A_s_*:

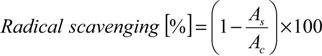
(1)

The percentage of O_2_^·−^ scavenging was calculated using Equation (2). The absorbance for the control is *A_c_* and the absorbance in the presence of the compounds or other scavenger is *A_s_*:

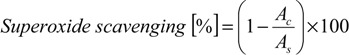
(2)

The amount of tested compound needed to scavenge free radicals by 50%, EC_50_ (effective concentration), was calculated by the linear regression between scavenger concentration and percentage of scavenging and expressed as μM.

### 3.4. DAAO Inhibitory Activity

Inhibition of DAAO was based on the determination of the α-keto acid obtained from the reaction between a DAAO and d-Ala according to the described method [30]. The method was adapted to microtitre plates and a total volume of 210 μL per well. Inhibitor solution in DMSO (15 μL), 5 mM d-Ala in 0.2 M Tris-HCl buffer pH 8.2 (121 μL) and 10 U/mL DAAO solution in 0.2 M Tris-HCl buffer pH 8.2 (5 μL) were mixed and incubated at 37 °C for 30 min. After incubation, 1 mM 2,4-dinitro phenylhydrazine dissolved in 1 M HCl (14 μL) was added to the well, mixed and further incubated at 37 °C for 10 min. Next, 1.5 M NaOH (55 μL) was added and incubated for 10 min. The absorbance was read at 445 nm against a blank sample consisting the same assay mixture but without the substrate (using Tris-HCl buffer instead of d-Ala). The positive control of the enzyme activity consisted of DMSO (15 μL) instead of inhibitor solution and the analogous blank contained Tris-HCl buffer instead of d-Ala. Sample, control and related blanks were carried out under the same condition. Benzoic acid was used as a standard. Eight different solutions of inhibitor were examined: 0.0005, 0.0025, 0.005, 0.00625, 0.0125, 0.025, 0.0375 and 0.05 M. For each of these inhibitor concentrations percentage of inhibition was calculated by using Equation (3). The absorbance for the control is *A_c_* and the absorbance in the presence of the compounds or other inhibitor is *A_i_*:

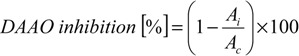
(3)

The inhibitor concentration needed to inhibit DAAO by 50% (IC_50_) was calculated by the linear regression between inhibitor concentration and percentage of inhibition. Each sample was replicated three times.

### 3.5. AChE Inhibitory Activity

The assay was based on Ellman’s method [31] with modifications. AChE activity was measured by the determination of a yellow colour produced from acetylthiocholine iodide when it reacted with the dithiobisnitrobenzoate ion. The assay was performed using microtitre plates and a total volume of 100 µL per well. In a 96-well plate, 0.1 M pH 7.4 phosphate buffer (32 µL), a serially diluted solution of tested compounds (5 µL), 0.59 U/mL AChE solution (4 µL) and 0.3 mM DTNB (42 µL) were mixed and pre-incubated at 25 °C for 2 min. Then, the substrate, 0.4mM acetylthiocholine iodide (17 µL), was added to the well and mixed. The enzymatic reaction was followed for 10 min, with a measurement every 12 s. Changes in the absorbance at 412 nm were detected in the microplate reader. The absorbance was measured against a blank sample containing the same assay mixture but without an enzyme (using phosphate buffer instead of AChE). The positive control of the enzyme activity consisted of DMSO (1.5 μL) instead of inhibitor solution. The sample, control and related blanks were measured under the same conditions. Galanthamine hydrobromide was used as a standard. Each sample was replicated three times. The concentration of the compounds that caused 50% inhibition of AChE activity (IC_50_) was calculated using linear regression analysis. At least three concentrations of tested compounds were assayed. For each of these inhibitor concentrations, the percentage of inhibition was calculated using Equation (4). The difference in absorbance for the inhibitor at *t* = 10 min and *t* = 0 is denoted Δ*A*_i_. The difference in absorbance for the control at *t* = 10 min and *t* = 0 is denoted Δ*A*_c_:

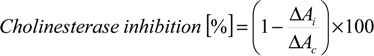
(4)

### 3.6. BuChE Inhibitory Activity

The assay was based on Ellman’s method [31] with modifications. BuChE activity was measured by the determination of the yellow colour produced from butyrylthiocholine iodide when it reacted with the dithiobisnitrobenzoate ion. The assay was performed using microtitre plates and a total volume of 100 µL per well. In a 96-well plate, 0.1 M pH 7.4 phosphate buffer (35 µL), a serially diluted solution of tested compounds (5 µL), 1.485 U/mL BuChE solution (5 µL) and 0.3 mM DTNB (42 µL) were mixed and pre-incubated at 25 °C for 2 min. Then, the substrate, 0.6 mM butyrylthiocholine iodide (13 µL), was added to the well and mixed. The enzymatic reaction was followed for 10 min, with a measurement every 12 s. Changes in absorbance at 412 nm were detected in the microplate reader. The absorbance was measured against a blank sample containing the same assay mixture, but without an enzyme (using phosphate buffer instead of BuChE). The positive control for enzyme activity contained DMSO (1.5 μL) instead of inhibitor solution. The sample, control and related blanks were measured under the same conditions. Galanthamine hydrobromide was used as a standard. Each sample was replicated three times. The concentration of the compounds that caused 50% inhibition of BuChE activity (IC_50_) was calculated using linear regression analysis. At least three concentrations of the tested compounds were assayed. For each of these inhibitor concentrations, the percentage of inhibition was calculated using Equation (4).

## 4. Conclusions

We investigated newly synthesised compounds (hydroxy- and halogeno-substituted derivatives of 3,4-dihydroisoquinoline-3-carboxylic acid, 2-oxo-1,4,5,9b-tetrahydro-2*H*-azeto[2,1-a]isoquinoline analogue and 4,5-dihydro-3*H*-2-benzazepine analogues) for their free-radical-scavenging activity and DAAO, AChE, BuChE inhibitory activity. The obtained dihydroxy-substituted derivatives of 3,4-dihydroisoquinoline-3-carboxylic acid were found to be more potent scavengers of DPPH^·^, ABTS^·+^, O_2_^·−^ and ^·^NO than monohydroxy-substituted derivatives. The presence of *ortho*-dihydroxy structures determined higher free-radical scavenging activities than the *meta* location of these substituents. These compounds were shown to be potent free-radical scavengers on at least one free radical used in this study. Obtained results are significant due to the fact that the 3,4-dihydroisoquinoline derivatives have not been described so far as potent free radical scavengers.

In conclusion, the potent free-radical scavengers obtained in the study may be potential candidates for therapeutics used in oxidative-stress-related diseases. The synthesised inhibitors of evaluated enzymes may deserve attention in the design of new antipsychotic drugs. Despite rather low inhibitory activities against DAAO, AChE and BuChE, it seems that compounds with potent free-radical scavenging properties may play a supporting role in the treatment of neurodegenerative and psychiatric diseases.

## Supplementary Materials

Supplementary materials can be accessed at: http://www.mdpi.com/1420-3049/19/10/15866/s1.

## Supplementary Files

Supplementary File 1
