# Supplementary material for: New Derivatives of 3,4-Dihydroisoquinoline-3-carboxylic Acid with Free-Radical Scavenging, d-Amino Acid Oxidase, Acetylcholinesterase and Butyrylcholinesterase Inhibitory Activity
Source: Molecules. 2014 Sep 30;19(10):15866–90. doi: 10.3390/molecules191015866 (PMC6271642; doi:10.3390/molecules191015866)
Supplement: Supplementary File 1 [file molecules-19-15866-s001.pdf]

Supplementary Materials

Figure S1. ^{1}H- (**a**) and ^{13}C-NMR (**b**) spectra of dibenzyl 2-(2-bromo-5-benzyloxybenzyl)-2-formamidomalonate **3a**.

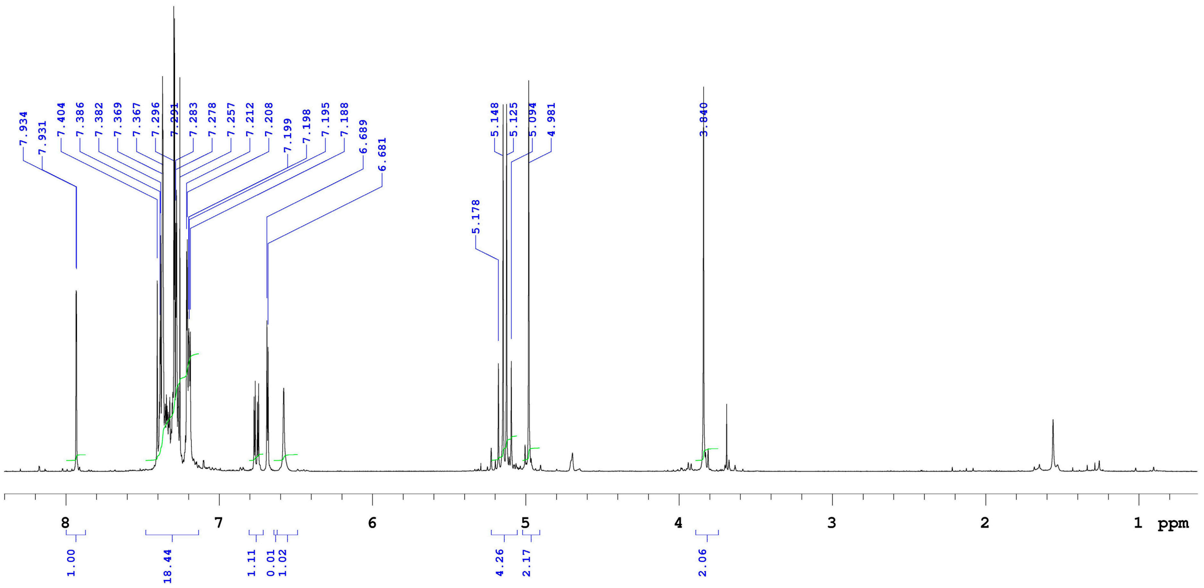

(**a**)

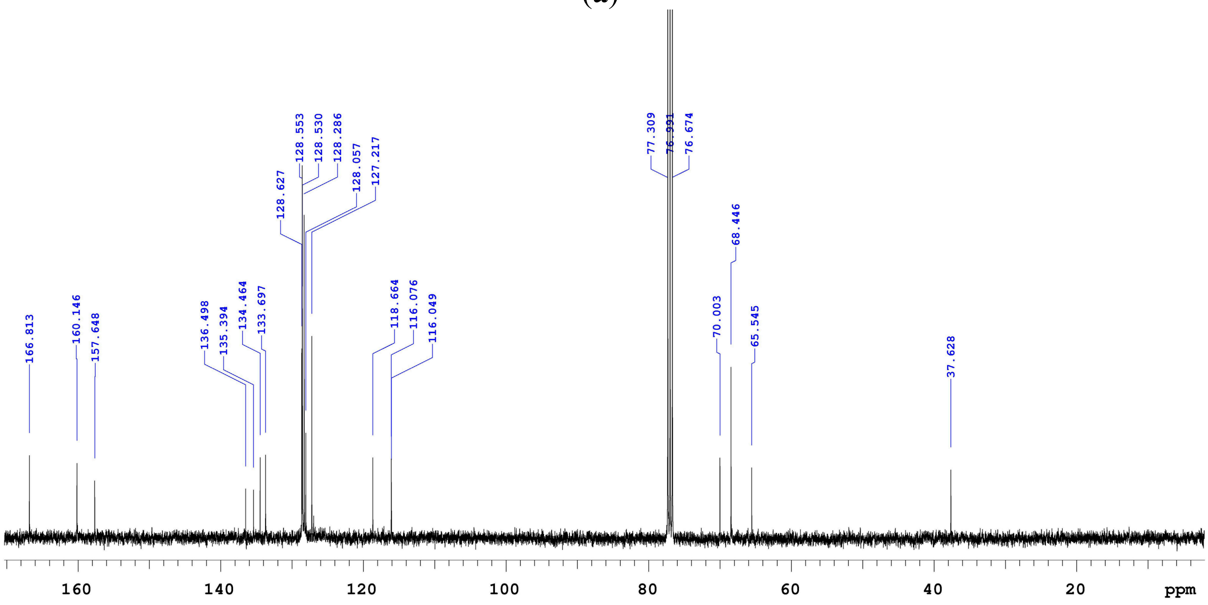

(**b**)

Figure S2. ^{1}H- (**a**) and ^{13}C-NMR (**b**) spectra of dibenzyl 2-(3-benzyloxy-4-bromobenzyl)-2-formamidomalonate **3b**.

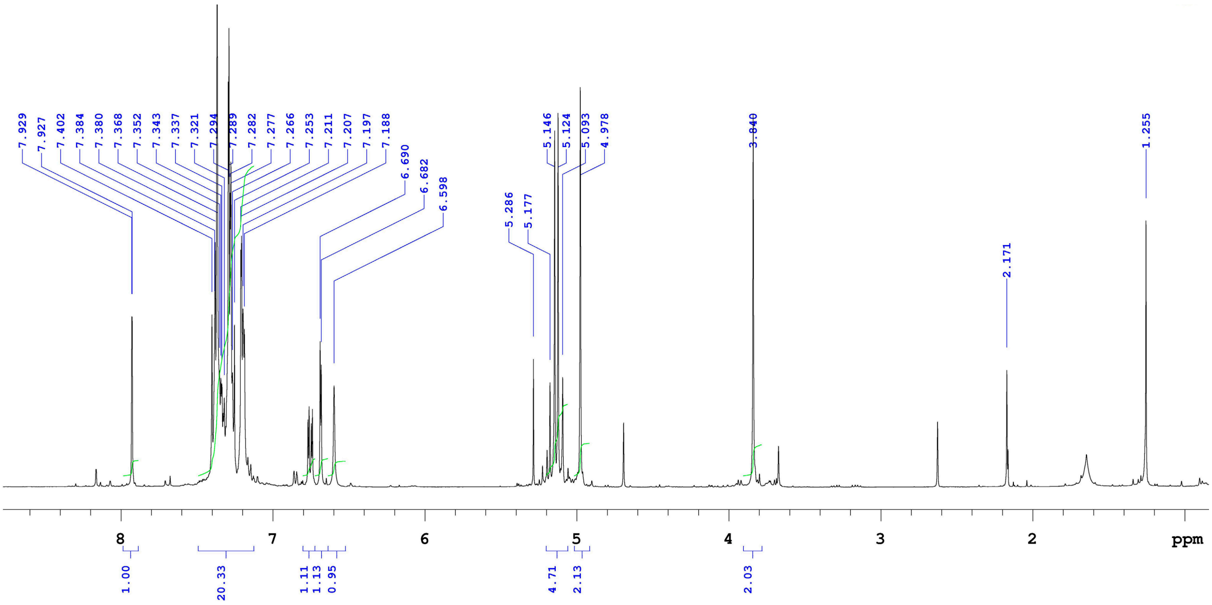

(**a**)

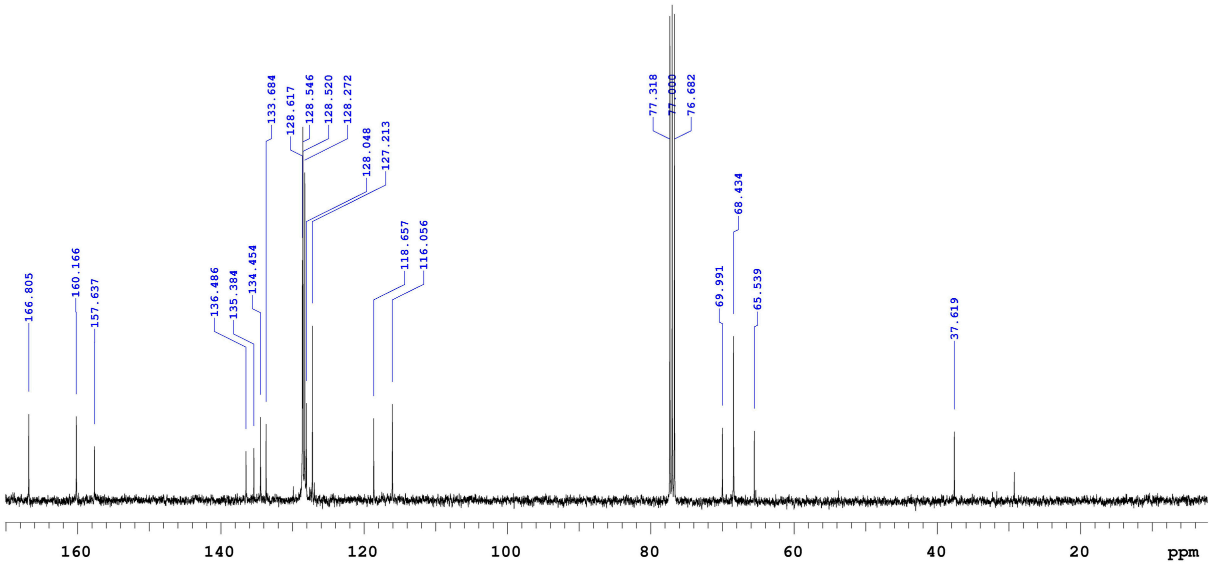

(**b**)

Figure S3. ^{1}H- (**a**) and ^{13}C-NMR (**b**) spectra of dibenzyl (3,5-dibenzyloxybenzyl)-2-formamidomalonate **3c**.

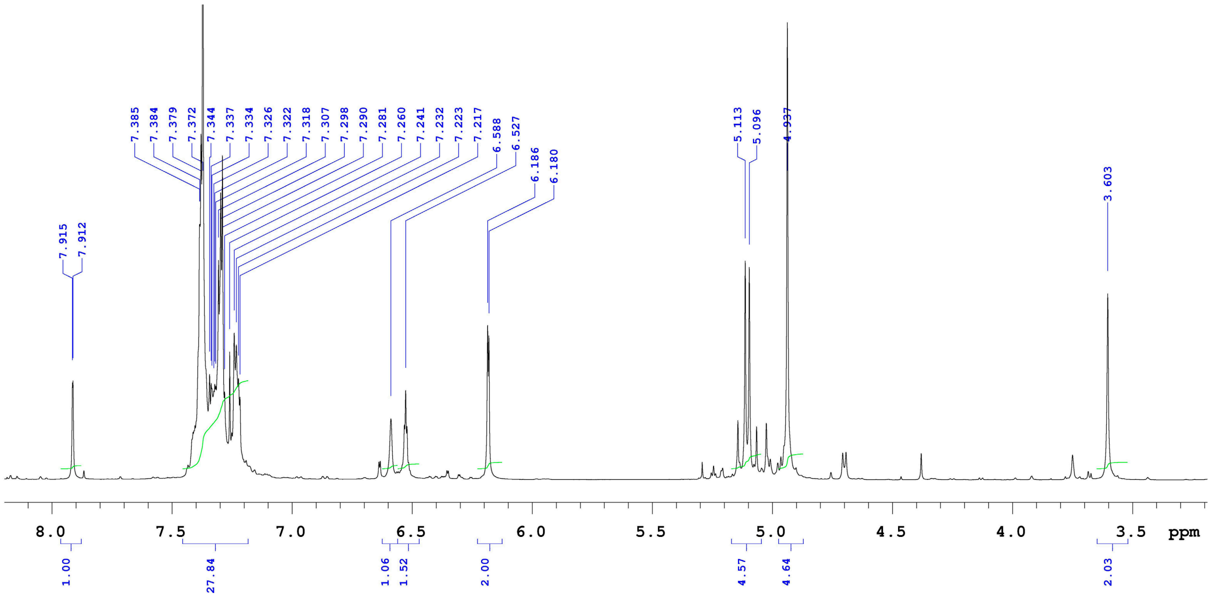

(**a**)

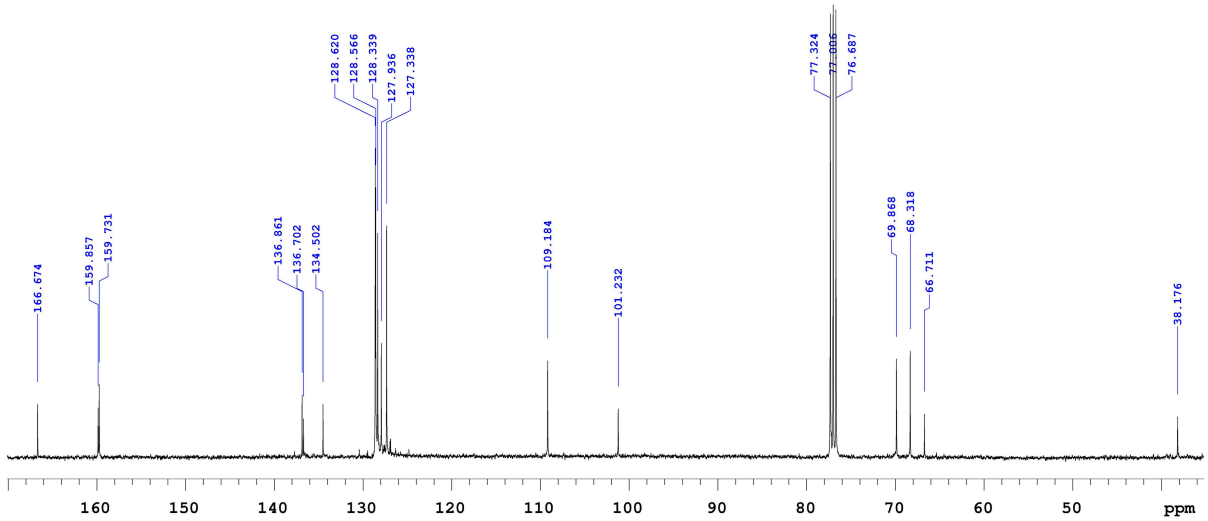

(**b**)

Figure S4. ^{1}H- (**a**) and ^{13}C-NMR (**b**) spectra of dibenzyl 2-(3-iodo-4-benzyloxy-5-methoxybenzyl)-2-formamidomalonate **3d**.

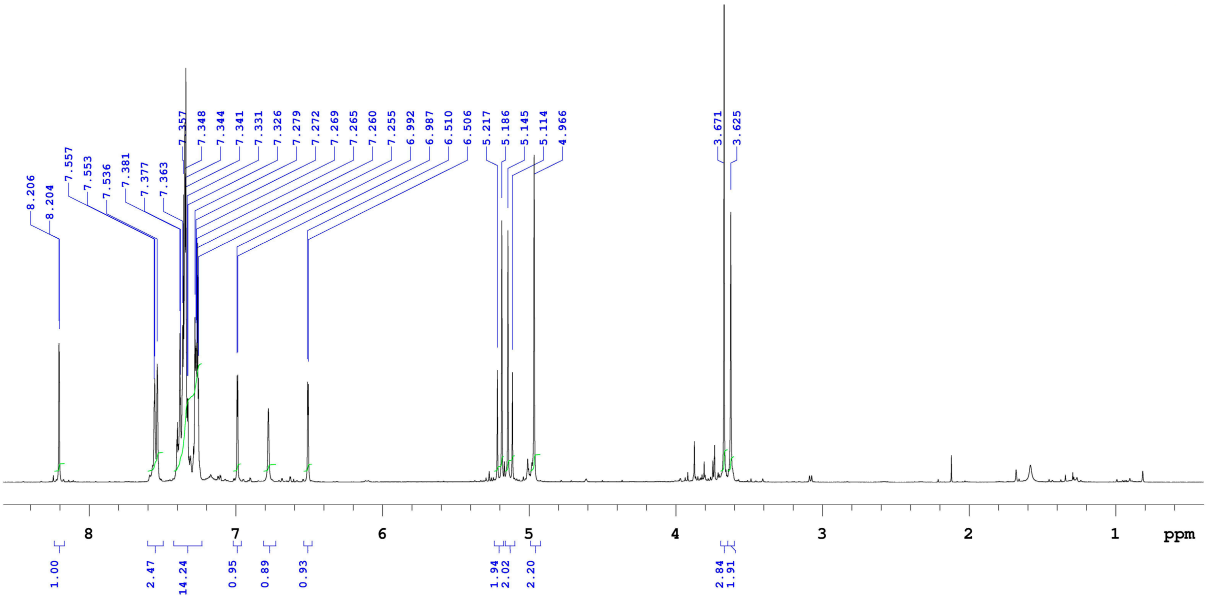

(**a**)

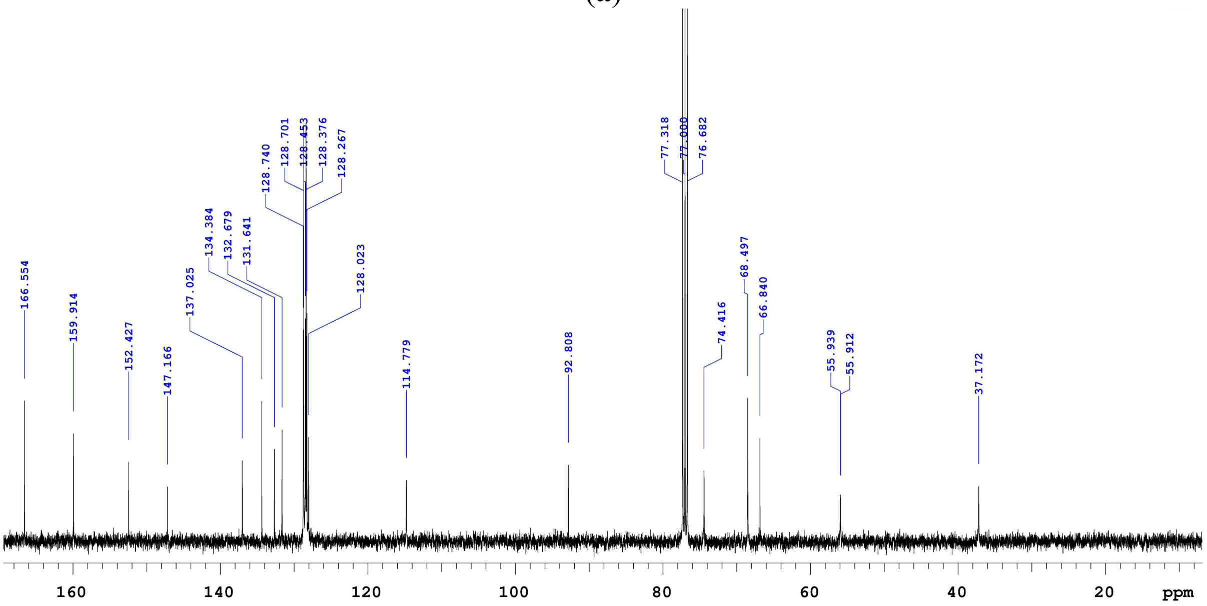

(**b**)

Figure S5. ^{1}H- (**a**) and ^{13}C-NMR (**b**) spectra of dibenzyl 2-(5-bromo-2,3-dimethoxybenzyl)-2-formamidomalonate **3f**.

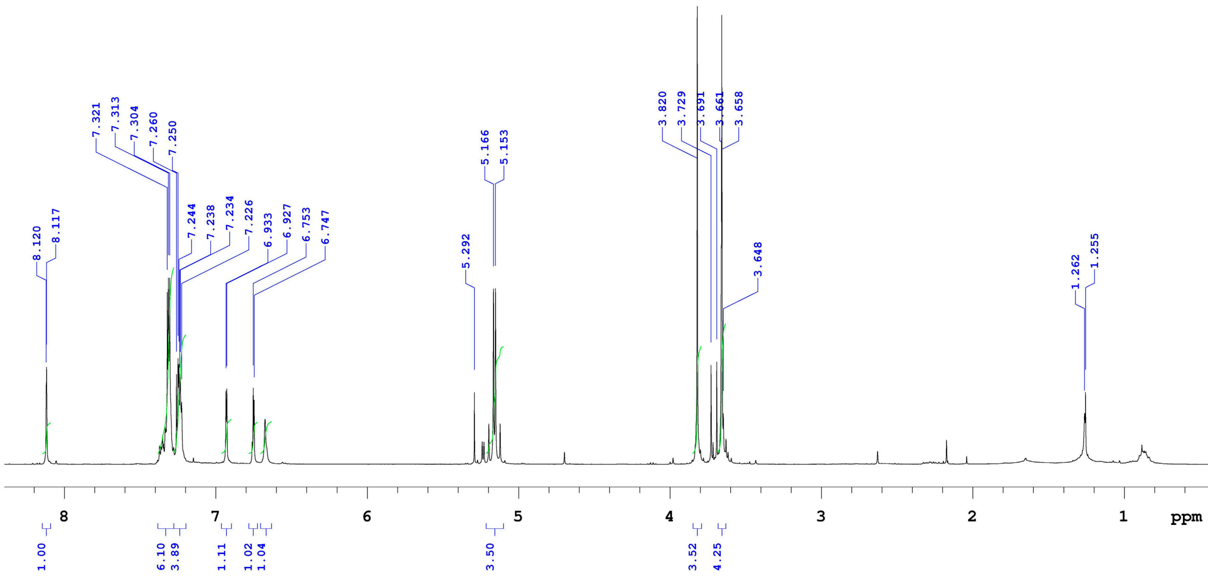

(**a**)

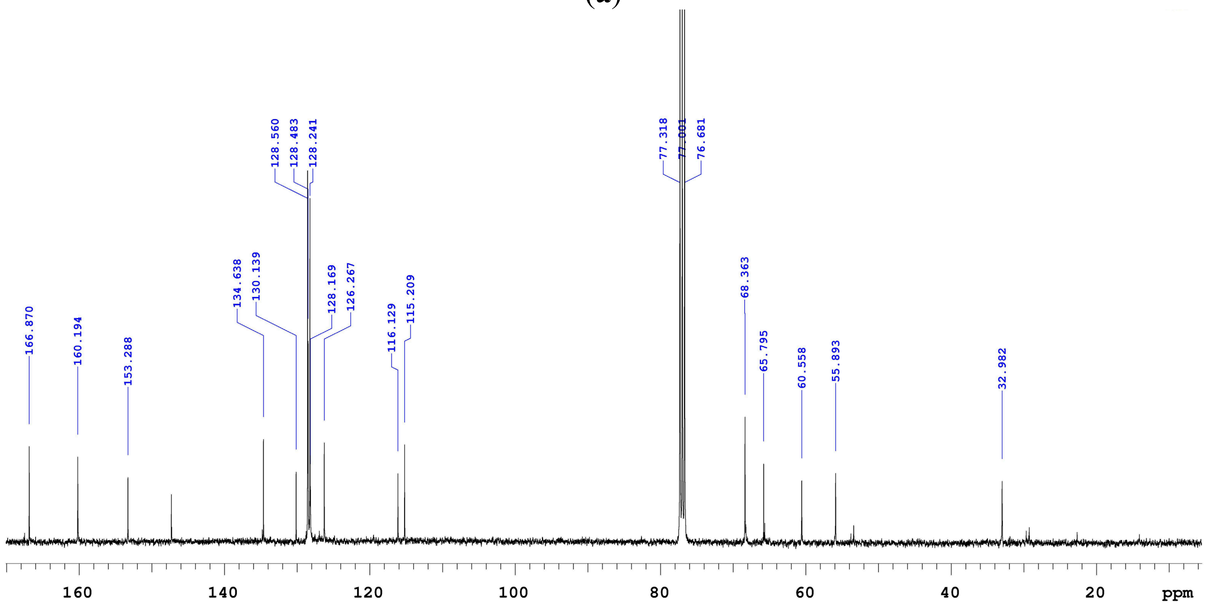

(**b**)

Figure S6. ^{1}H- (**a**) and ^{13}C-NMR (**b**) spectra of dibenzyl 2-(3-bromo-4,5-dimethoxybenzyl)-2-formamidomalonate **3g**.

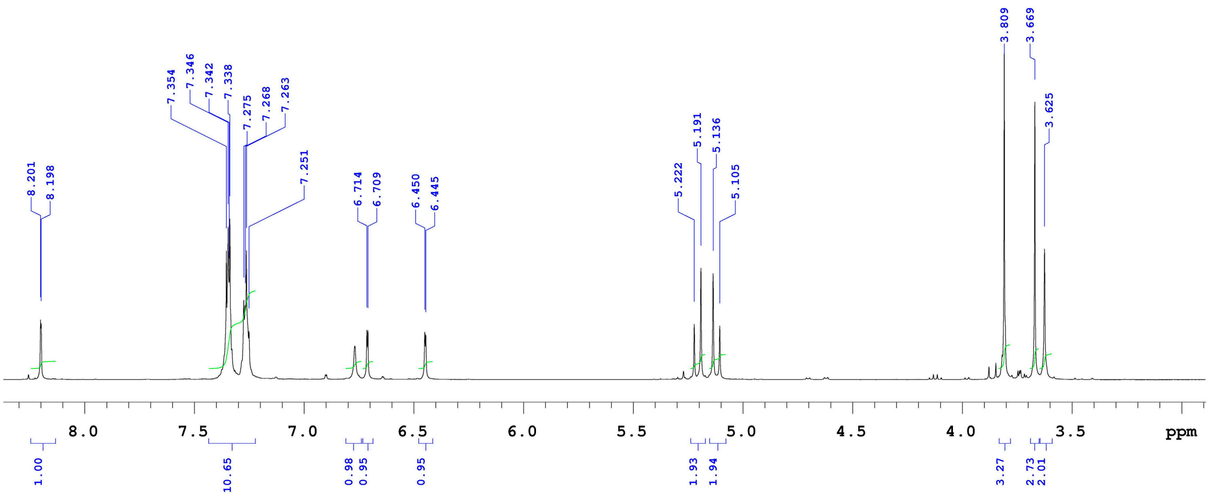

(**a**)

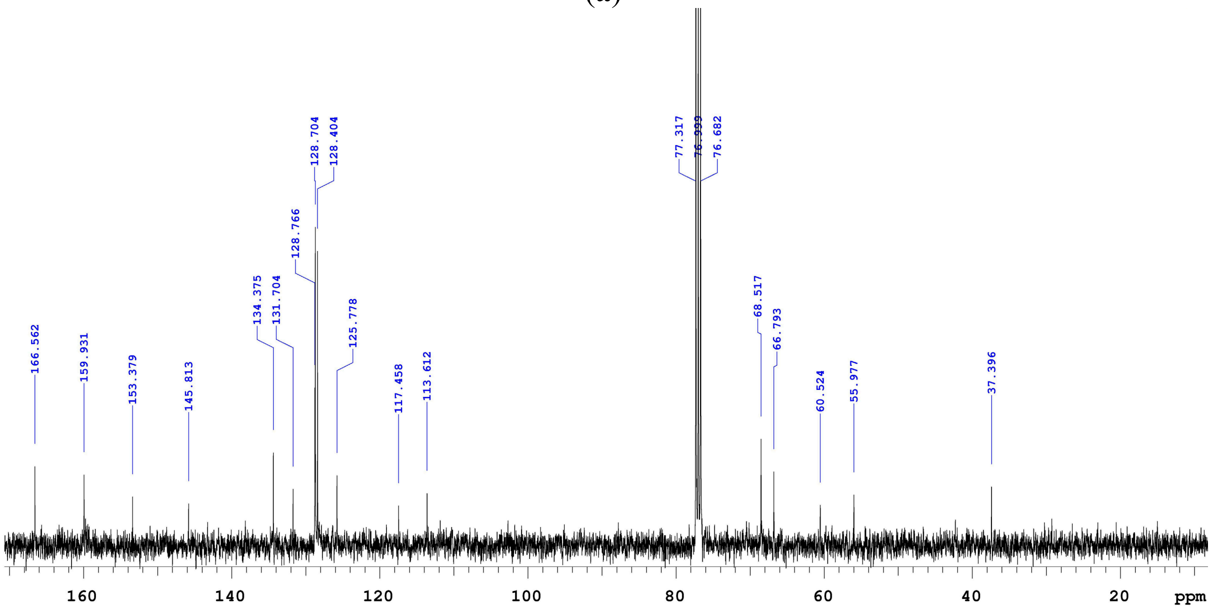

(**b**)

Figure S7. ^{1}H- (**a**) and ^{13}C-NMR (**b**) spectra of dibenzyl 2-(3-chloro-4,5-dimethoxybenzyl)-2-formamidomalonate **3h**.

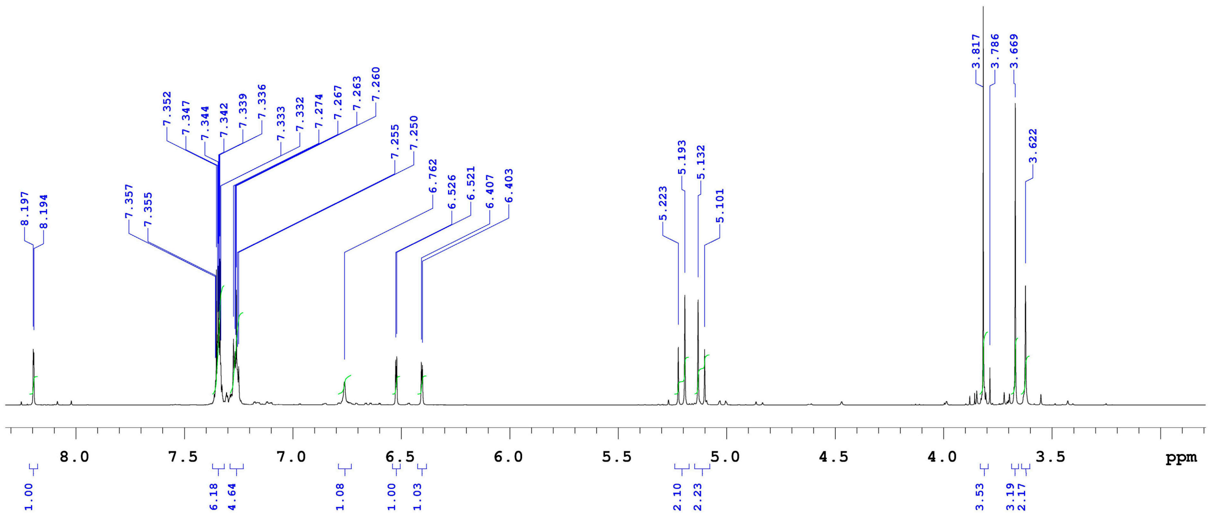

(**a**)

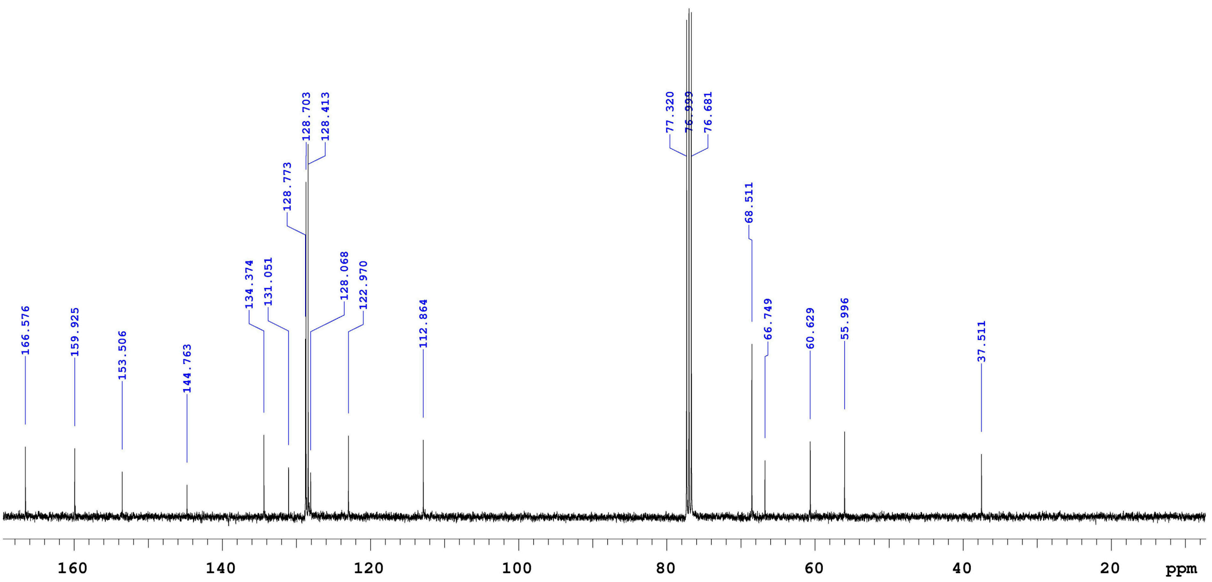

(**b**)

Figure S8. ^{1}H- (**a**) and ^{13}C-NMR (**b**) spectra of dibenzyl 2-(3,4,5-trimethoxybenzyl)-2-formamidomalonate **3i**.

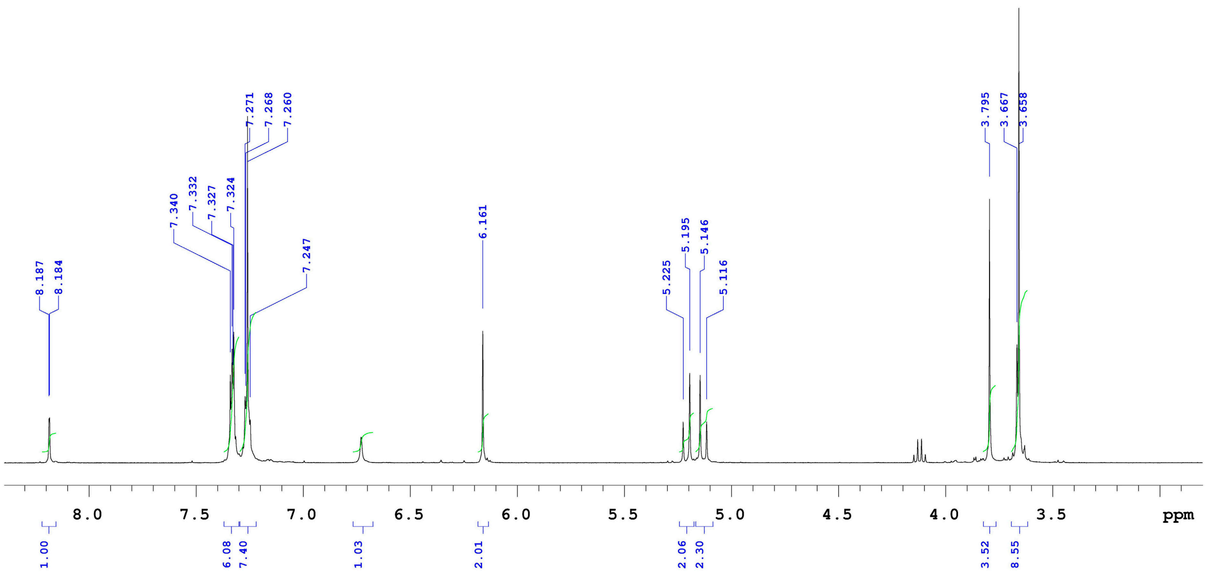

(**a**)

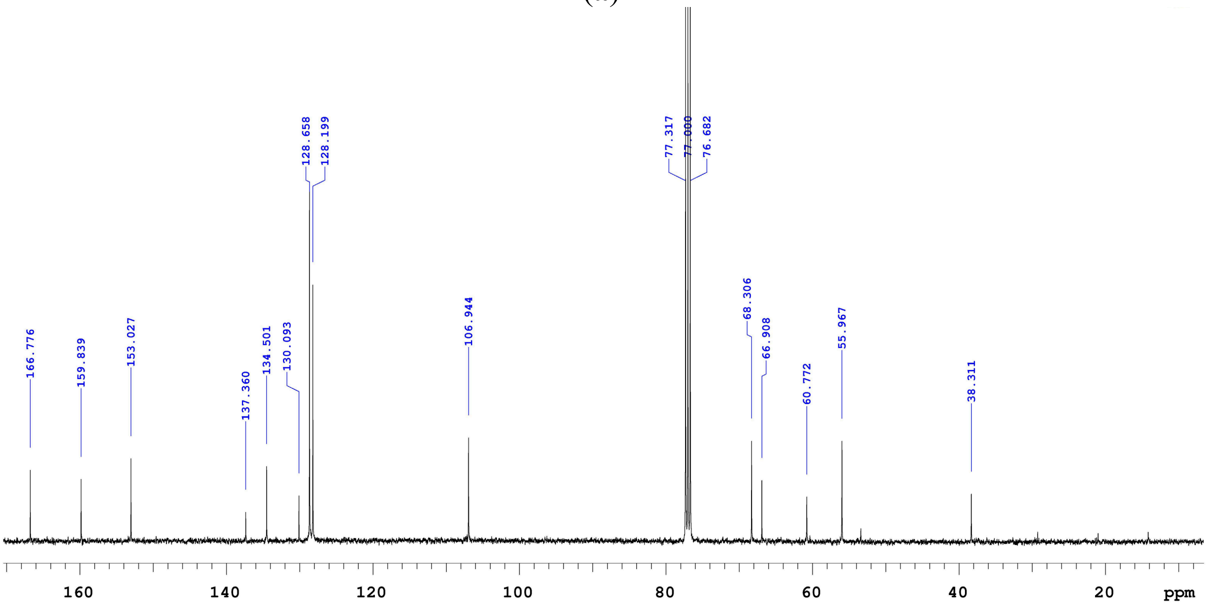

(**b**)

Figure S9. ^{1}H- (**a**) and ^{13}C-NMR (**b**) spectra of dibenzyl 2-(4-bromo-3,5-dibenzyloxybenzyl)-2-formamidomalonate **3j**.

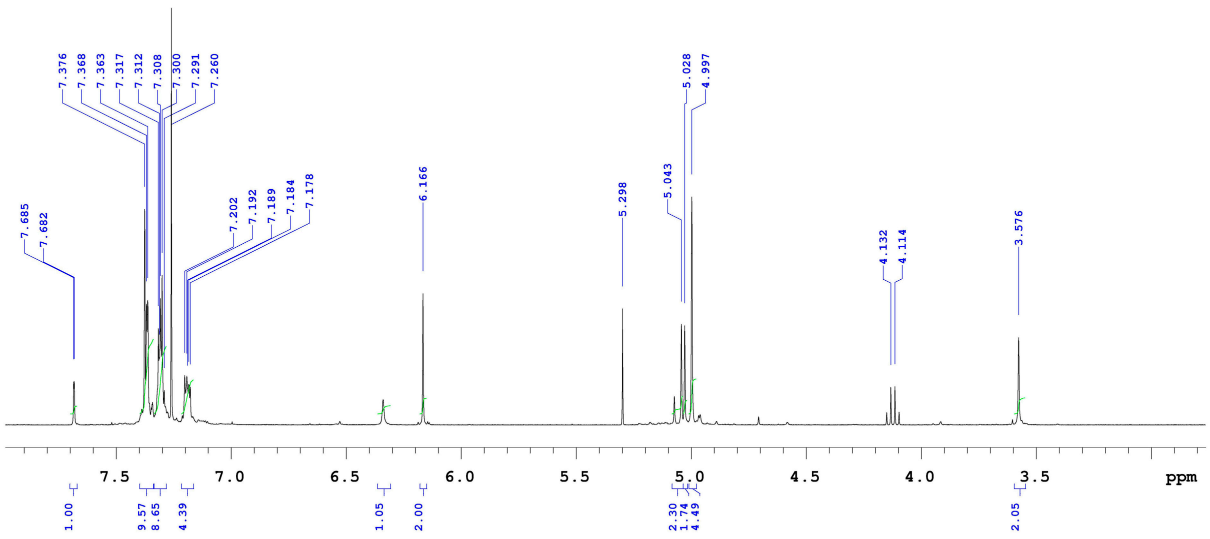

(**a**)

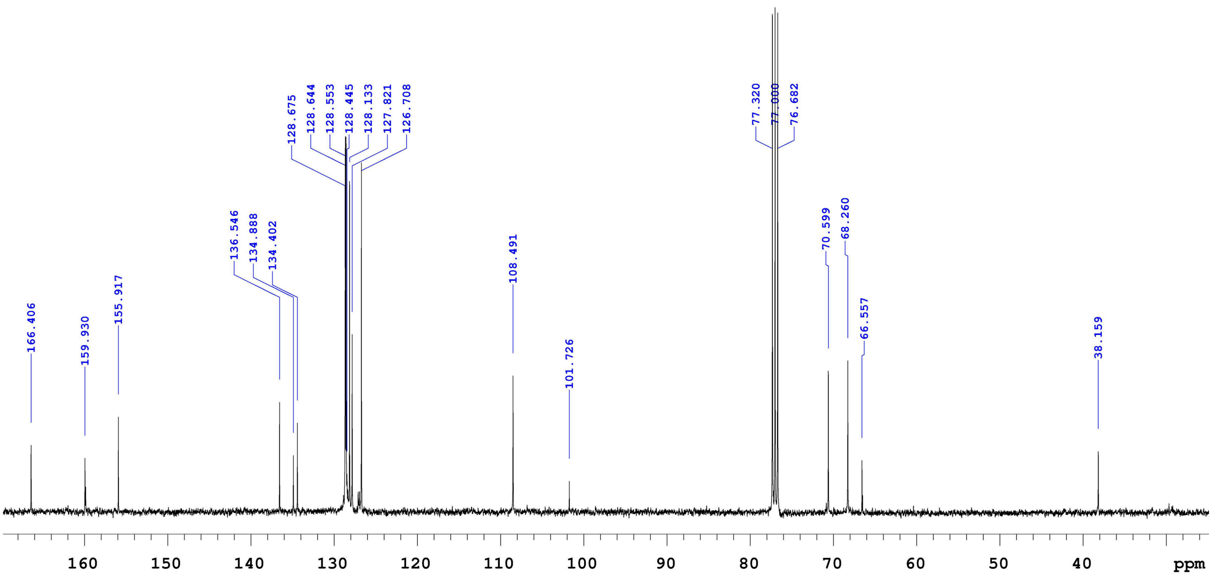

(**b**)

Figure S10. ^{1}H- (**a**) and ^{13}C-NMR (**b**) spectra of dibenzyl (2-chloro-3,5-dibenzyloxybenzyl)-2-formamidomalonate **3k**.

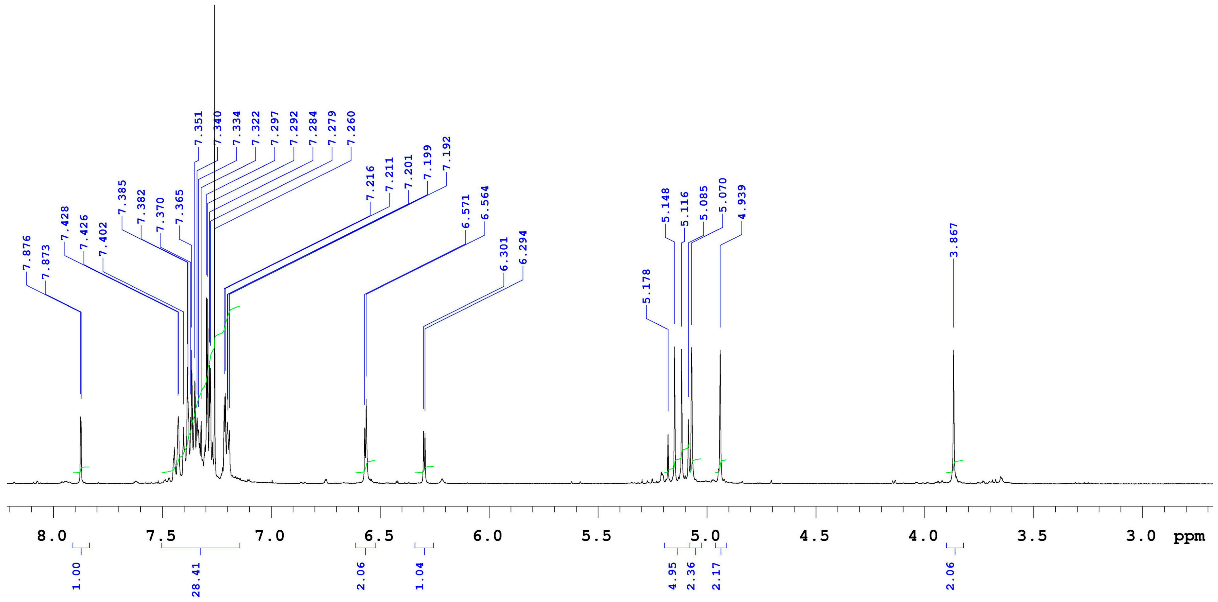

(**a**)

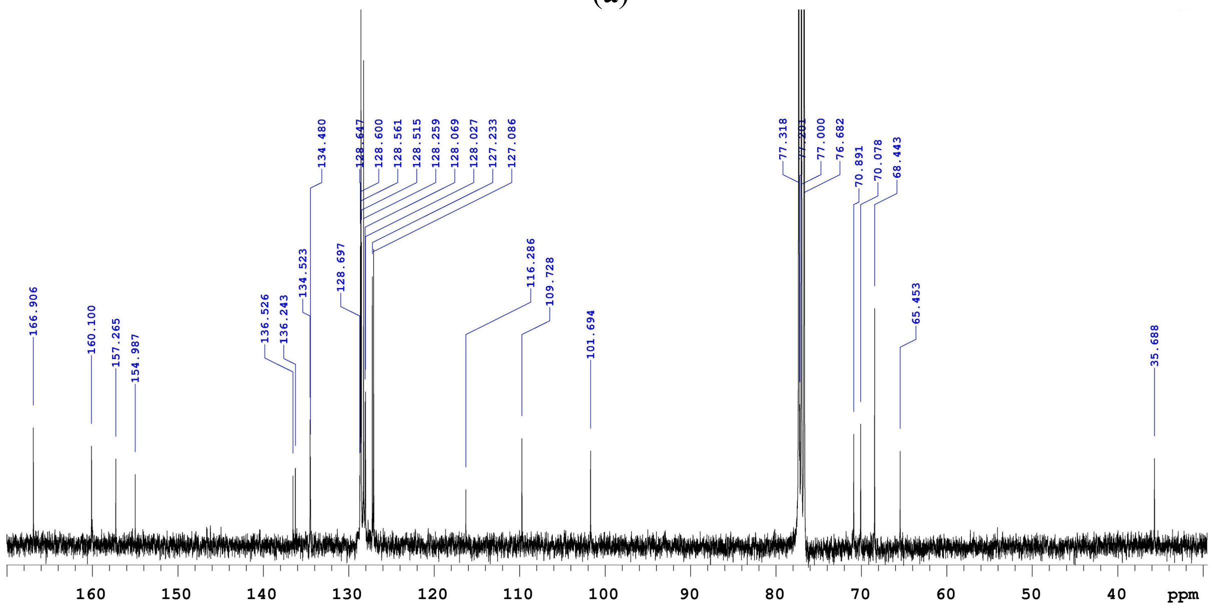

(**b**)

Figure S11. ^{1}H- (**a**) and ^{13}C-NMR (**b**) spectra of dibenzyl 2-(2-chloro-3,4-dimethoxybenzyl)-2-formamidomalonate **3l**.

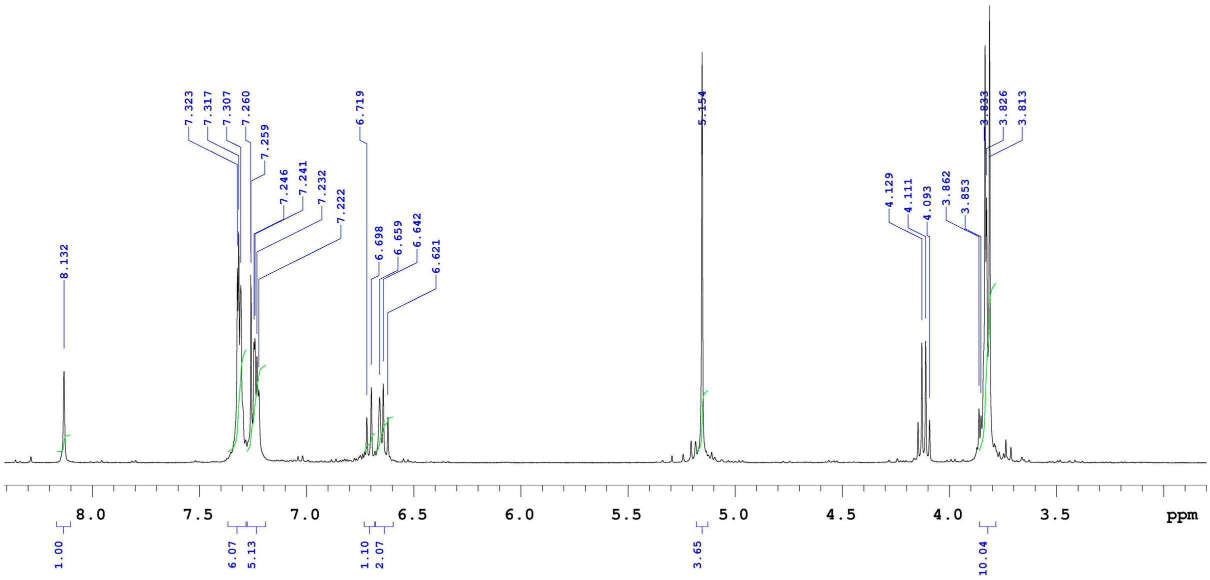

(**a**)

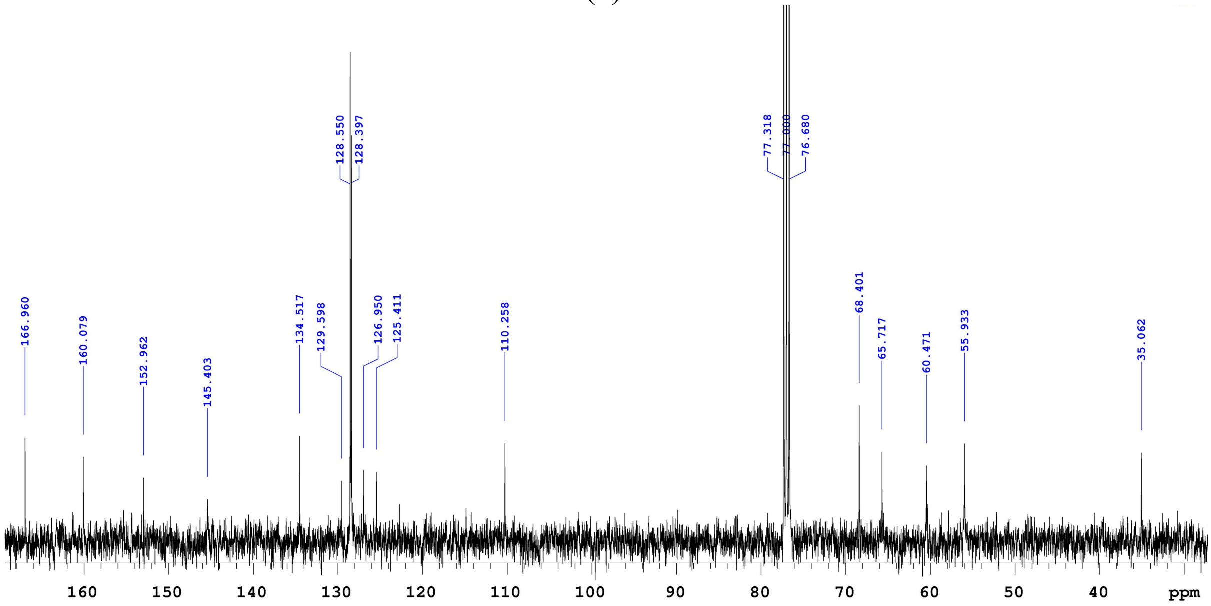

(**b**)

Figure S12. ^{1}H- (**a**) and ^{13}C-NMR (**b**) spectra of dibenzyl 5-bromo-8-benzyloxy-3,4-dihydroisoquinolin-3,3-dicarboxylate **4a**.

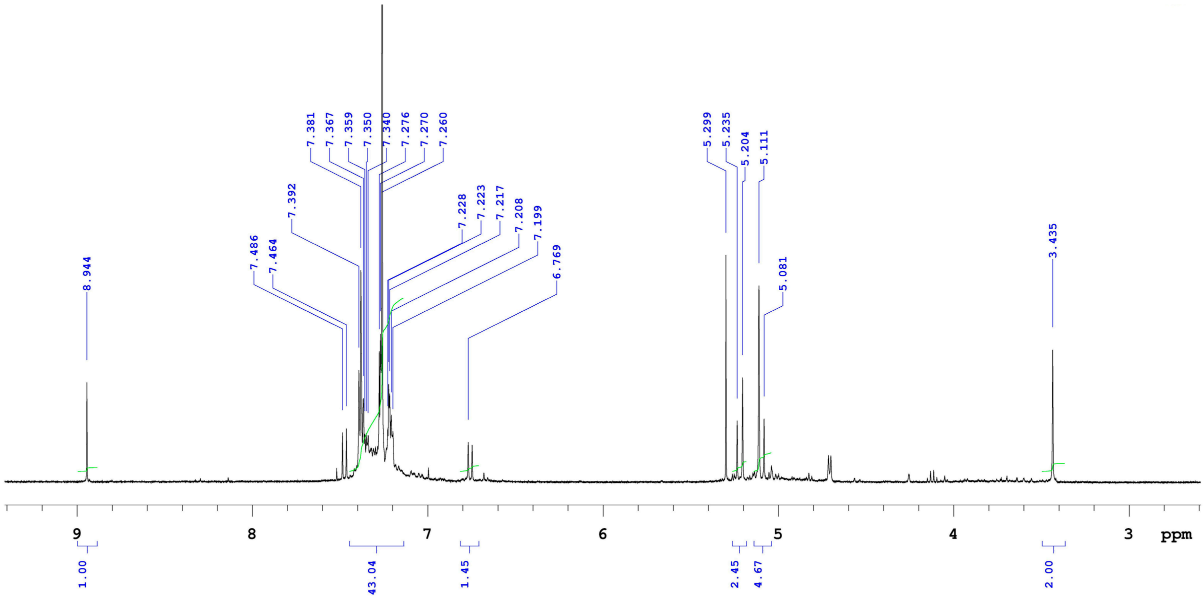

(**a**)

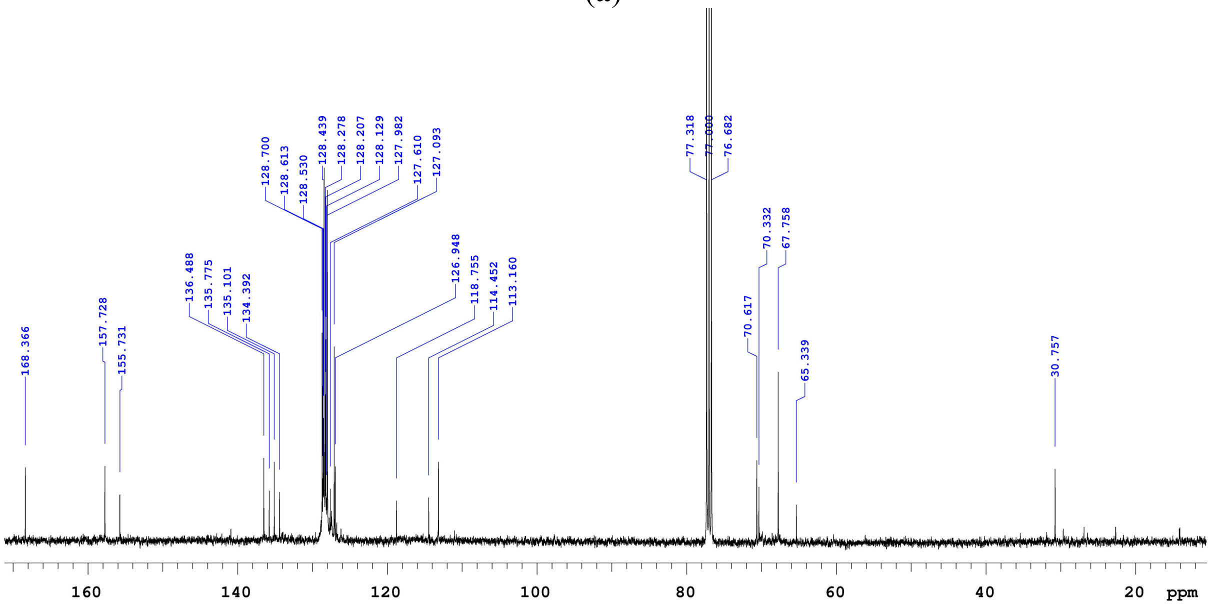

(**b**)

Figure S13. ^{1}H- (**a**) and ^{13}C-NMR (**b**) spectra of dibenzyl 8-benzyloxy-7-bromo-3,4-dihydroisoquinolin-3,3-dicarboxylate **4b**.

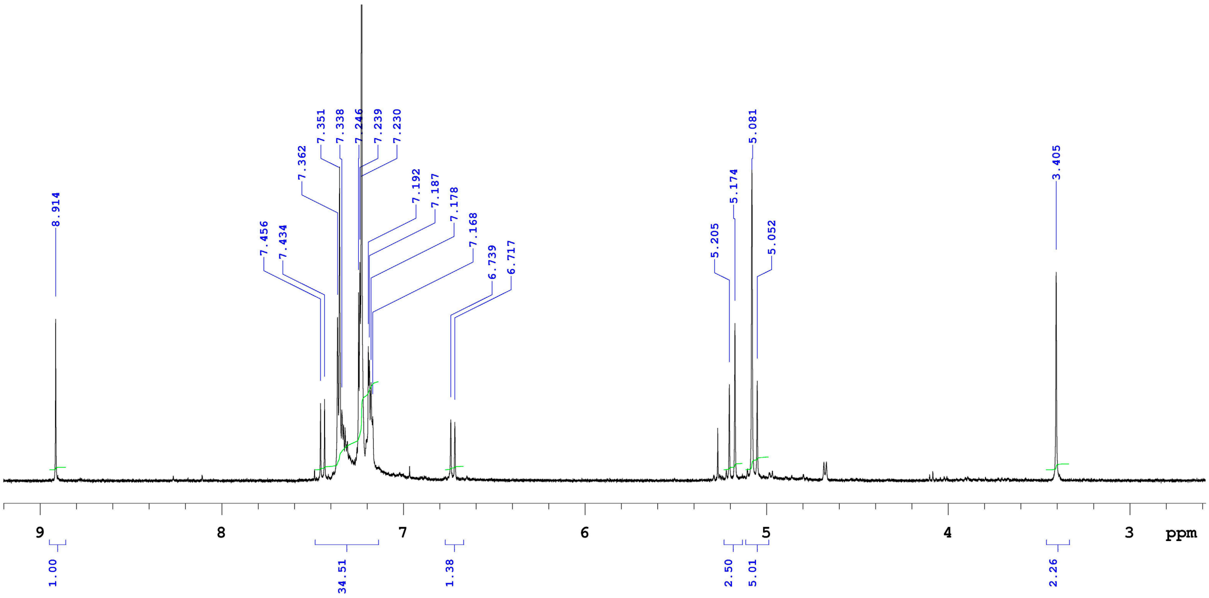

(**a**)

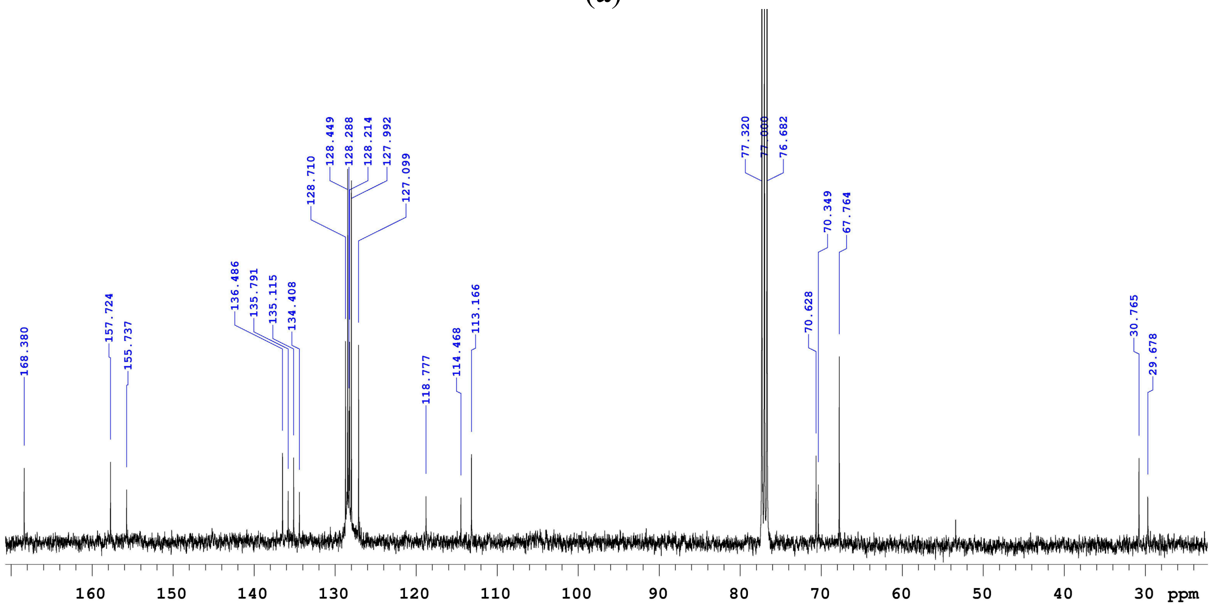

(**b**)

Figure S14. ^{1}H- (**a**) and ^{13}C-NMR (**b**) spectra of dibenzyl 6,8-dibenzyloxy-3,4-dihydroisoquinolin-3,3-dicarboxylate **4c**.

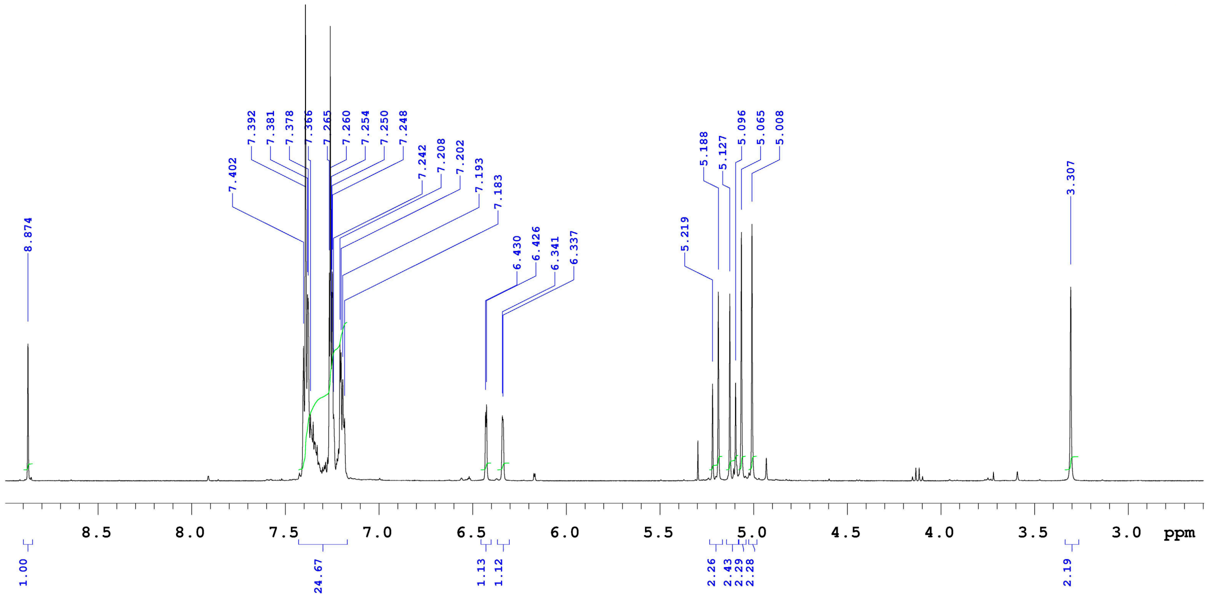

(**a**)

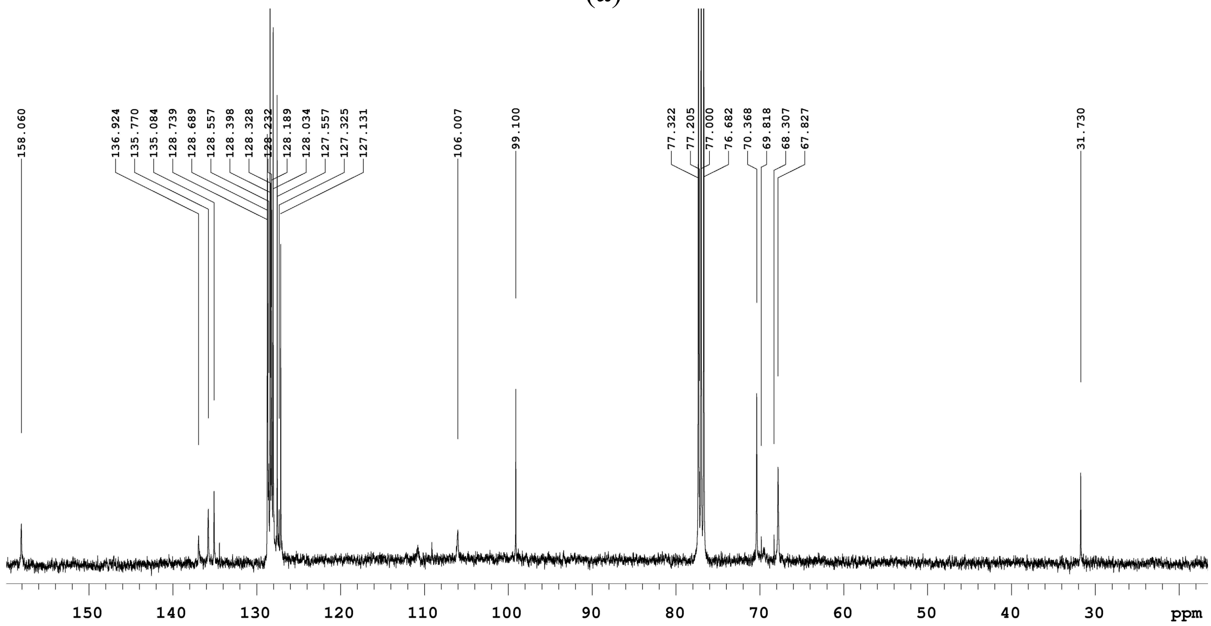

(**b**)

Figure S15. ^{1}H- (**a**) and ^{13}C-NMR (**b**) spectra of dibenzyl 6-methoxy-7-benzyloxy-8-iodo-3,4-dihydroisoquinolin-3,3-dicarboxylate **4d**.

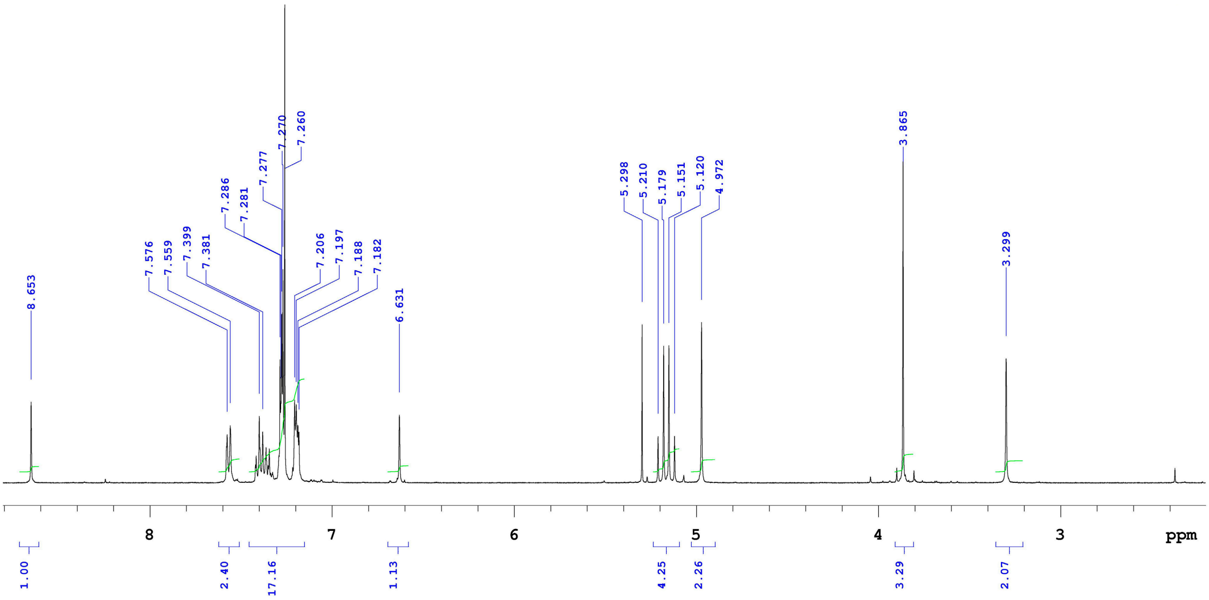

(**a**)

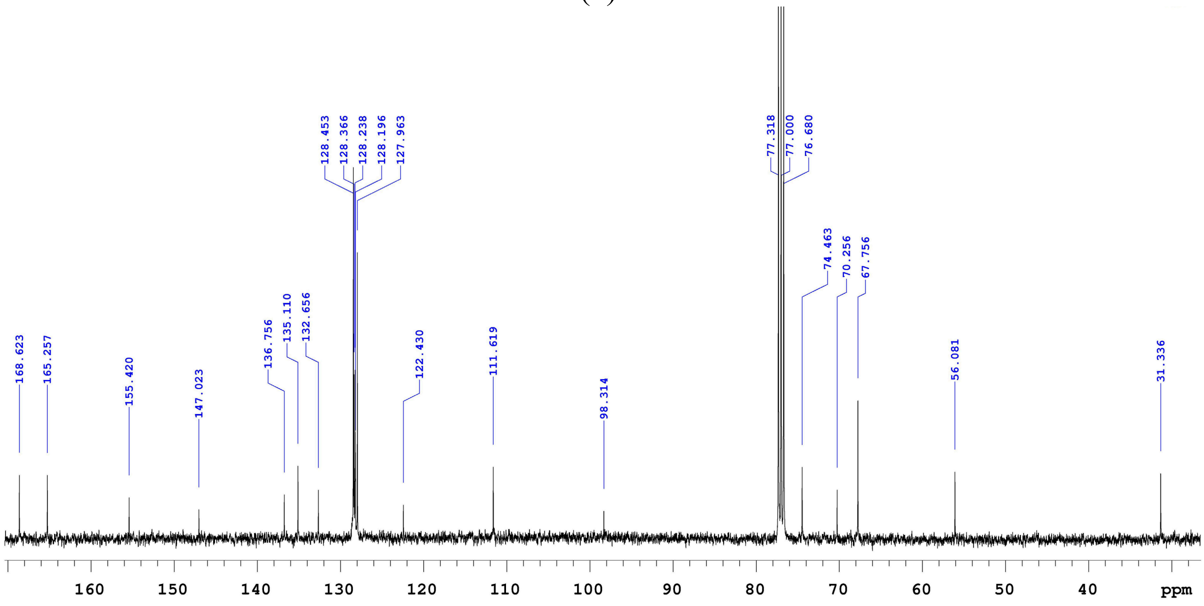

(**b**)

Figure S16. ^{1}H- (**a**) and ^{13}C-NMR (**b**) spectra of dibenzyl 8-bromo-5,6-dimethoxy-3,4-dihydroisoquinolin-3,3-dicarboxylate **4f**.

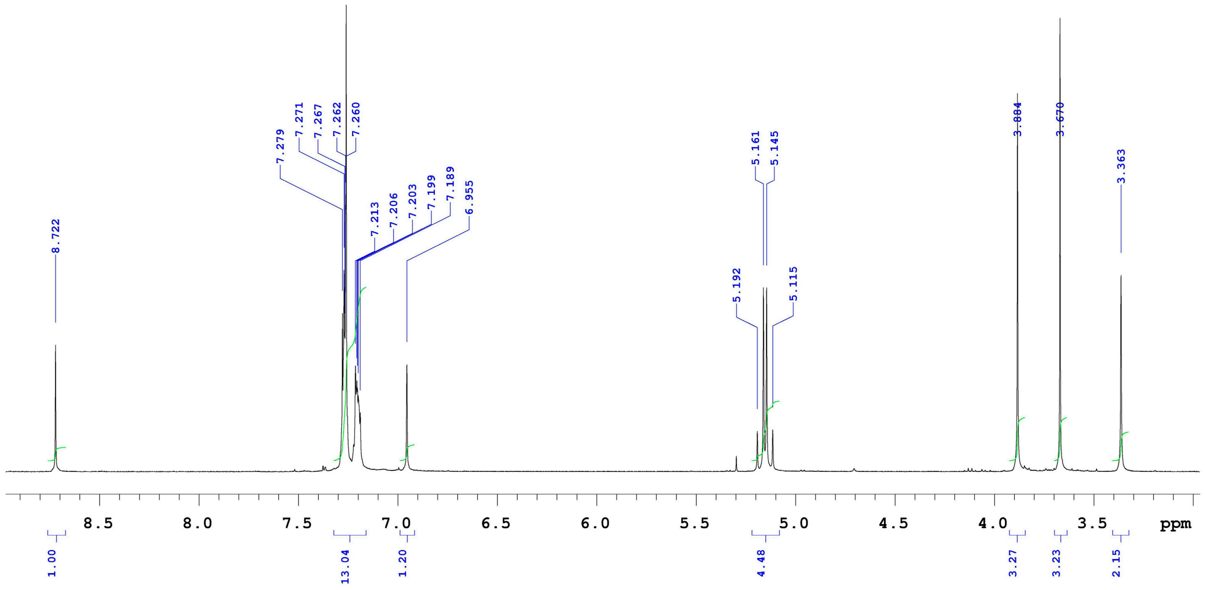

(**a**)

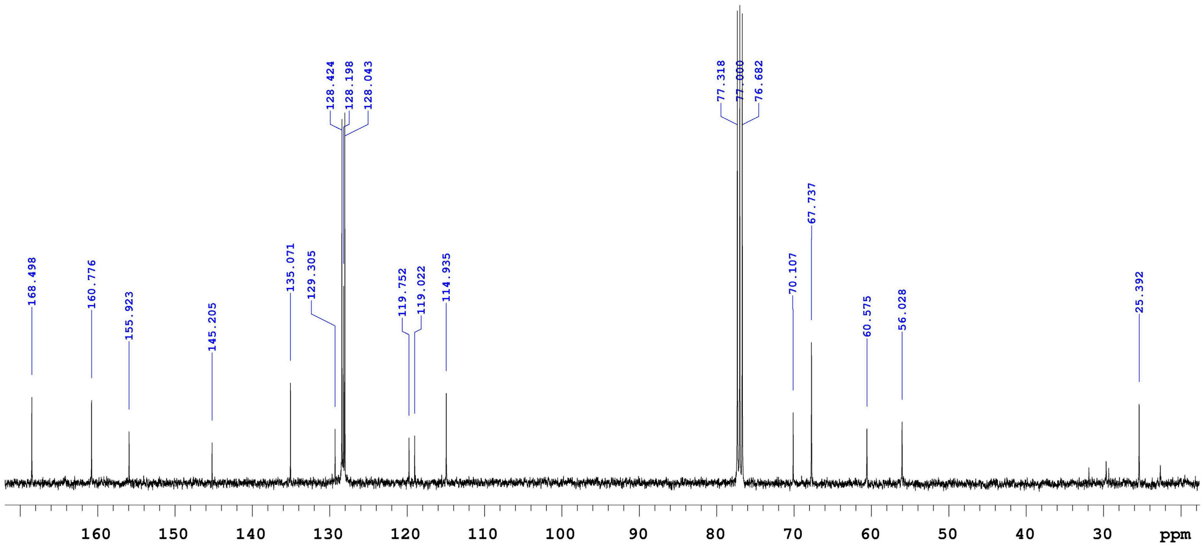

(**b**)

Figure S17. ^{1}H- (**a**) and ^{13}C-NMR (**b**) spectra of dibenzyl 8-bromo-6,7-dimethoxy-3,4-dihydroisoquinolin-3,3-dicarboxylate **4g**.

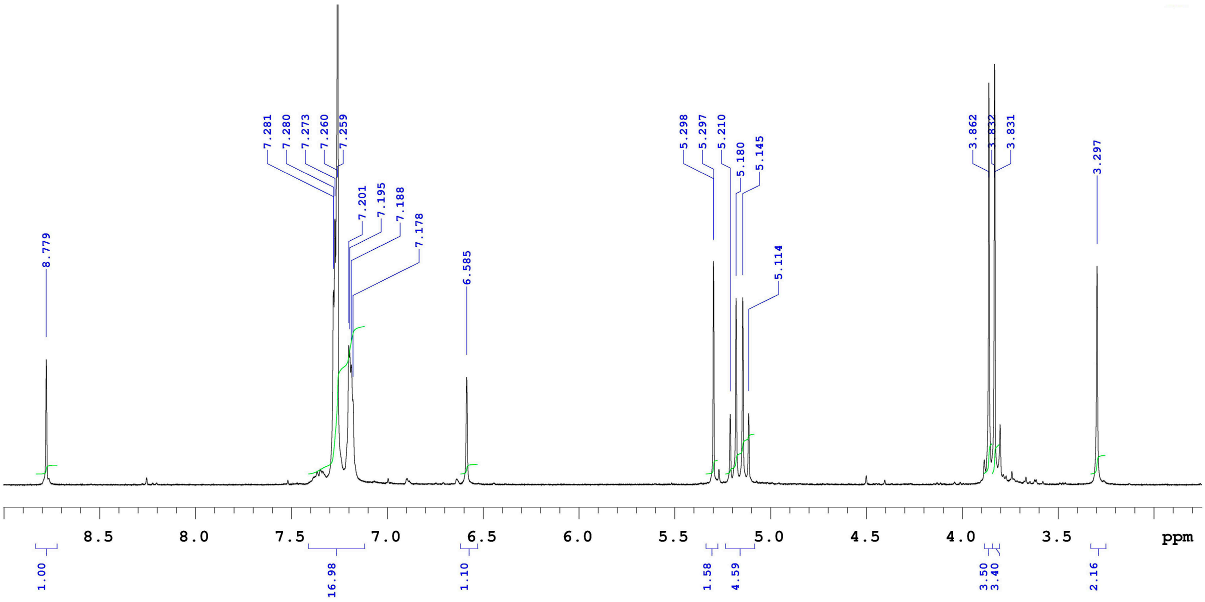

(**a**)

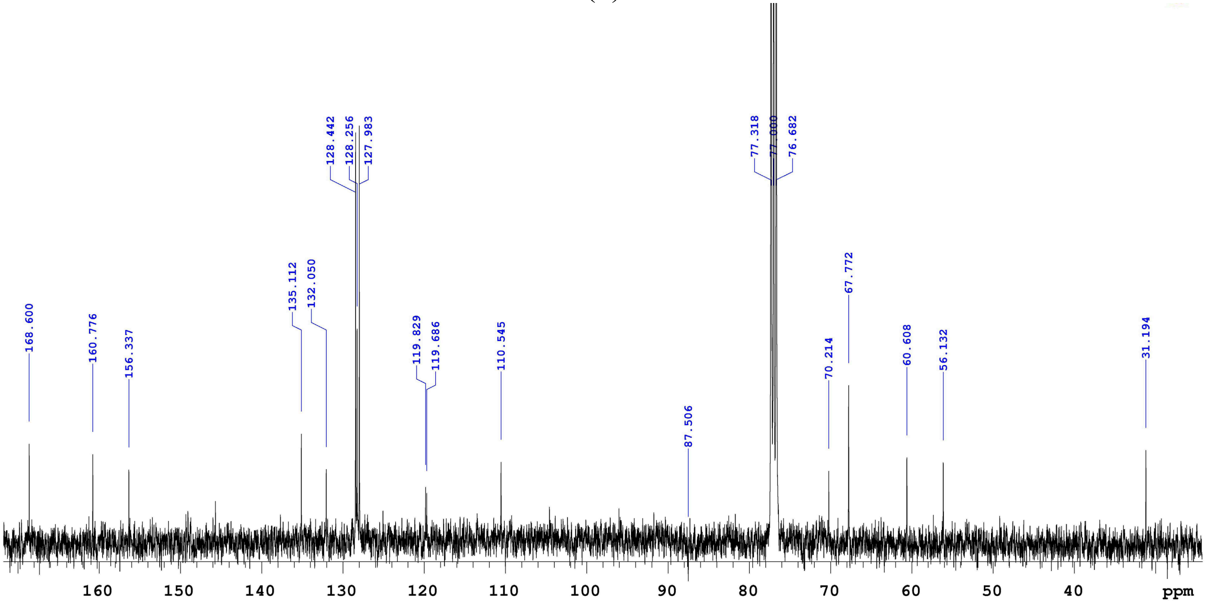

(**b**)

Figure S18. ^{1}H- (**a**) and ^{13}C-NMR (**b**) spectra of dibenzyl 8-chloro-6,7-dimethoxy-3,4-dihydroisoquinolin-3,3-dicarboxylate **4h**.

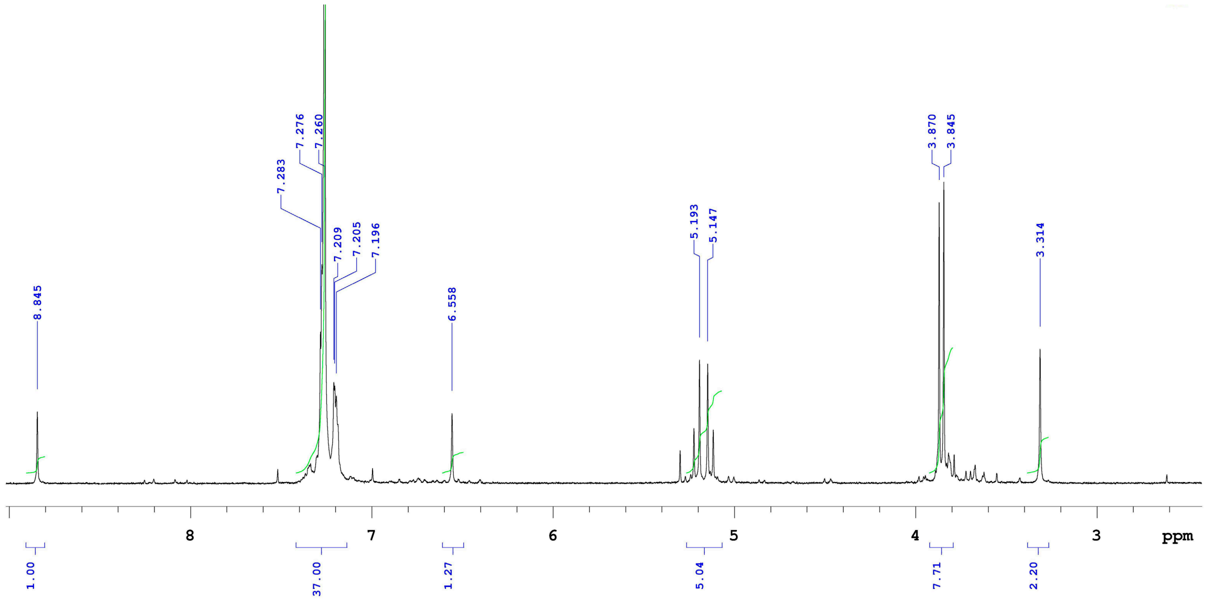

(**a**)

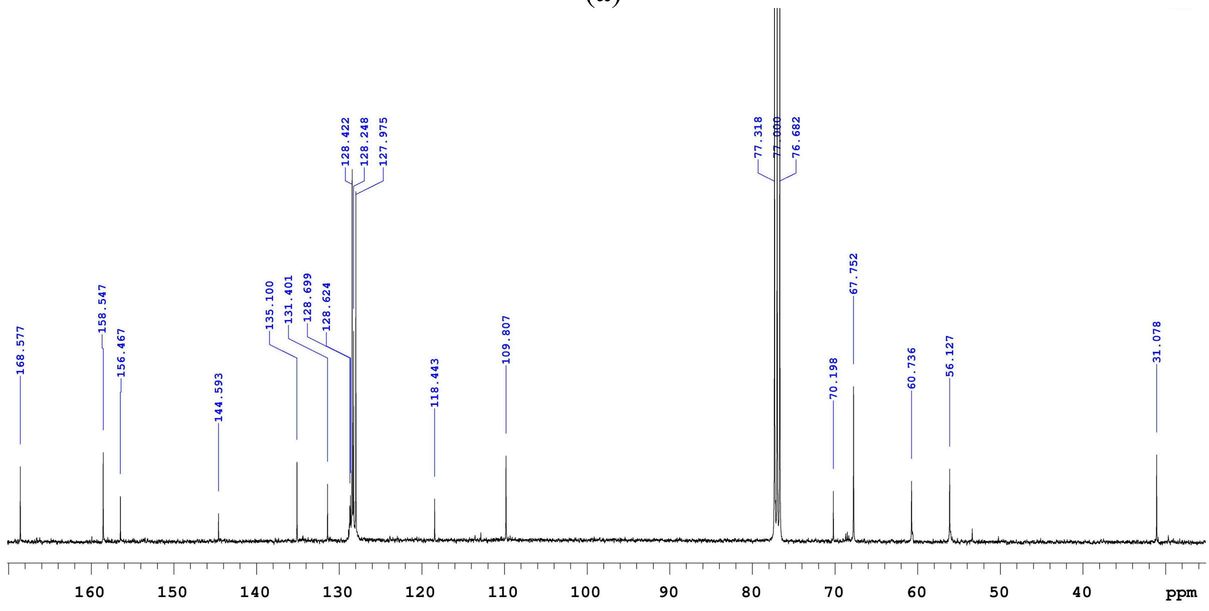

(**b**)

Figure S19. ^{1}H- (**a**) and ^{13}C-NMR (**b**) spectra of dibenzyl 6,7,8-trimethoxy-3,4-dihydroisoquinolin-3,3-dicarboxylate **4i**.

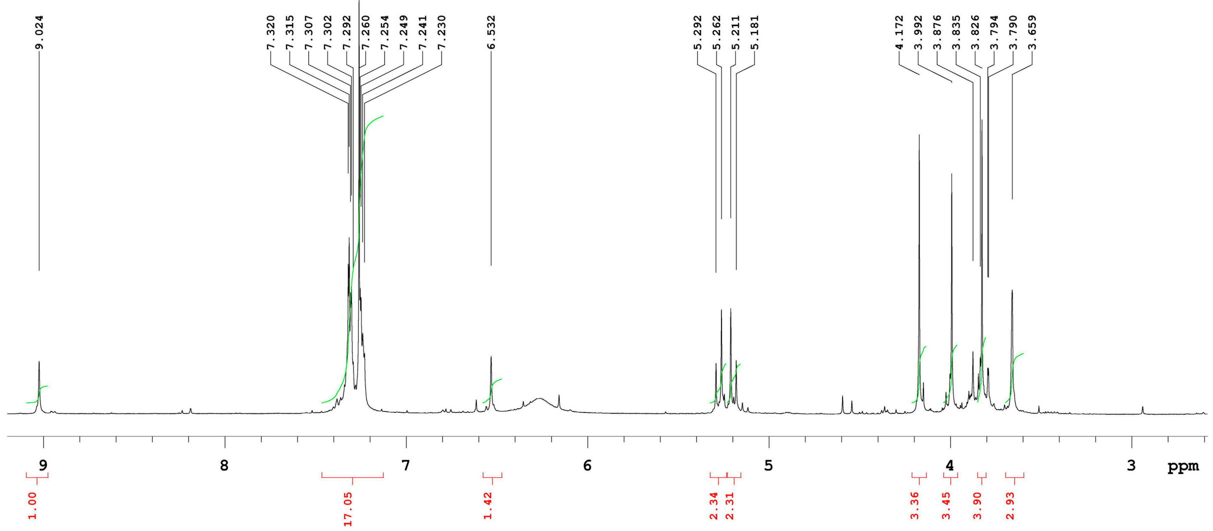

(**a**)

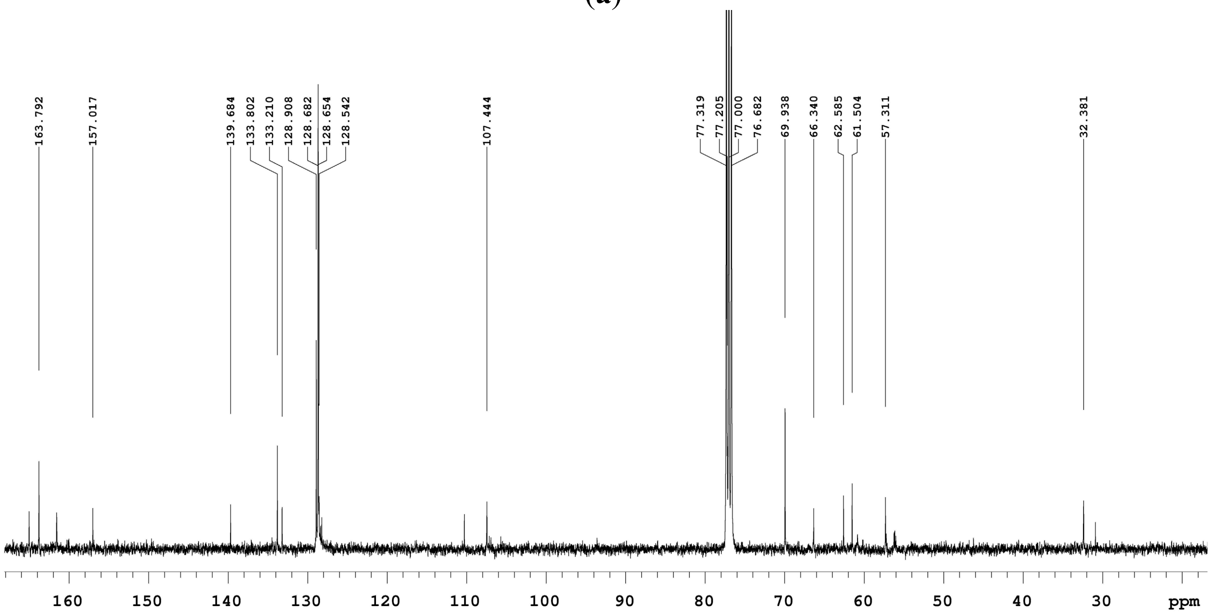

(**b**)

Figure S20. ^{1}H- (**a**) and ^{13}C-NMR (**b**) spectra of dibenzyl 7-bromo-6,8-dibenzyloxy-3,4-dihydroisoquinolin-3,3-dicarboxylate **4j**.

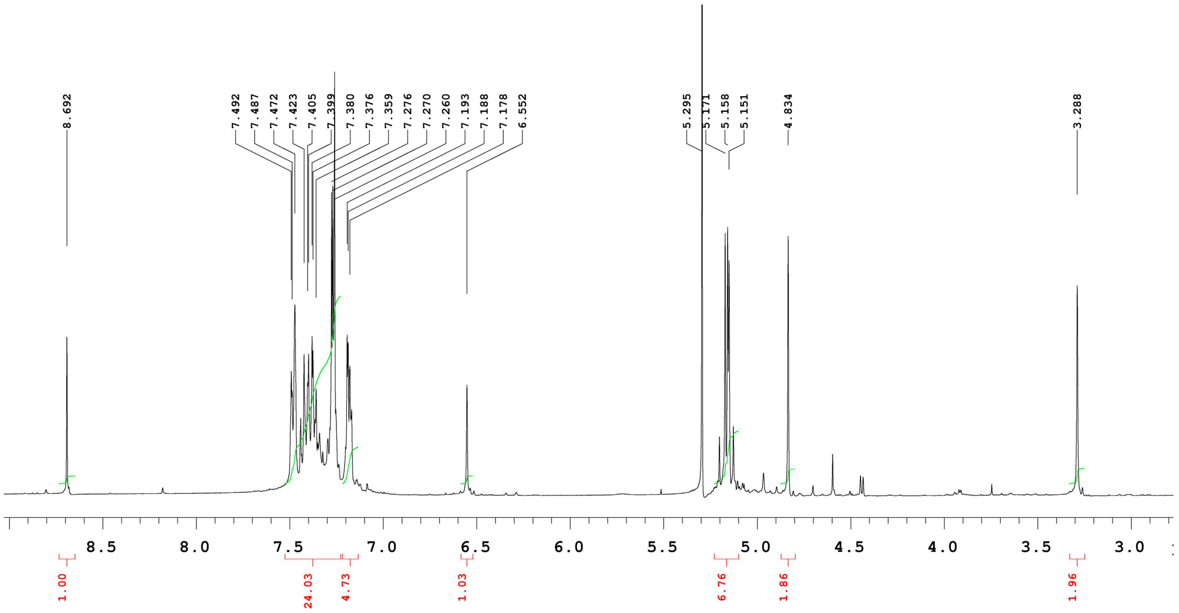

(**a**)

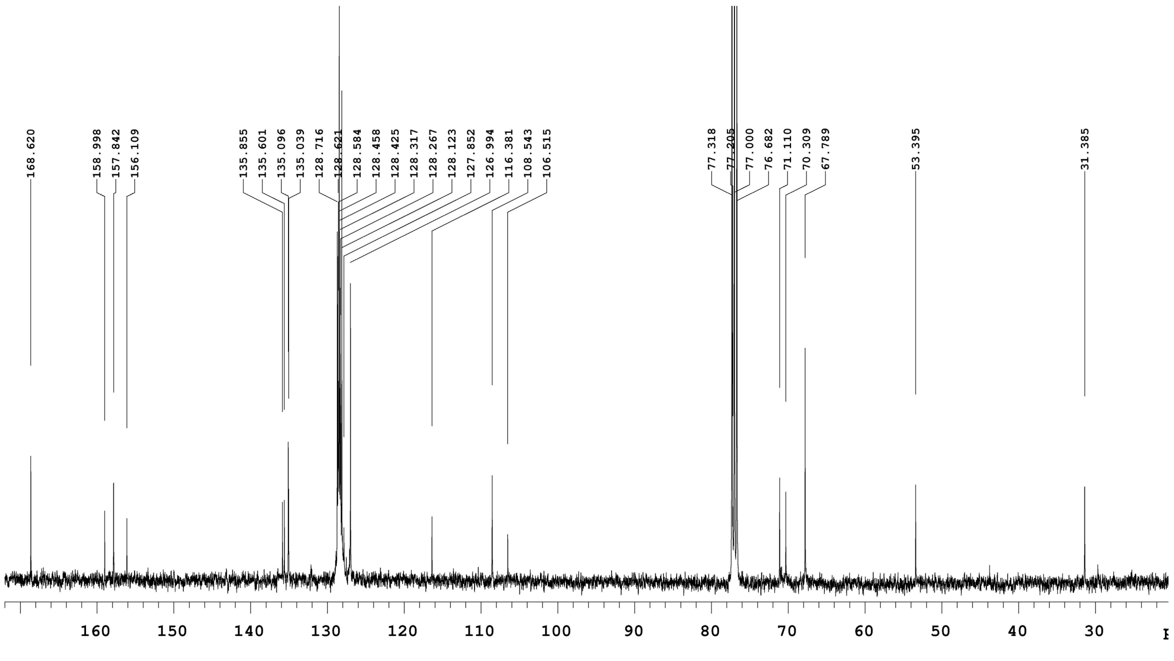

(**b**)

Figure S21. ^{1}H-NMR spectrum of dibenzyl 5-chloro-6,8-dibenzyloxy-3,4-dihydroisoquinolin-3,3-dicarboxylate **4k**.

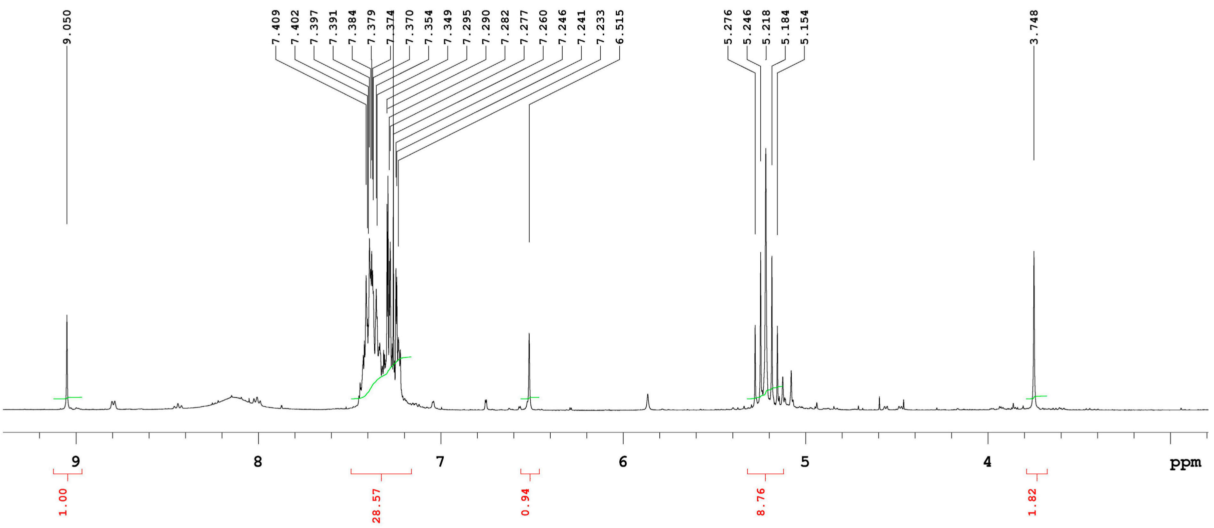

Figure S22. ^{1}H-NMR spectrum of dibenzyl 5-chloro-6,7-dimethoxy-3,4-dihydroisoquinolin-3,3-dicarboxylate **4l**.

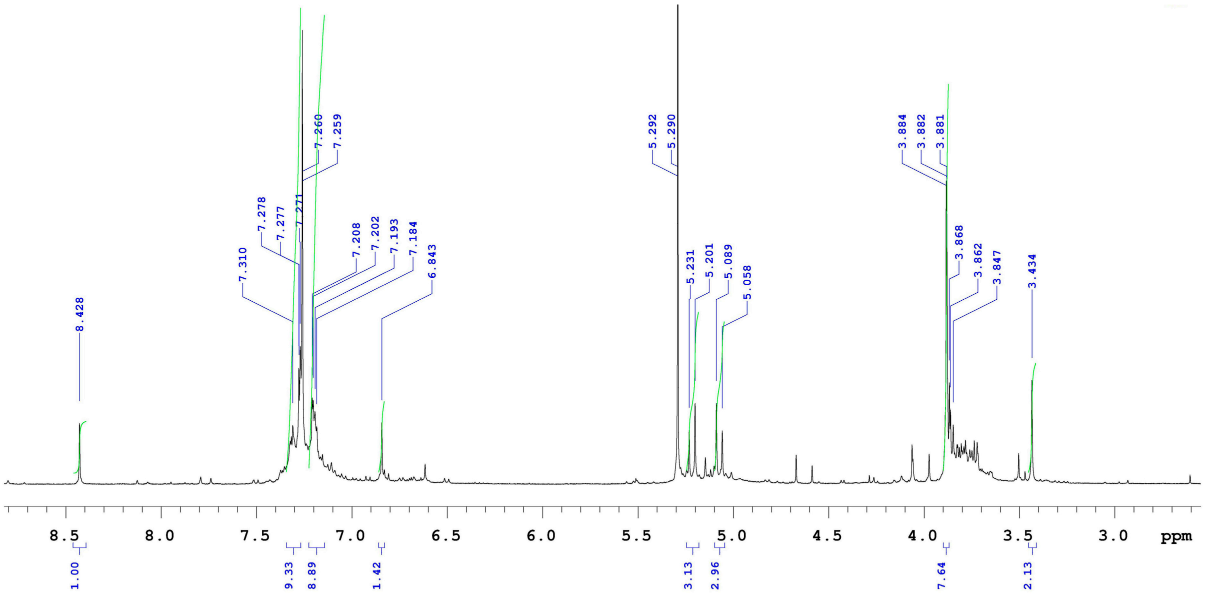

Figure S23. ^{1}H-NMR spectrum of 5-bromo-8-hydroxy-3,4-dihydroisoquinolin-3-carboxylic acid **5a**.

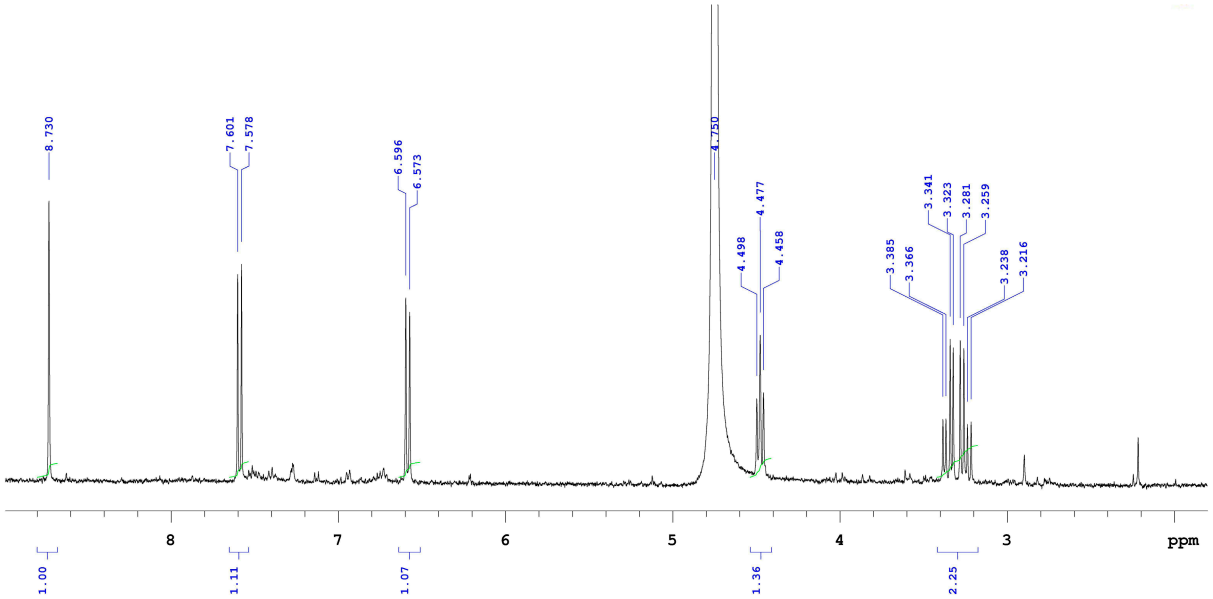

Figure S24. ^{1}H-NMR spectrum of 7-Bromo-8-hydroxy-3,4-dihydroisoquinolin-3-carboxylic acid **5b**.

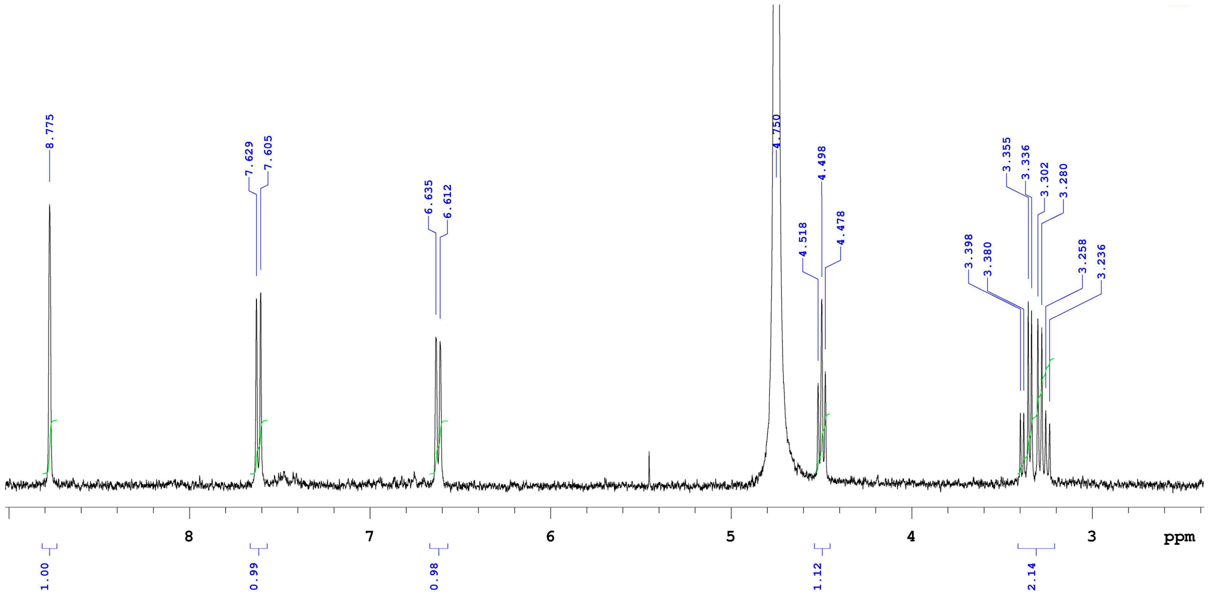

Figure S25. ^{1}H-NMR spectrum of 6,8-dihydroxy-3,4-dihydroisoquinolin-3-carboxylic acid **5c**.

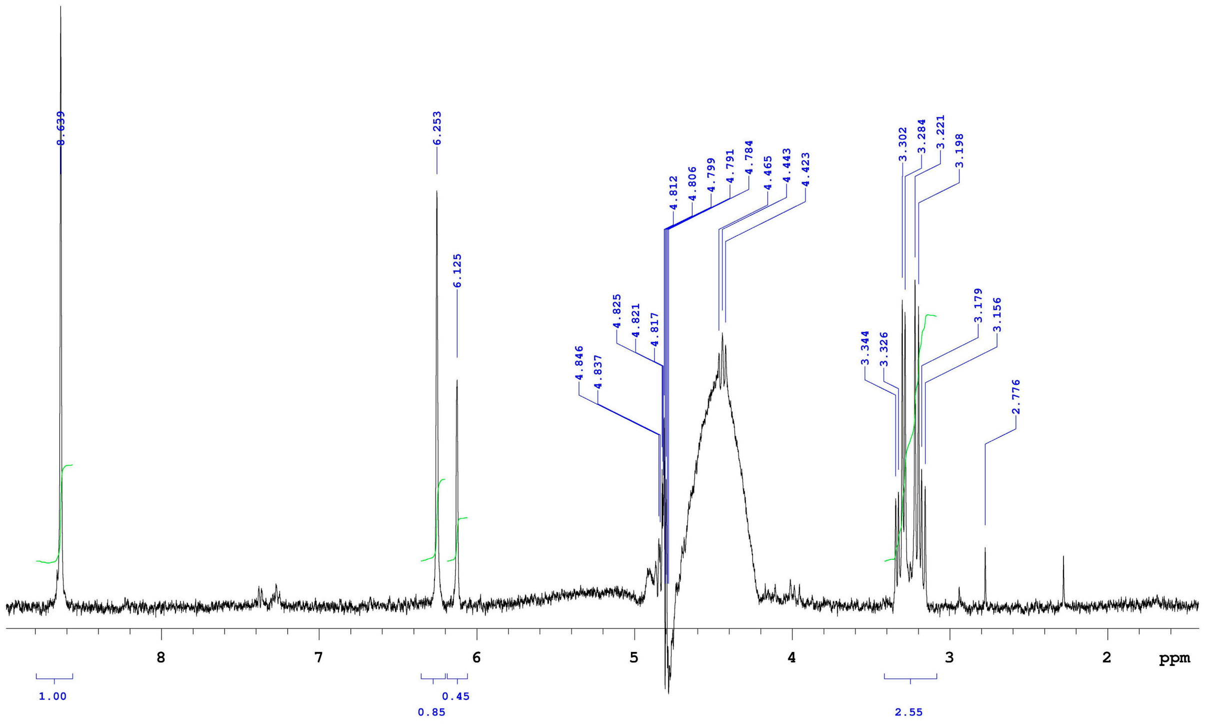

Figure S26. ^{1}H-NMR spectrum of 6,7-dihydroxy-8-iodo-3,4-dihydroisoquinolin-3-carboxylic acid **5d**.

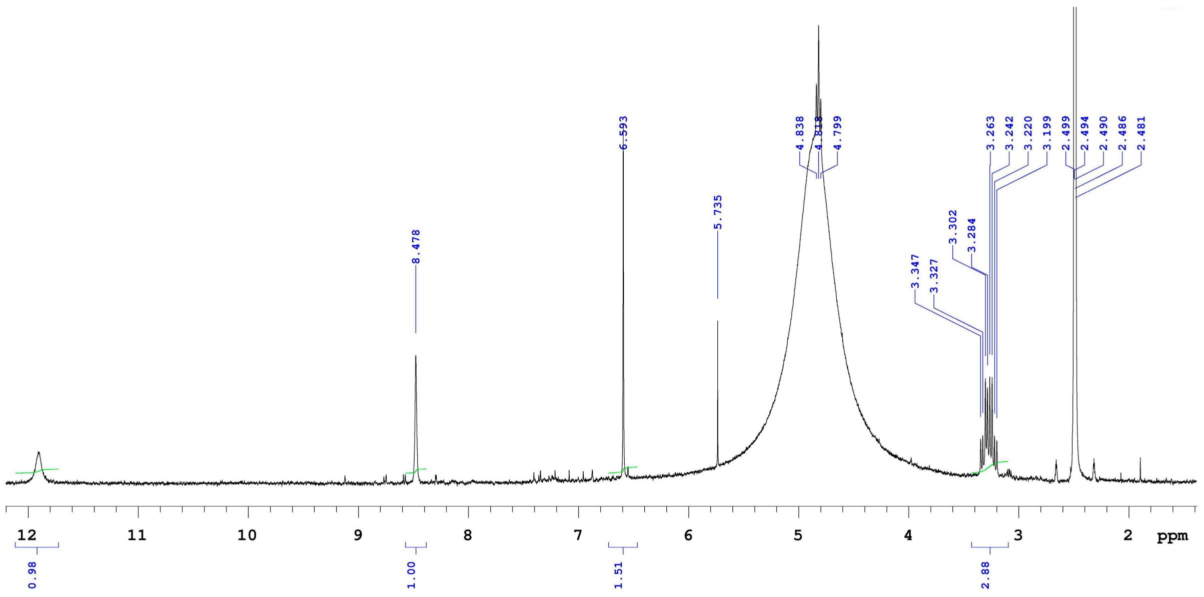

Figure S27. ^{1}H-NMR spectrum of 6,7-dihydroxy-3,4-dihydroisoquinolin-3-carboxylic acid **5e**.

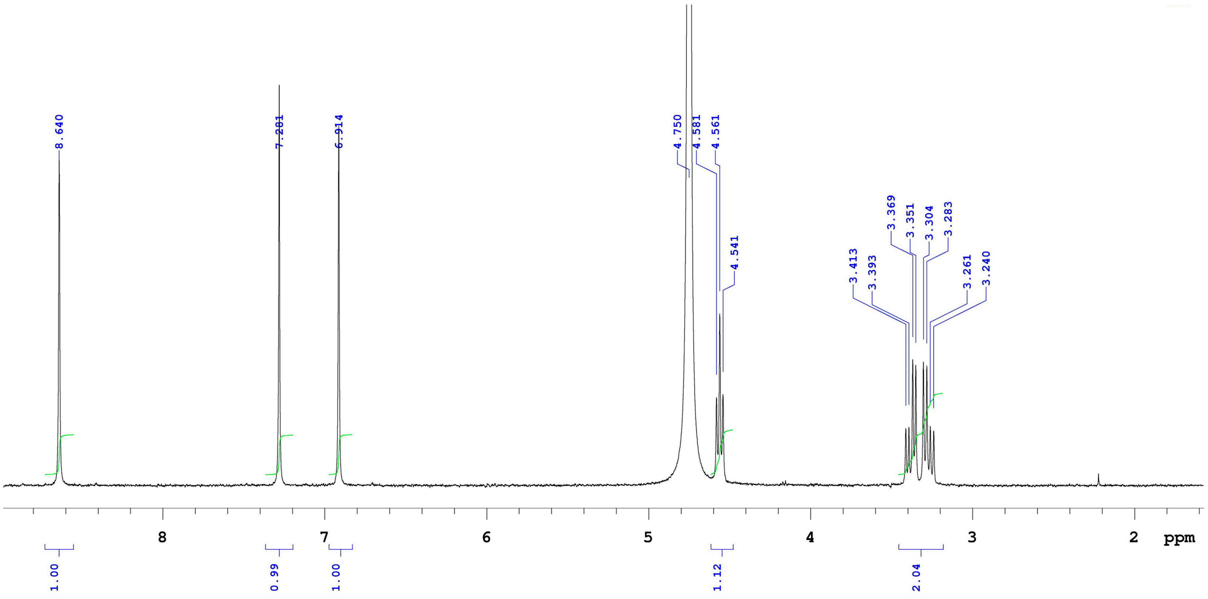

Figure S28. ^{1}H- (**a**) and ^{13}C-NMR (**b**) spectra of 8-bromo-5,6-dihydroxy-3,4-dihydroisoquinolin-3-carboxylic acid **5f**.

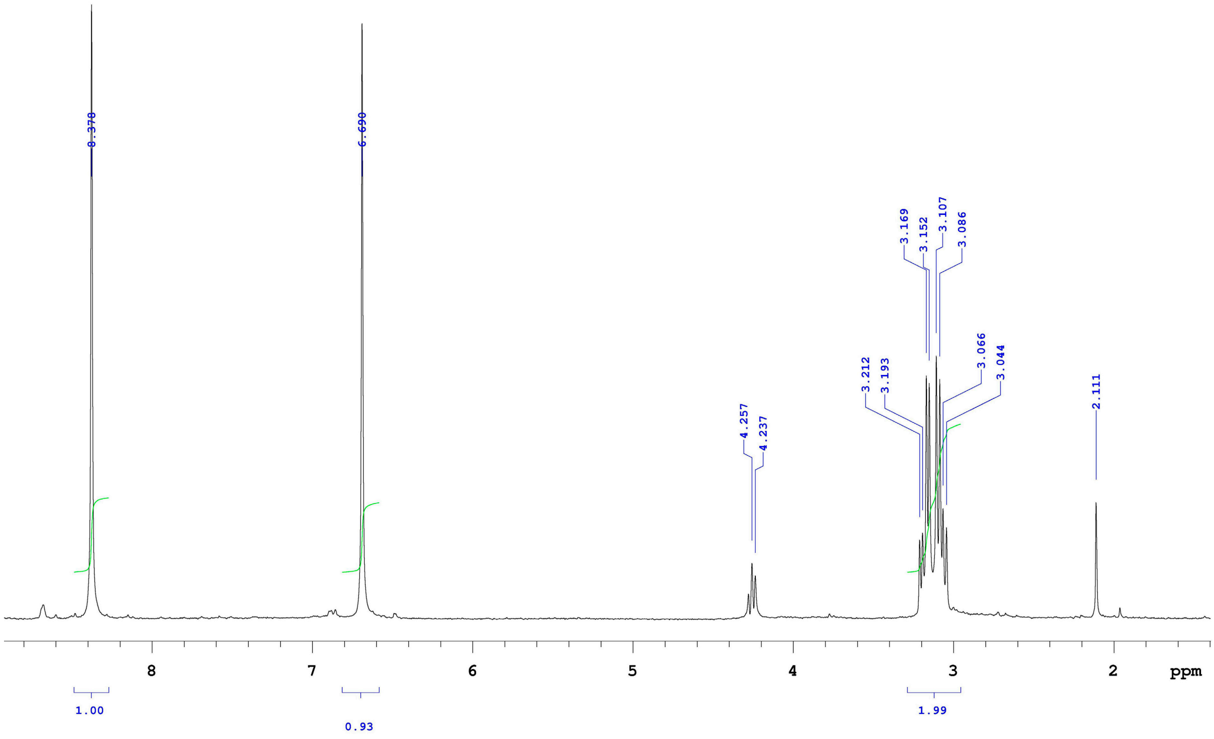

(**a**)

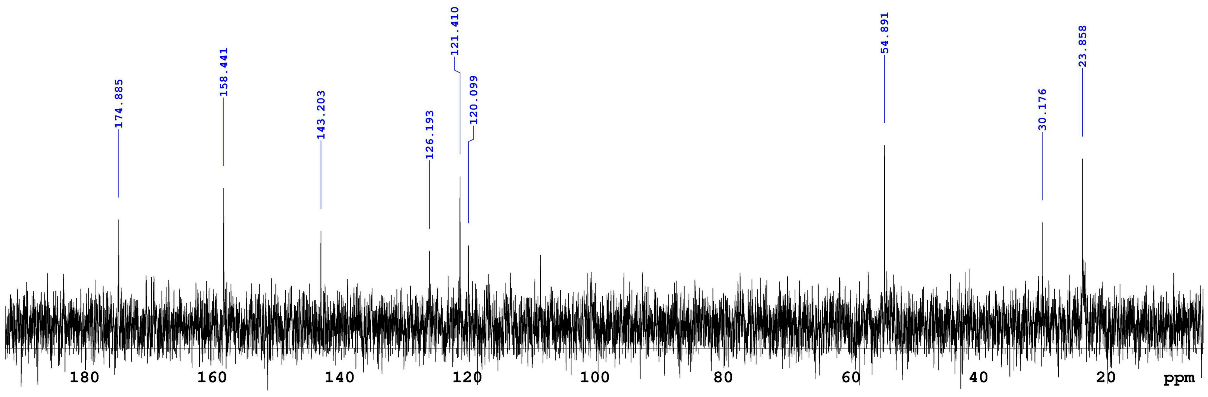

(**b**)

Figure S29. ^{1}H- (**a**) and ^{13}C-NMR (**b**) spectra of 8-bromo-6,7-dihydroxy-3,4-dihydroisoquinolin-3-carboxylic acid **5g**.

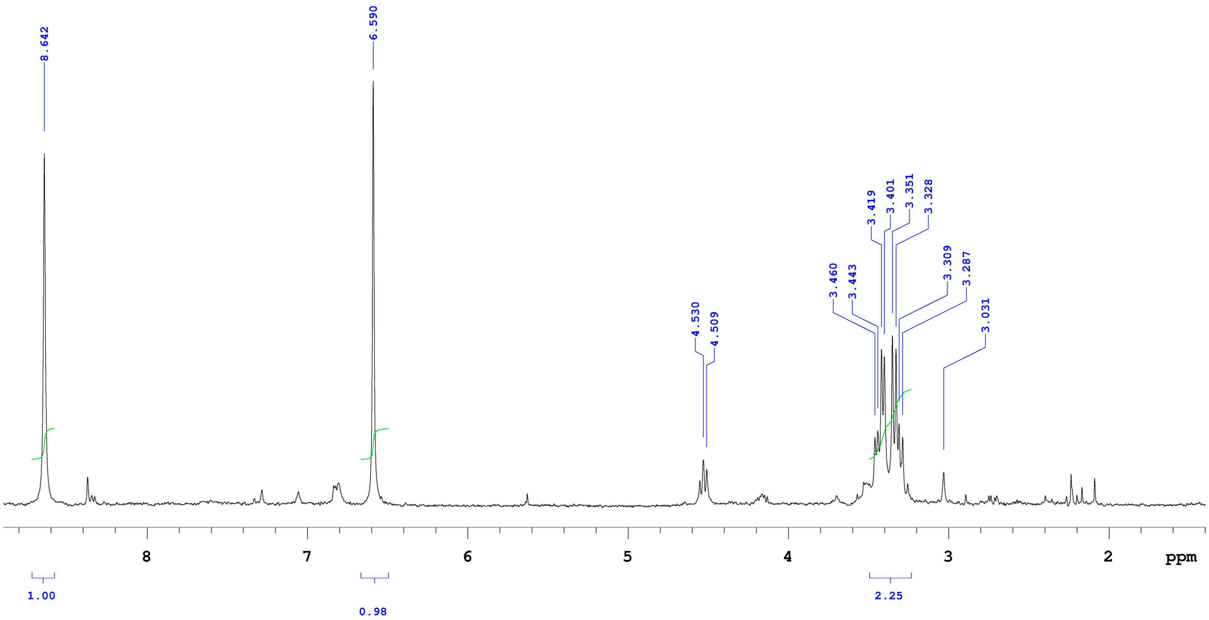

(**a**)

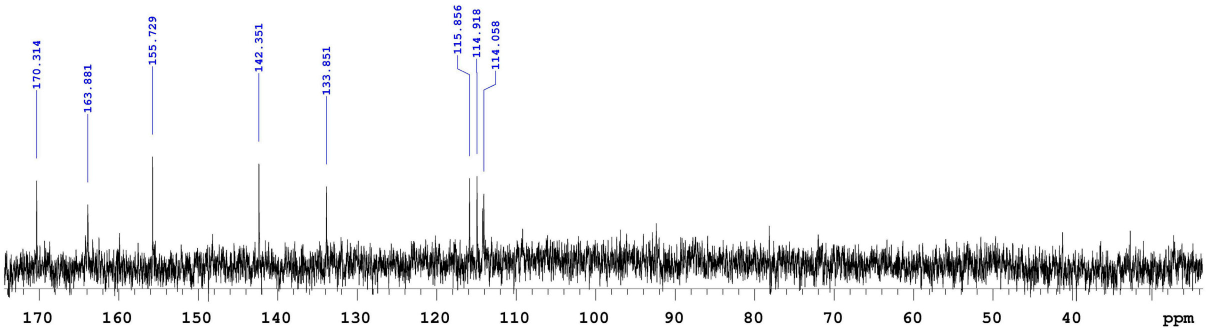

(**b**)

Figure S30. ^{1}H-NMR spectrum of 8-chloro-6,7-dihydroxy-3,4-dihydroisoquinolin-3-carboxylic acid **5h**.

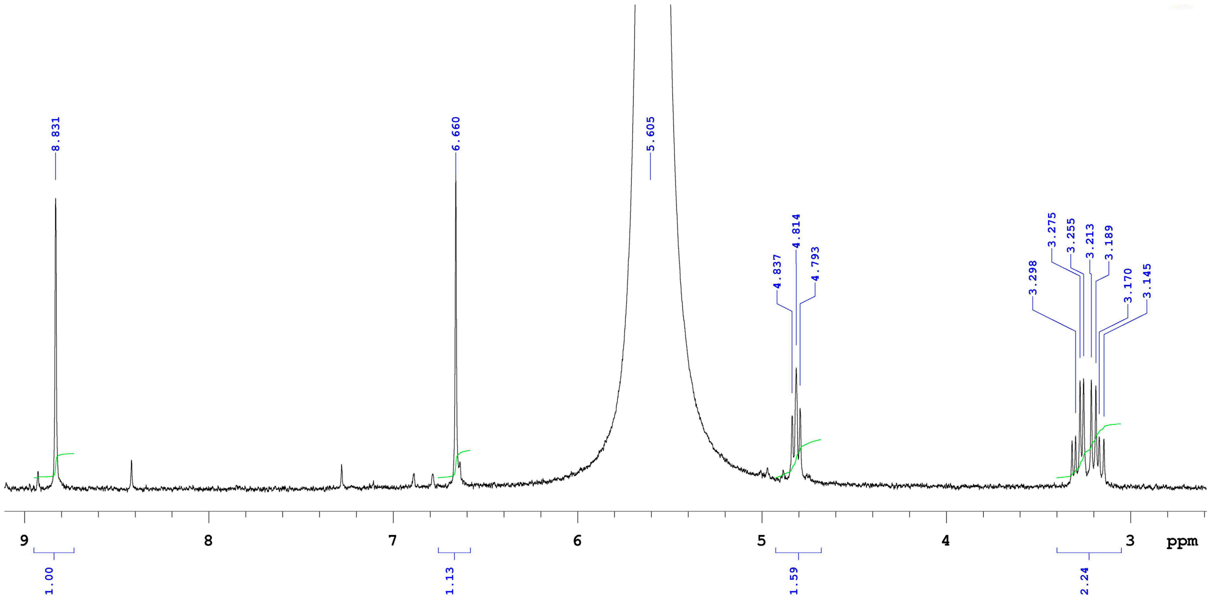

Figure S31. ^{1}H-NMR spectrum of 7,8-dihydroxy-6-methoxy-3,4-dihydroisoquinolin-3-carboxylic acid **5i**.

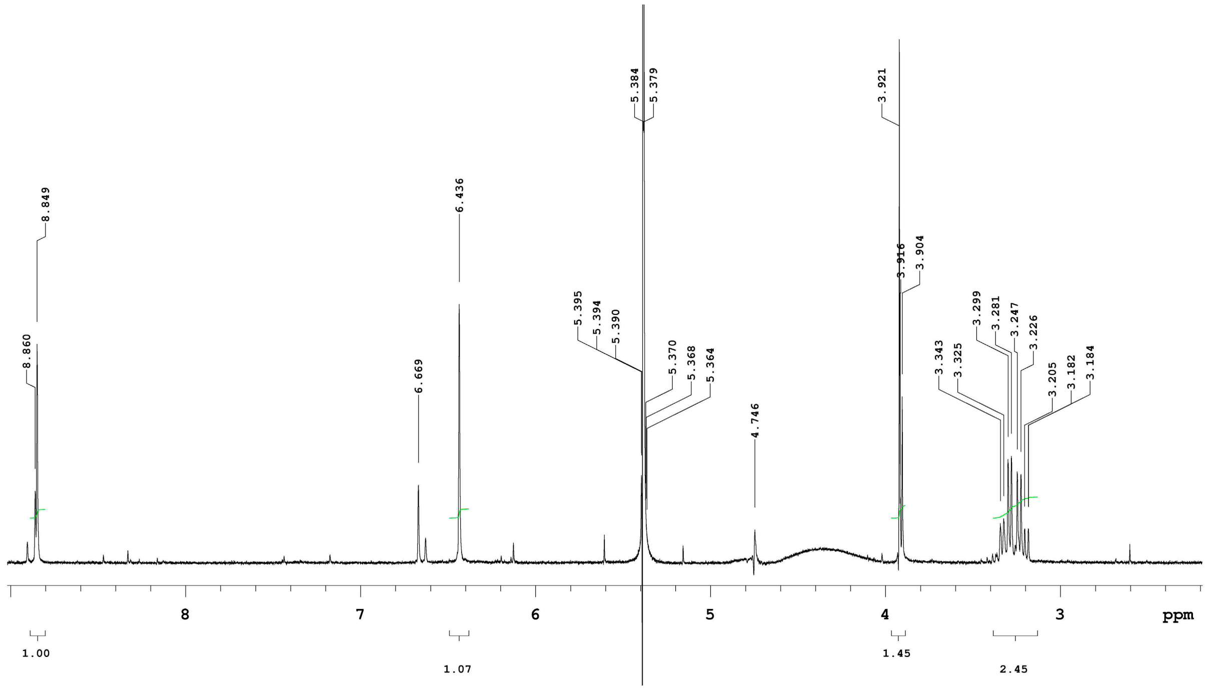

Figure S32. ^{1}H- (**a**) and ^{13}C-NMR (**b**) spectra of 7-bromo-6,8-dihydroxy-3,4-dihydroisoquinolin-3-carboxylic acid **5j**.

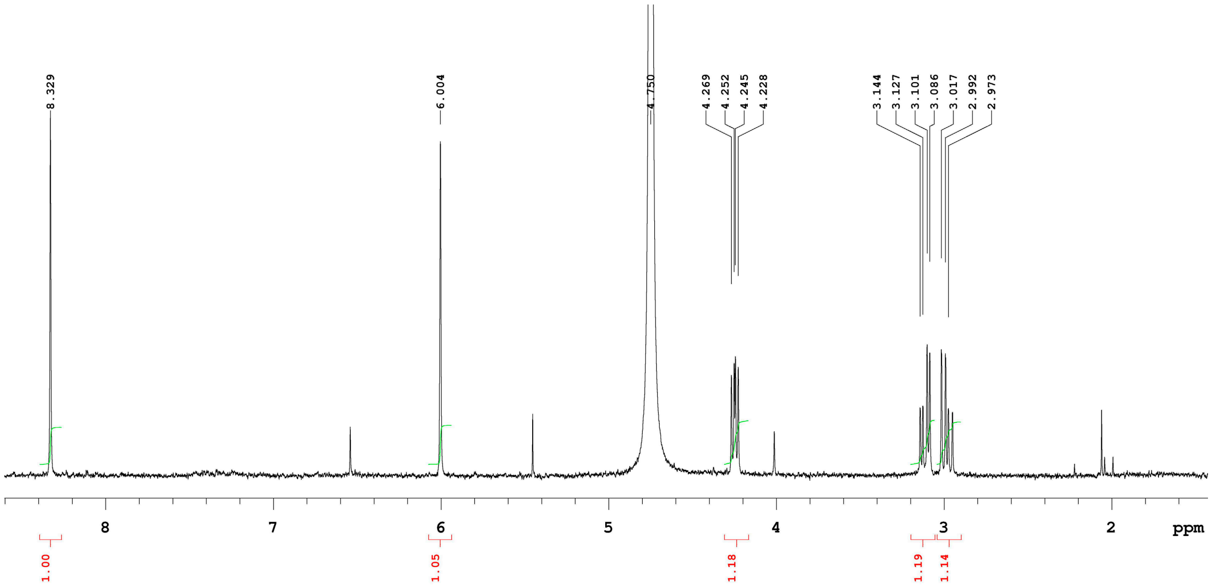

(**a**)

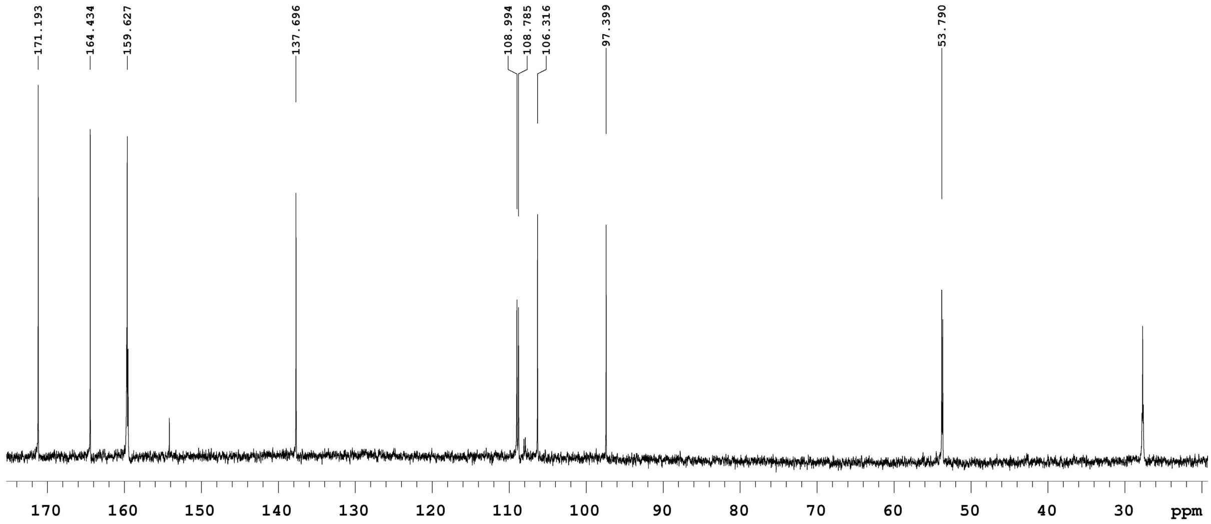

(**b**)

Figure S33. ^{1}H-NMR spectrum of 5-chloro-6,8-dihydroxy-3,4-dihydroisoquinolin-3-carboxylic acid **5k**.

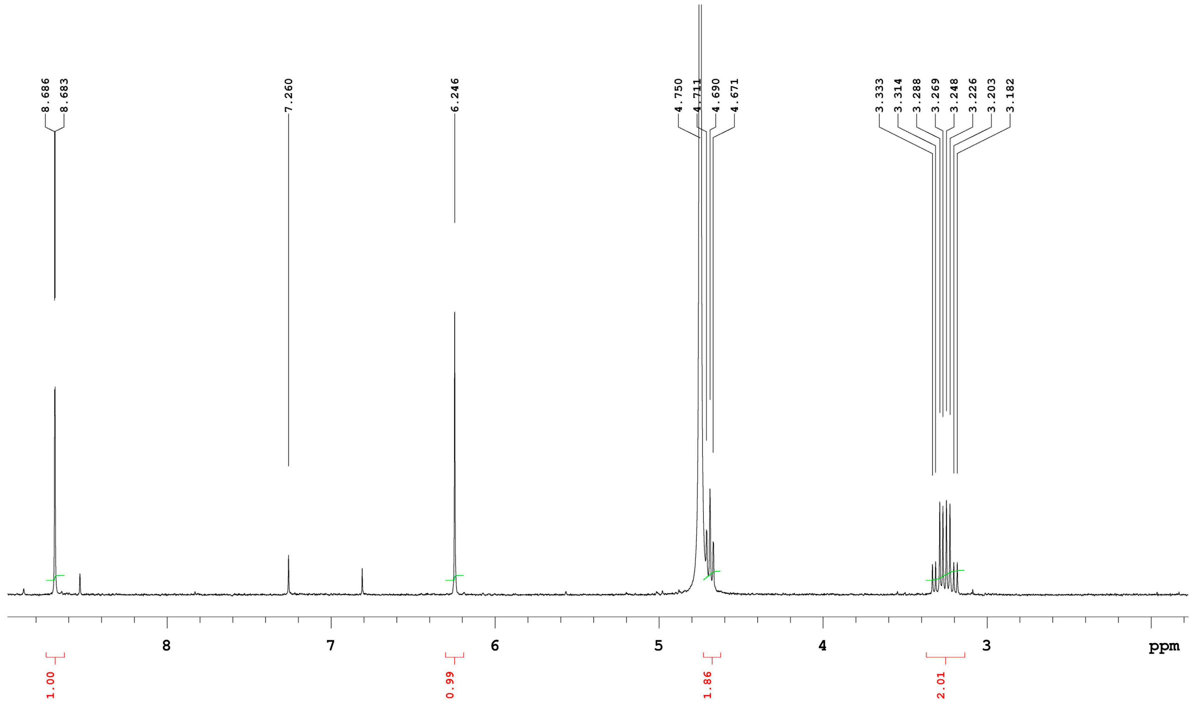

Figure S34. ^{1}H- (**a**) and ^{13}C-NMR (**b**) spectra of 5-chloro-6,7-dihydroxy-3,4-dihydroisoquinolin-3-carboxylic acid **5l**.

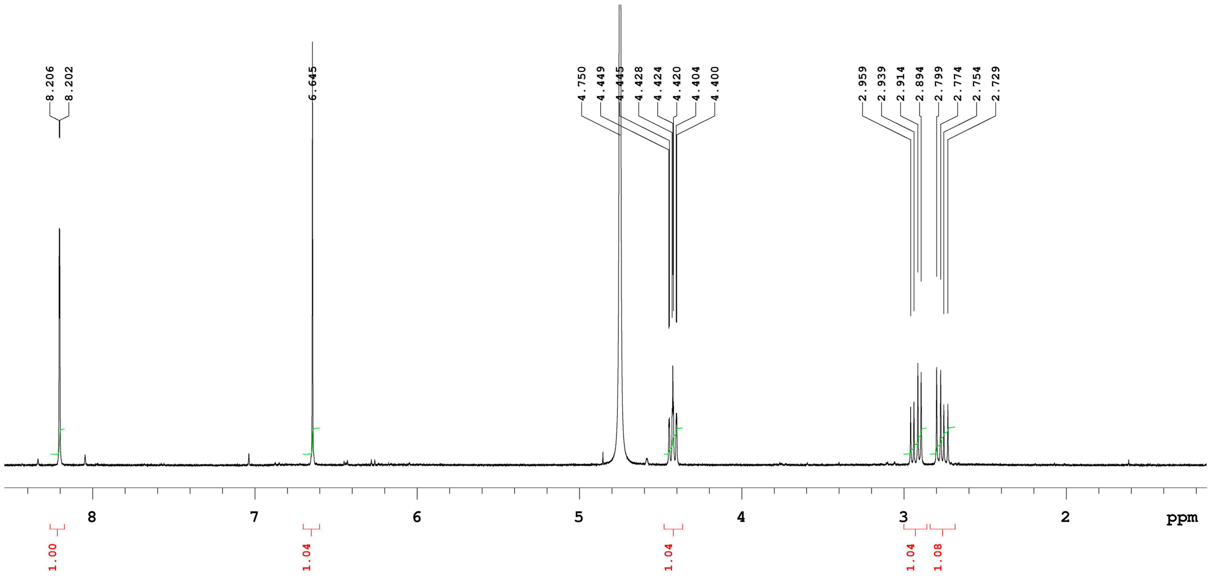

(**a**)

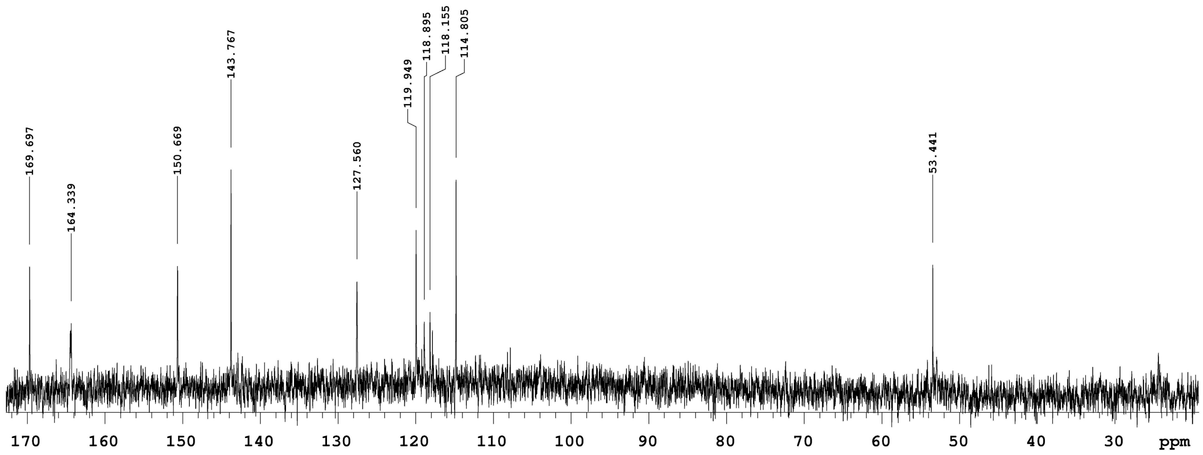

(**b**)

Figure S35. ^{1}H- (**a**) and ^{13}C-NMR (**b**) spectra of dibenzyl 2-[2-(3,4-dimethoxyphenyl)ethyl]-2-formamidomalonate **7**.

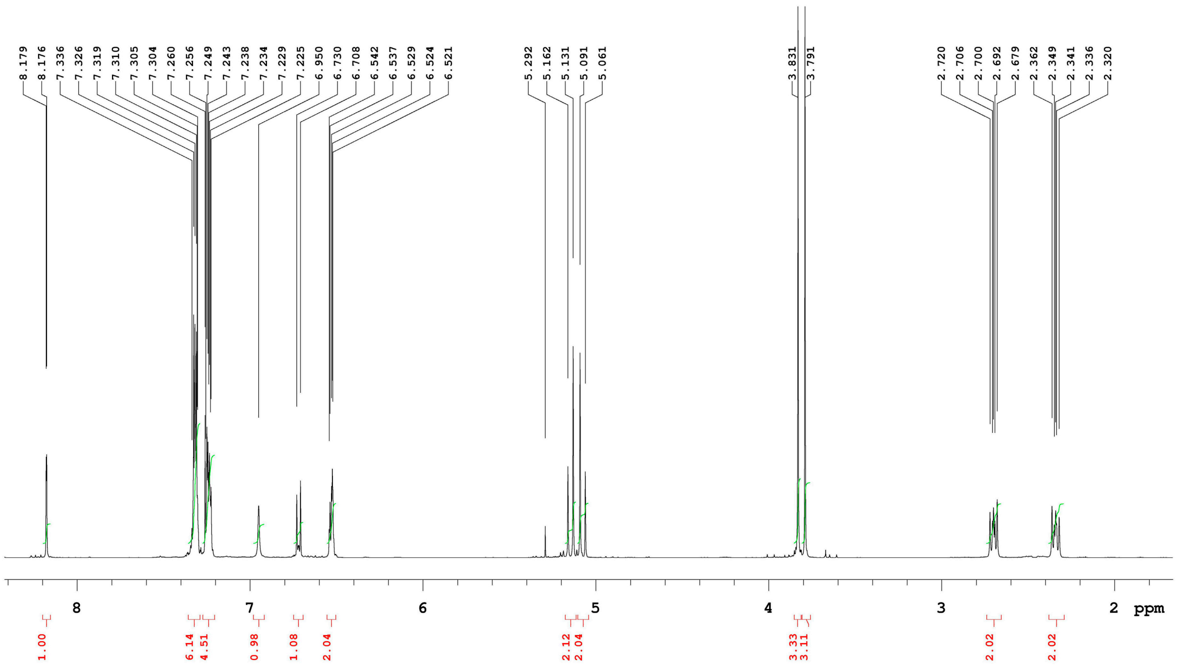

(**a**)

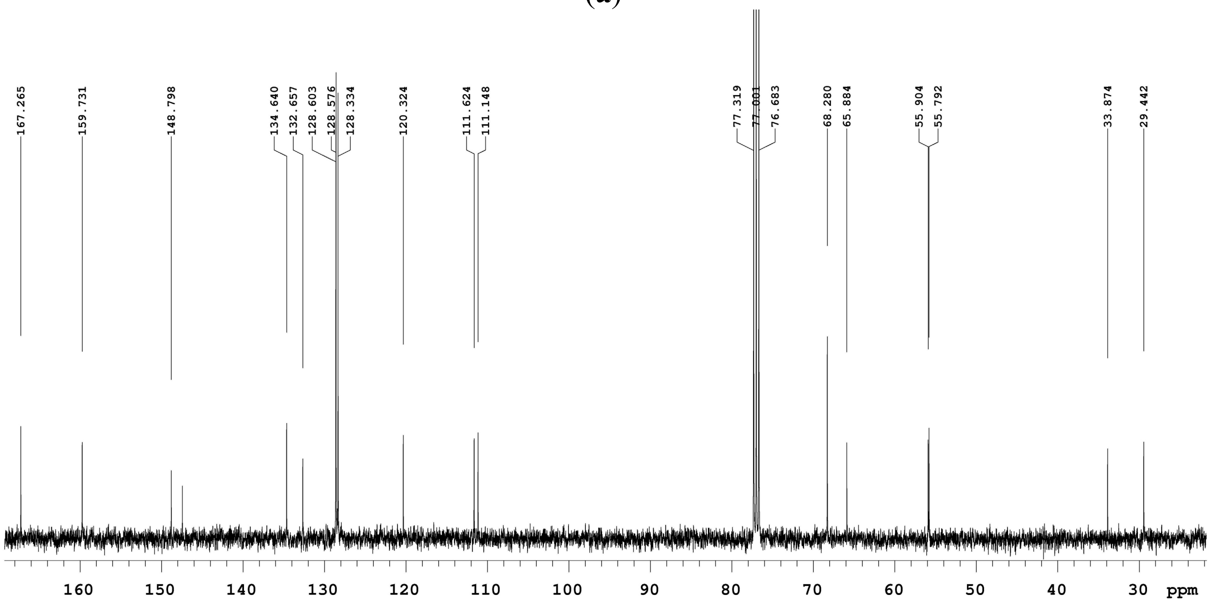

(**b**)

Figure S36. ^{1}H- (**a**) and ^{13}C-NMR (**b**) spectra of dibenzyl 7,8-dimethoxy-4,5-dihydro-3H-2-benzazepine-3,3-dicarboxylate **8**.

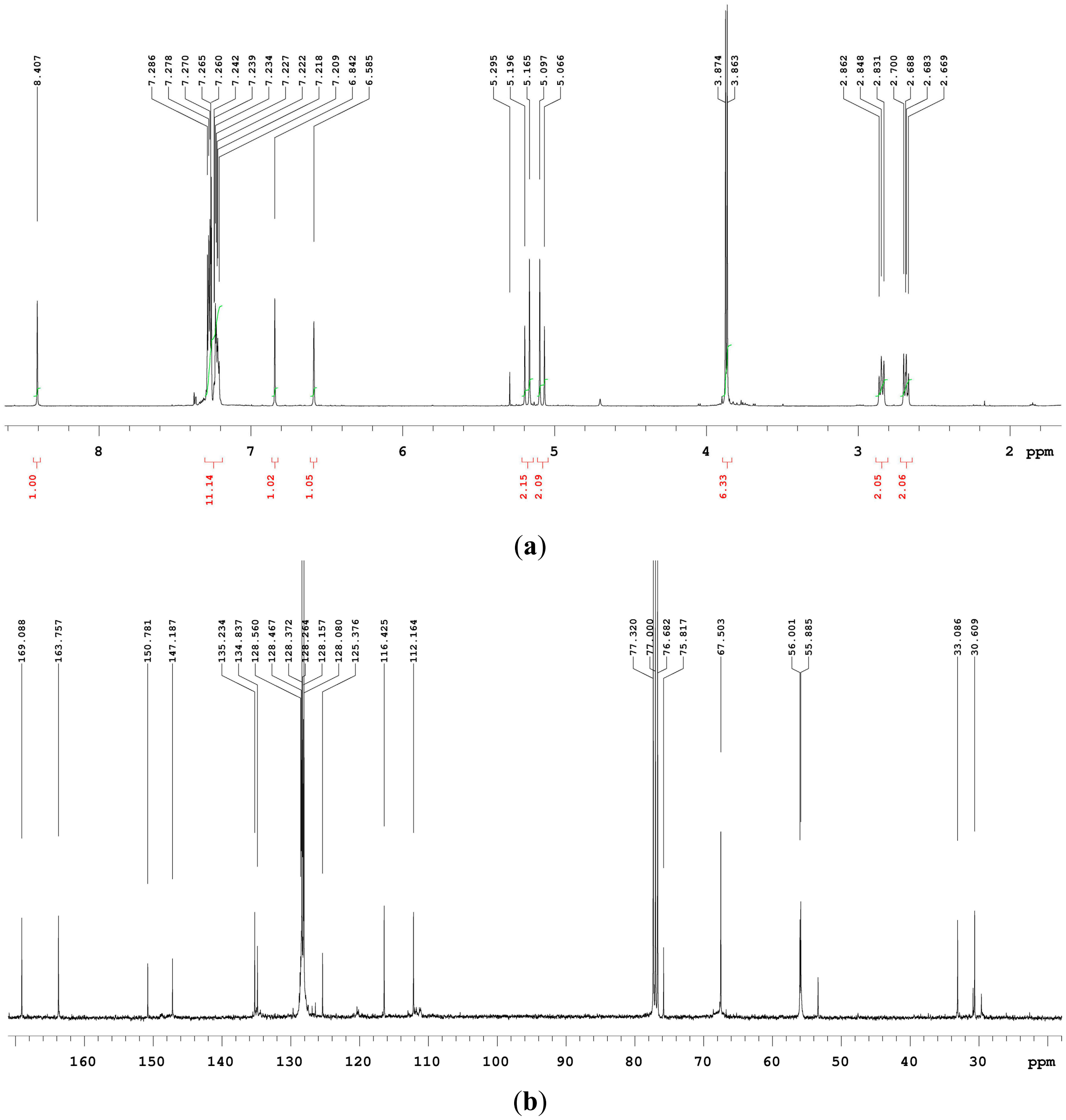

Figure S37. ^{1}H-NMR spectra of 7,8-dihydroxy-4,5-dihydro-3H-2-benzazepine-3-carboxylic acid **9**.

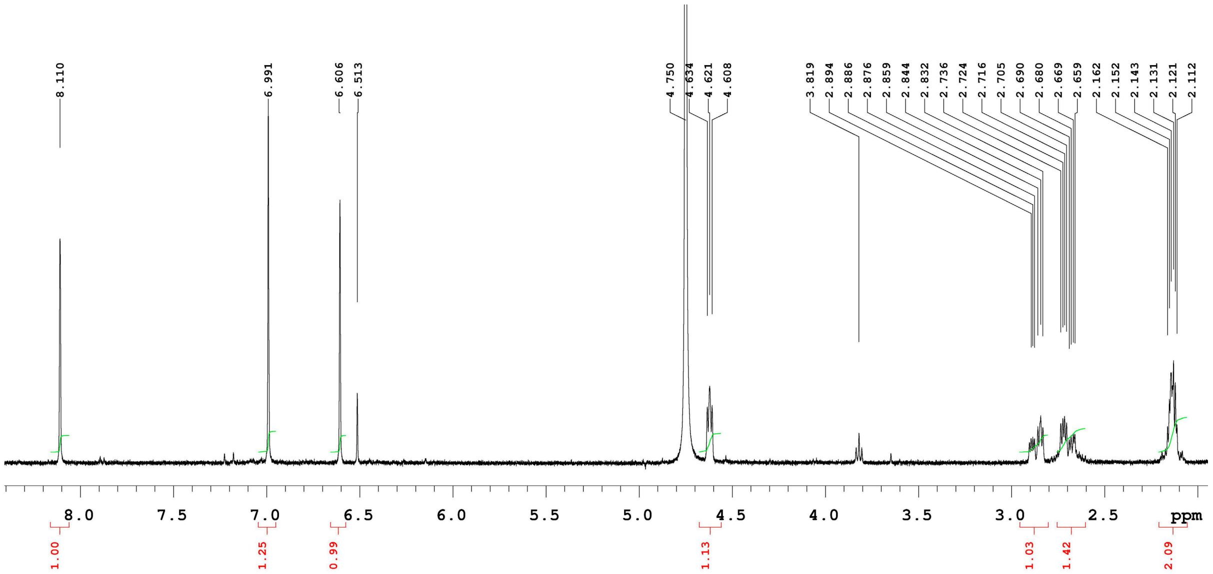

Figure S38. ^{1}H- (**a**) and ^{13}C-NMR (**b**) spectra of (*S*)-benzyl 2-(3,4-dibenzyloxy)benzyl-2-formamidopropanoate **11**.

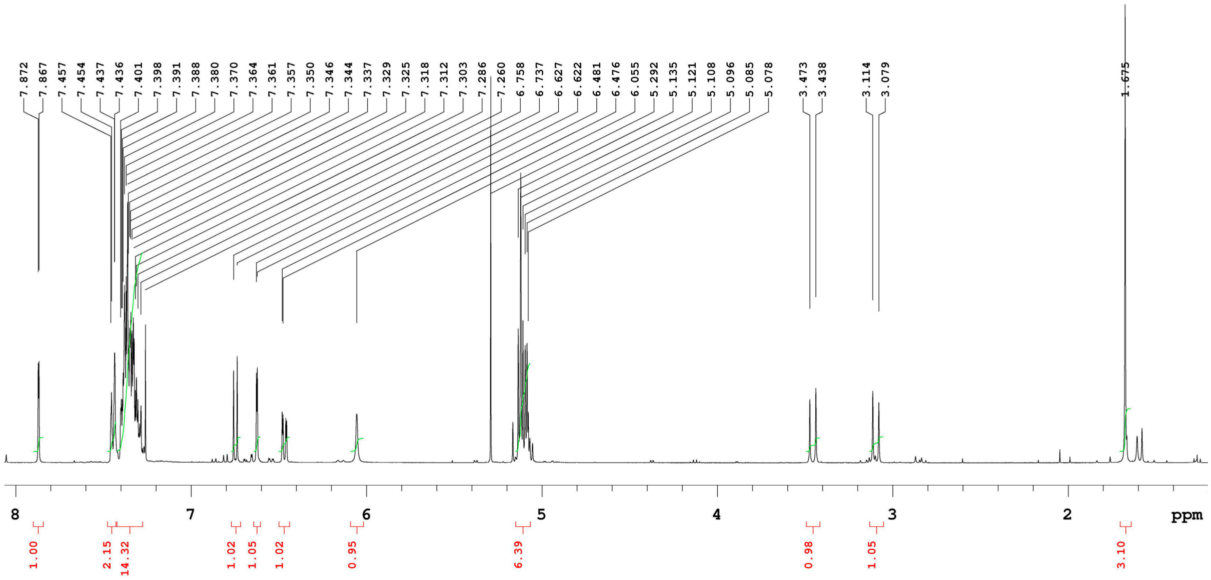

(**a**)

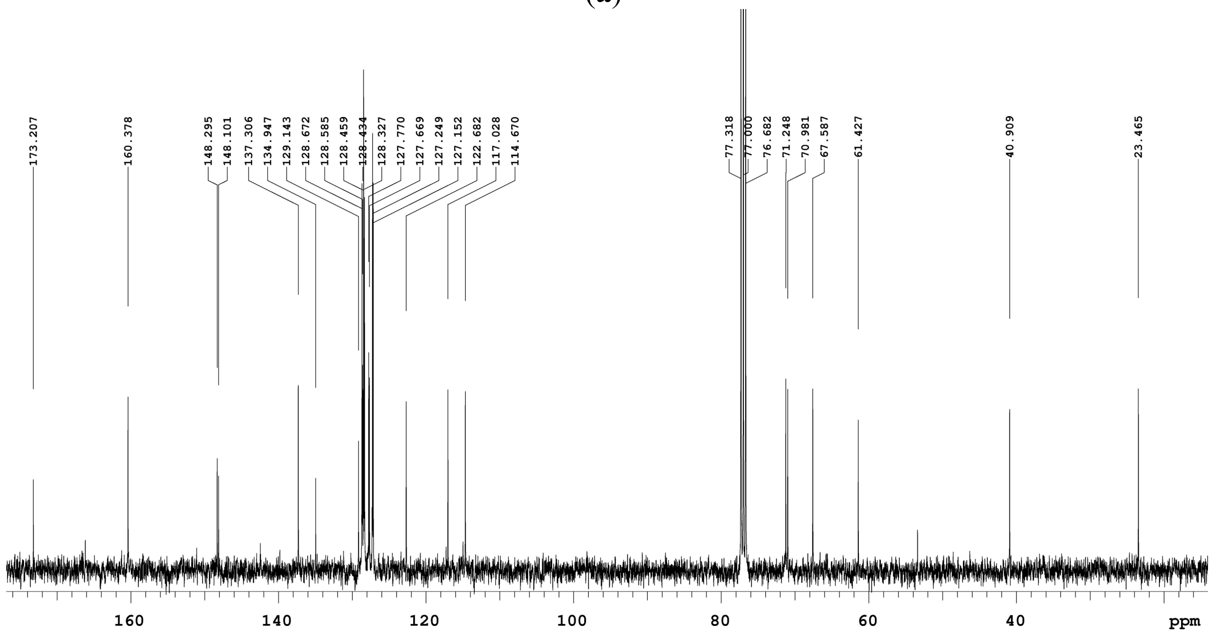

(**b**)

Figure S39. ^{1}H- (**a**) and ^{13}C-NMR (**b**) spectra of (*S*)-benzyl-6,7-dibenzyloxy-3-methyl-3,4-dihydroisoquinolin-3-carboxylate **12**.

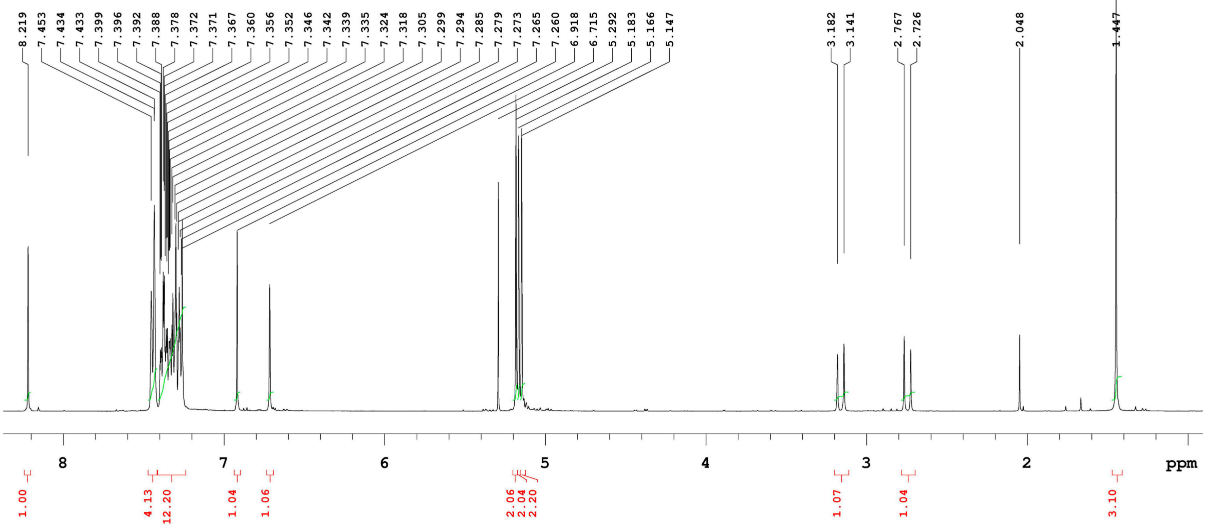

(**a**)

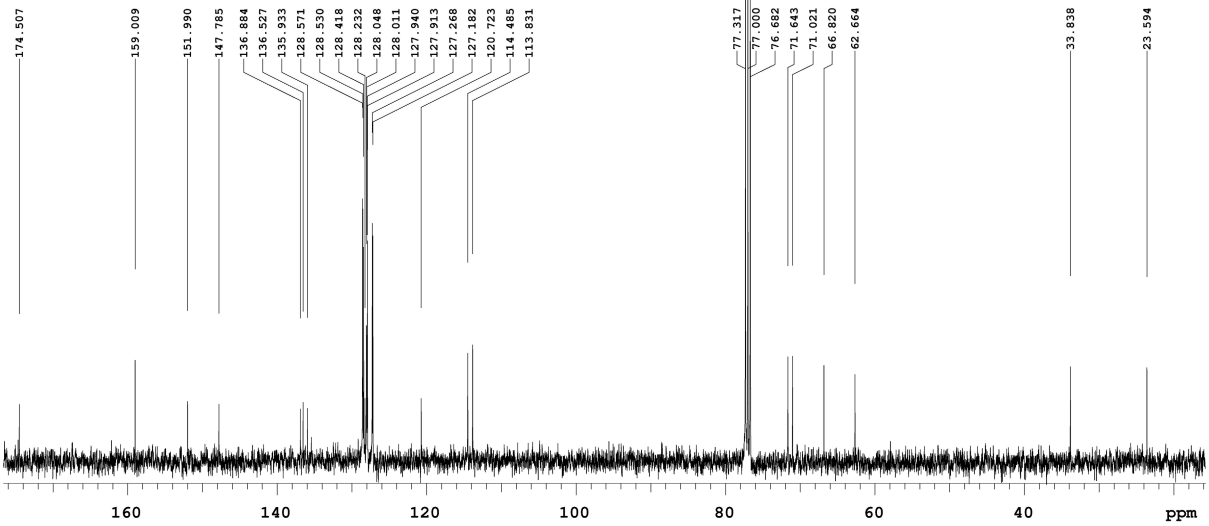

(**b**)

Figure S40. ^{1}H- (**a**) and ^{13}C-NMR (**b**) spectra of (*S*)-6,7-dihydroxy-3-methyl-3,4-dihydroisoquinolin-3-carboxylic acid **13**.

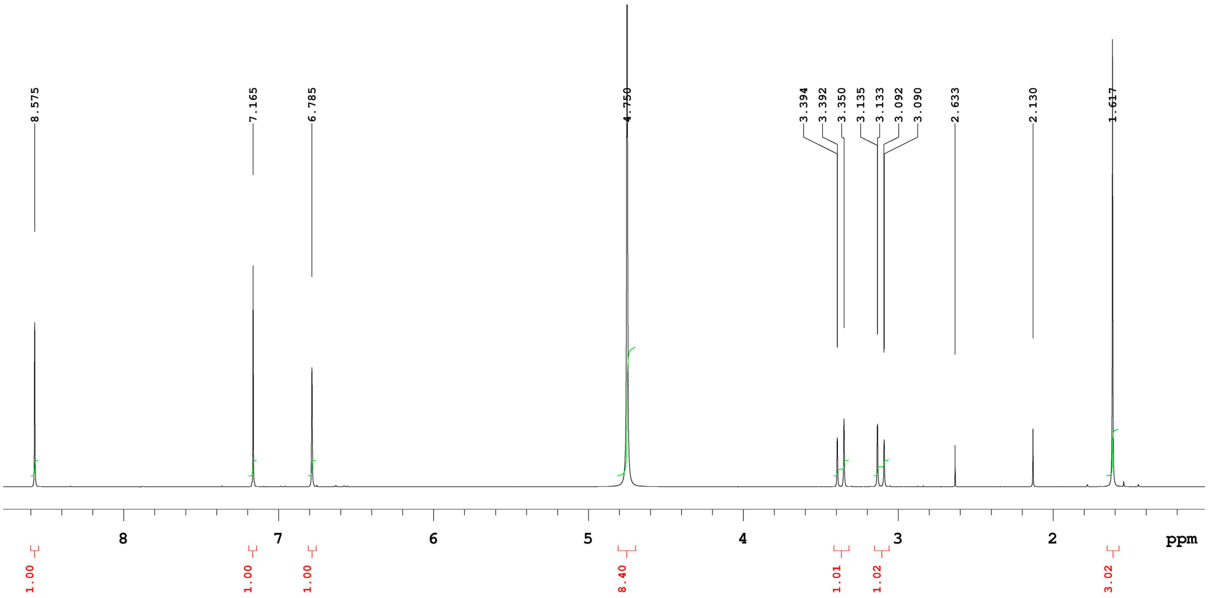

(**a**)

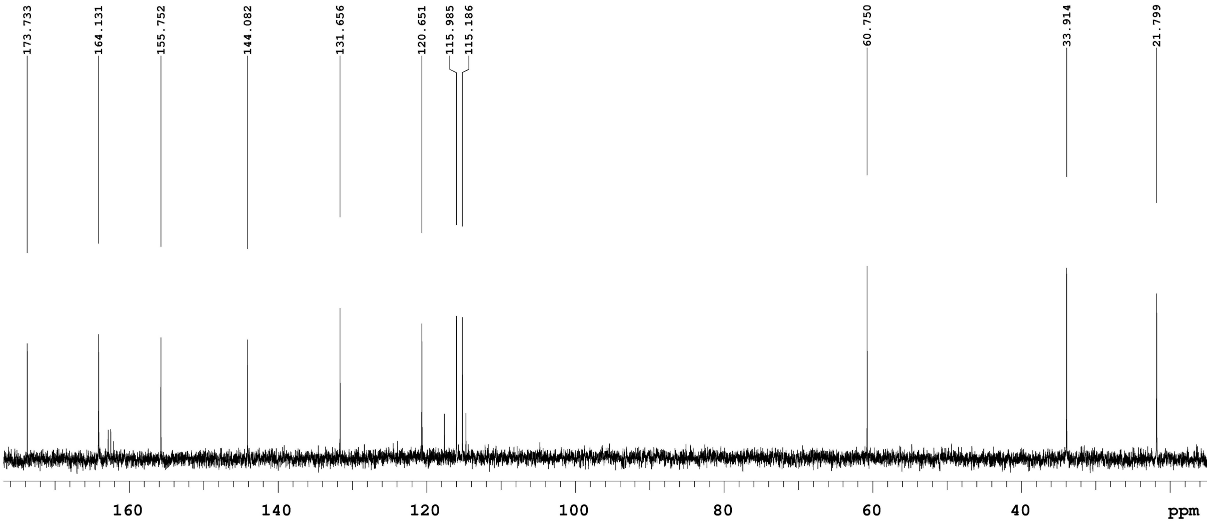

(**b**)

Figure S41. ^{1}H- (**a**) and ^{13}C-NMR (**b**) spectra of benzyl 1-chloro-7,8-dibenzyloxy-4-methyl-2-oxo-1,4,5,9b-tetrahydro-2H-azeto[2,1-a]isoquinoline-4-carboxylate **14**.

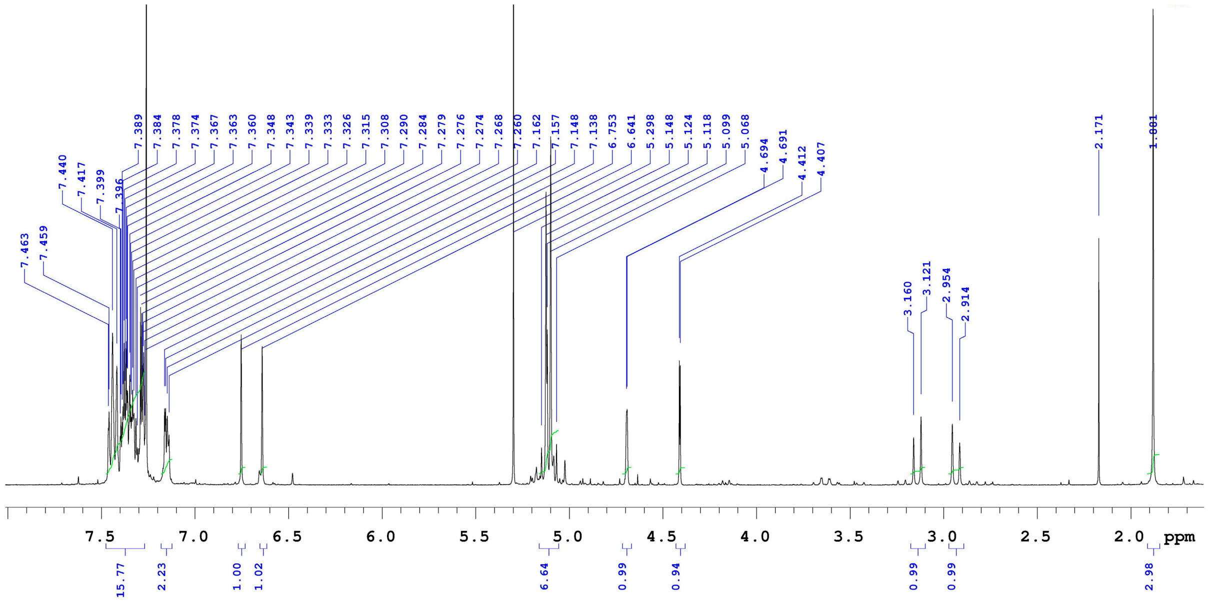

(**a**)

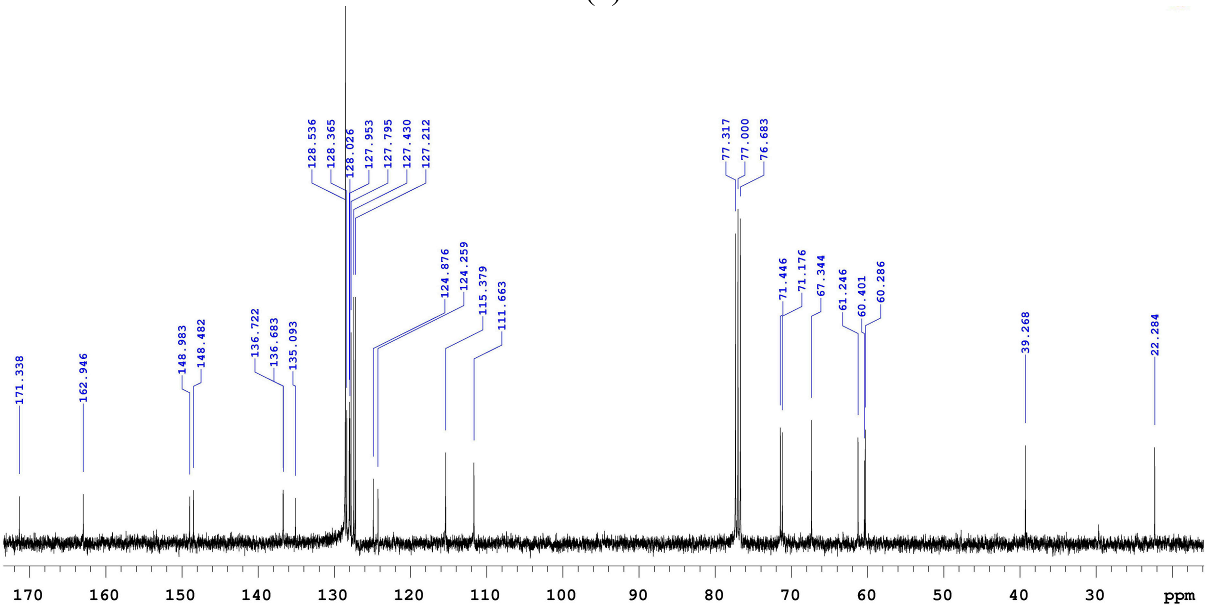

(**b**)

Figure S42. ^{1}H- (**a**) and ^{13}C-NMR (**b**) spectra of 1-chloro-7,8-dihydroxy-4-methyl-2-oxo-1,4,5,9b-tetrahydro-2H-azeto[2,1-a]isoquinoline-4-carboxylic acid **15**.

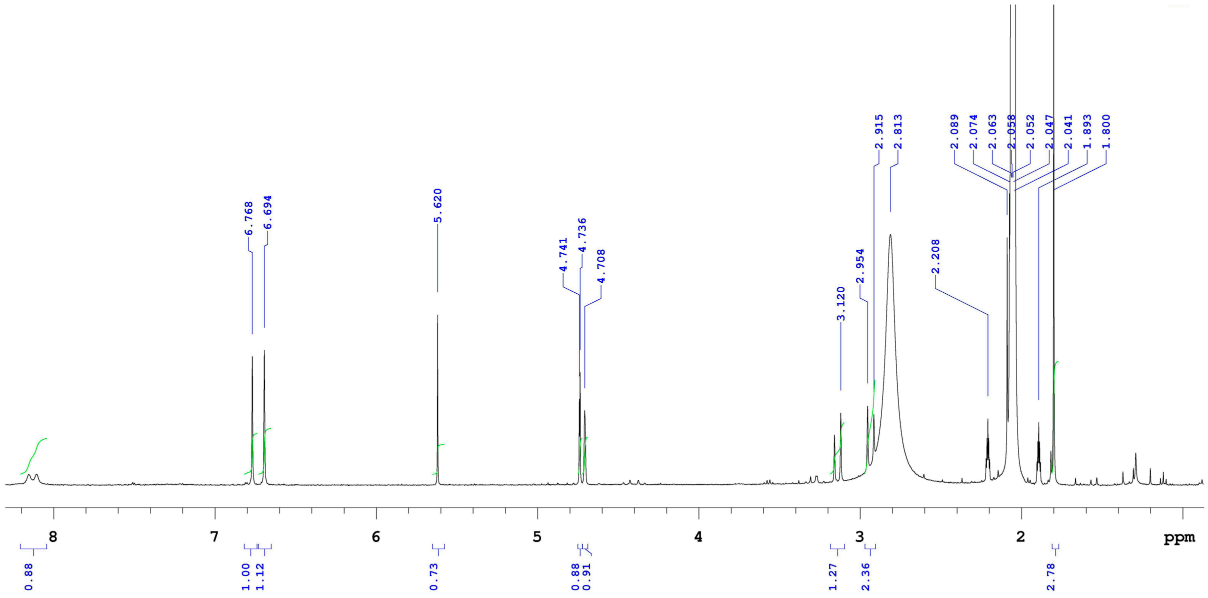

(**a**)

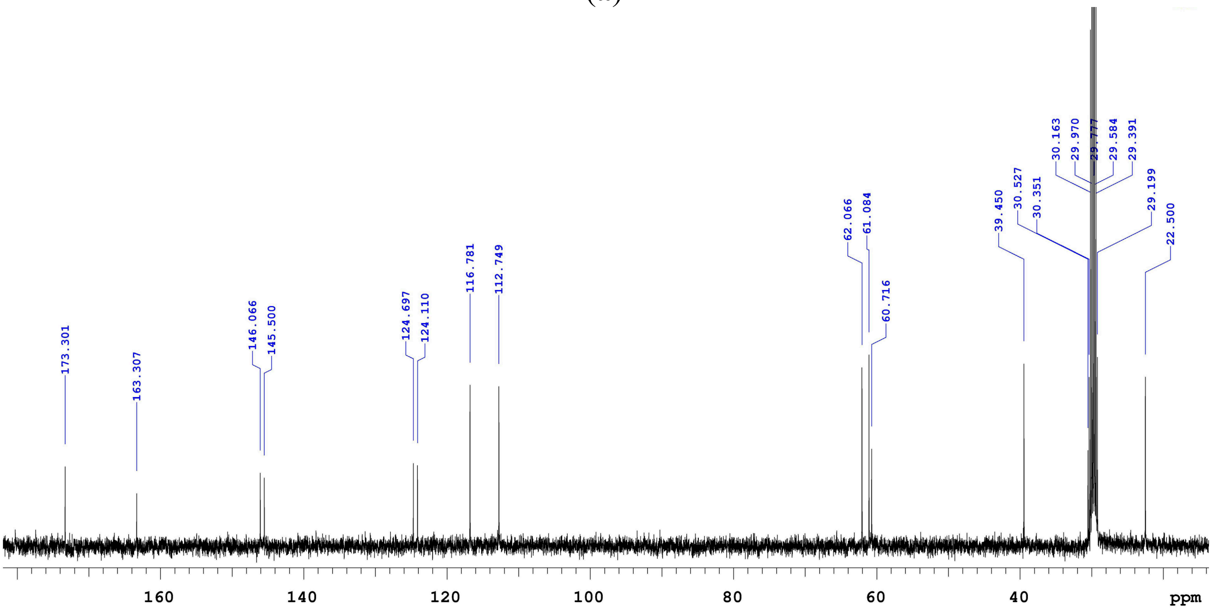

(**b**)
